# First-line benmelstobart plus anlotinib and chemotherapy in advanced or metastatic/recurrent esophageal squamous cell carcinoma: a multi-center phase 2 study

**DOI:** 10.1038/s41392-024-02008-7

**Published:** 2024-11-08

**Authors:** Ning Li, Jin Xia, Xiaohui Gao, Jianwei Zhou, Yonggui Hong, Donghai Cui, Xuesong Zhao, Tao Wu, Yanzhen Guo, Junsheng Wang, Suxia Luo

**Affiliations:** 1 Department of Gastrointestinal Oncology, The Affiliated Cancer Hospital of Zhengzhou University & Henan Cancer Hospital, Zhengzhou, China; 2 Department of Medical Oncology, Anyang Tumor Hospital & The Affiliated Anyang Tumor Hospital of Henan University of Science and Technology, Anyang, Henan China; 3 Department of Respiratory Oncology, The First Affiliated Hospital of Henan University of Science and Technology, Luoyang, Henan China; 4 Department of Oncology, Henan Provincial People’s Hospital, Zhengzhou, Henan China; 5 Department of Tumor Radiotherapy, The People’s Hospital of Anyang City, Anyang, Henan China

**Keywords:** Gastrointestinal cancer, Clinical trials

## Abstract

Although first-line immunochemotherapy has improved prognosis for patients with advanced esophageal squamous cell carcinoma (ESCC), more effective strategies still require further investigation. This multi-center, phase II study (ClinicalTrials.gov NCT05013697) assessed the feasibility of benmelstobart (a novel PD-L1 inhibitor) plus anlotinib (multitargeted TKI) and chemotherapy in advanced or metastatic/recurrent ESCC. Eligible patients received 4–6 cycles (21-day) of benmelstobart (1200 mg), anlotinib (10 mg) plus paclitaxel (135 mg/m^2^)/cisplatin (60–75 mg/m^2^), then maintained with benmelstobart and anlotinib. Primary endpoint was progression-free survival (PFS) assessed according to RECIST v1.1. Secondary endpoints were tumor response, overall survival (OS), and safety assessed by adverse events (AEs). From September 2021 to November 2023, 50 patients were enrolled and received study treatment. With median follow-up of 23.7 months as of April 1, 2024, median PFS was 14.9 months (95% CI, 11.4-not estimable [NE]) and the 1-year PFS was 58.5% (95% CI, 41.9%–71.9%). Among 50 patients, confirmed objective response rate was 72.0% and disease control rate was 84.0%. Median duration of response of 36 responders was 16.2 months (95% CI, 10.2-NE). At the cutoff date, 31 patients remained alive; median OS was not reached (95% CI, 13.2 months-NE) with 1-year OS of 74.8% (95% CI, 59.8%–84.8%). Forty-six (92.0%) patients reported treatment-related AEs, with 37 (74.0%) were grade ≥3. Overall, benmelstobart plus anlotinib and chemotherapy showed promising efficacy and acceptable toxicity in advanced or metastatic/recurrent ESCC.

## Introduction

Esophageal squamous cell carcinoma (ESCC) accounts for approximately 90% of esophageal cancer and is particularly prevalent in Central to East Asia.^1^ Unfortunately, due to its highly aggressive behavior and the lack of early symptoms, most ESCC cases are advanced stage at diagnosis.^2,3^ Combination chemotherapy currently remains the standard first-line strategy for advanced ESCC, but with dismal survival.^4^

With the revolution of immuno-oncology therapies, several pivotal trials, such as CheckMate 648, KEYNOTE-590, and JUPITER-06,^5–7^ have established immunochemotherapy as the new standard for first-line treatment of this disease. Nevertheless, such combinations only provided limited clinical benefits, demonstrating overall survival (OS) ranging from 12 to 17 months.^5–7^ Notably, immunochemotherapy mainly benefits a subset of PD-L1-positive ESCC patients,^5,8^ potentially because of the immune evasion caused by vascular abnormalities.^9^

Given the highly angiogenic nature of ESCC and the synergistic effects of immunotherapy and antiangiogenic agents,^10,11^ numerous efforts are going to address the above issues with combination strategies. Current efforts mainly put the focus on PD-1 inhibitors-based triple regimens, such as the LEAP-014 trial, which evaluates pembrolizumab combined with lenvatinib and chemotherapy,^12^ and another phase II study assessing PD-1 blockades combined with anlotinib.^13^ However, the definitive benefits of these combinations have yet to be fully established, pending results from ongoing trials. In the realm of PD-L1 inhibitors, only the ALTER-E003 study has reported preliminary response data for first-line treatment with benmelstobart plus anlotinib.^14^ Notably, anti-PD-L1-based triple regimens, particularly involving multitargeted tyrosine kinase inhibitors (TKIs), remain underexplored in ESCC treatment.

Anlotinib is a multitargeted TKI that blocks VEGFR, PDGFR, FGFR and c-Kit with broad-spectrum antiangiogenic activities.^15^ Mechanistically, anlotinib potentially synergizes with ICIs by reprogramming the tumor microenvironment (TME) and downregulating PD-L1 expression in preclinical models.^16,17^ Additionally, as an antiangiogenic agent, anlotinib can enhance the delivery of chemotherapy drugs by inducing tumor vascular normalization.^18^ Therefore, anlotinib may be a promising combination partner with ICIs and chemotherapy. Benmelstobart, an IgG1 PD-L1 inhibitor, disrupts the interaction between PD-L1 with its receptors PD-1 and CD80, featuring sequence differences in complementarity-determining regions from other PD-L1 inhibitors.^19,20^ Benmelstobart includes a genetically-modified Fc domain designed to minimize FcγR binding, thus eliminating antibody-dependent cell-mediated cytotoxicity/complement-dependent cytotoxicity activities (ADCC/CDC).^19,20^ Preclinical and clinical data indicate that benmelstobart offers favorable safety compared to other PD-L1 blockades, with a reduced incidence of serious adverse events (AEs) (27.5% vs. 33.3%–54%).^21–24^ Previous studies have shown the encouraging activity of benmelstobart plus anlotinib without or with chemotherapy in treating various solid tumors.^25–27^ This evidence provides a rationale for combining anlotinib, chemotherapy, and PD-L1 inhibitors in advanced ESCC. We therefore initiated the first investigation of benmelstobart (PD-L1 inhibitor) anlotinib (multitargeted TKI), and chemotherapy in the first-line treatment of this disease.

## Results

### Patients characteristics

**Fig. 1:**
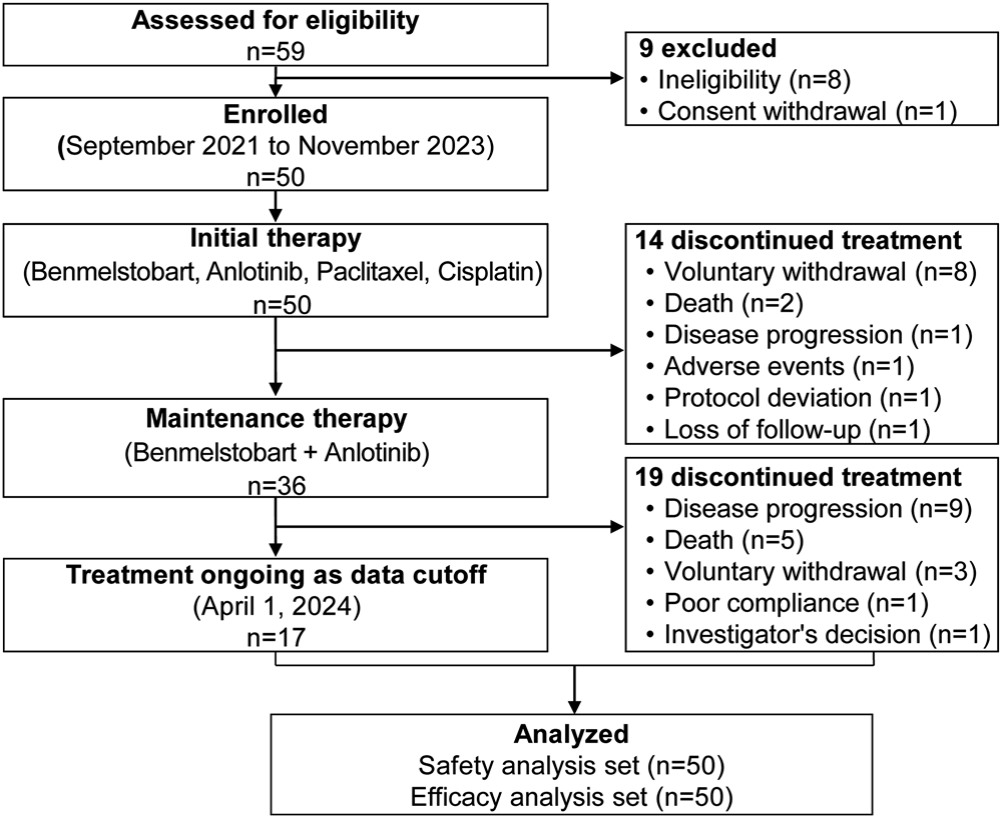
Patients disposition in the intention-to-treat population

Between September 2021 and November 2023, 50 eligible patients from 5 centers were enrolled and initiated the protocol-specified therapy (Fig. 1). Efficacy and safety were analyzed in all 50 as-treated patients. The median age was 64 years (range, 41–74), with 26 patients (52%) having a combined positive score (CPS) of PD-L1 below 1 (Table 1). Data cutoff occurred on April 1, 2024; the median follow-up period was 23.7 months (95% confidence interval [CI], 22.1–26.8).

**Table 1:** Baseline characteristics in 50 as-treated patients

	Patients (*n* = 50)
Age, years	64 (41–74)
<65years	24 (48)
≥65 years	26 (52)
Sex	
Male	39 (78)
Female	11 (22)
ECOG performance status	
0	12 (24)
1	38 (76)
Disease stage	
IVA	3 (6)
IVB	47 (94)
Number of metastatic sites	
1	11 (22)
2	32 (64)
≥2	7 (14)
Metastatic sites	
Lymph node	48 (96)
Lung	25 (50)
Liver	7 (14)
Prior surgery	17 (34)
Prior radiotherapy	8 (16)
Baseline PD-L1 CPS ^a^	
<1	26 (52)
≥1	12 (24)
<10	34 (68)
≥10	4 (8)
Unknown	12 (24)

Data were expressed as median (range) or no (%)

*ECOG* Eastern Cooperative Oncology Group, *PD-L1* programmed death ligand-1, *CPS* combined positive score

^a^ PD-L1 positivity was not required and PD-L1 status was assessed in 38 patients

### Treatment compliance and subsequent treatment

Among 50 as-treated patients, 14 (38%) discontinued initial treatment early (Fig. 1), mainly due to voluntary withdrawal (n = 8) and death (n = 2). The median initial therapy cycle was 5 (range, 1–6). During the initial phase, median relative dose-intensity were all 100% for benmelstobart (range, 33%–100%), anlotinib (range, 64%–100%), paclitaxel (range, 60%–100%), and cisplatin (range, 67%–100%). Mean treatment duration was 2.9 months (range, 0.03–4.2) for benmelstobart, 3.5 months (range, 0.03–4.8) for anlotinib, 3.0 months (range, 0.1–4.0) for paclitaxel, and 2.9 months (range, 0.03–3.9) for cisplatin. Thirty-six patients (72%) completed 4–6 cycles of initial therapy and proceeded to the maintenance phase. Maintenance treatment was discontinued in 19 patients (38%) because of disease progression (PD) (n = 9), death (n = 5), voluntary withdrawal (n = 3), poor compliance (n = 1), and investigator decision (n = 1). At cutoff date, 17 patients (34%) remained ongoing treatment.

After progression or withdrawal from the study treatment, 53.8% (7/13) of patients received subsequent treatments, with one patient (7.7%) undergoing third-line therapy. Among those who received subsequent treatments, the majority (6 [46.2%]) received anti-PD-1 therapy in later-line. Data on subsequent therapies are provided in supplementary Table S1.

### Efficacy

**Fig. 2:**
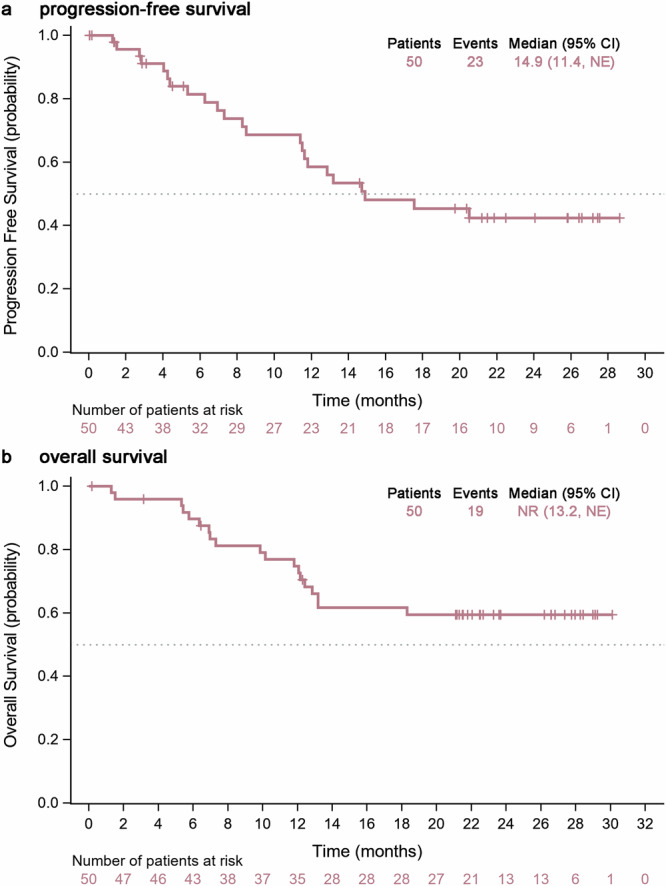
Survival outcomes. **a** Kaplan-Meier plots of progression-free survival per RECIST version 1.1 criteria. **b** overall survival. Vertical lines denote censored patients

Among 50 patients, 23 progression events or death occurred. The median progression-free survival (PFS) was 14.9 months (95% CI, 11.4-not estimable [NE]) according to Response Evaluation Criteria in Solid Tumors criteria version 1.1 (RECIST v1.1) and the estimated PFS rate at one year was 58.5% (95% CI, 41.9%–71.9%) (Fig. 2a). With 19 deaths, the median OS was not reached (NR; 95% CI, 13.2 months-NE); 1-year OS rate was estimated at 74.8% (95% CI, 59.8%–84.8%) (Fig. 2b).

**Fig. 3:**
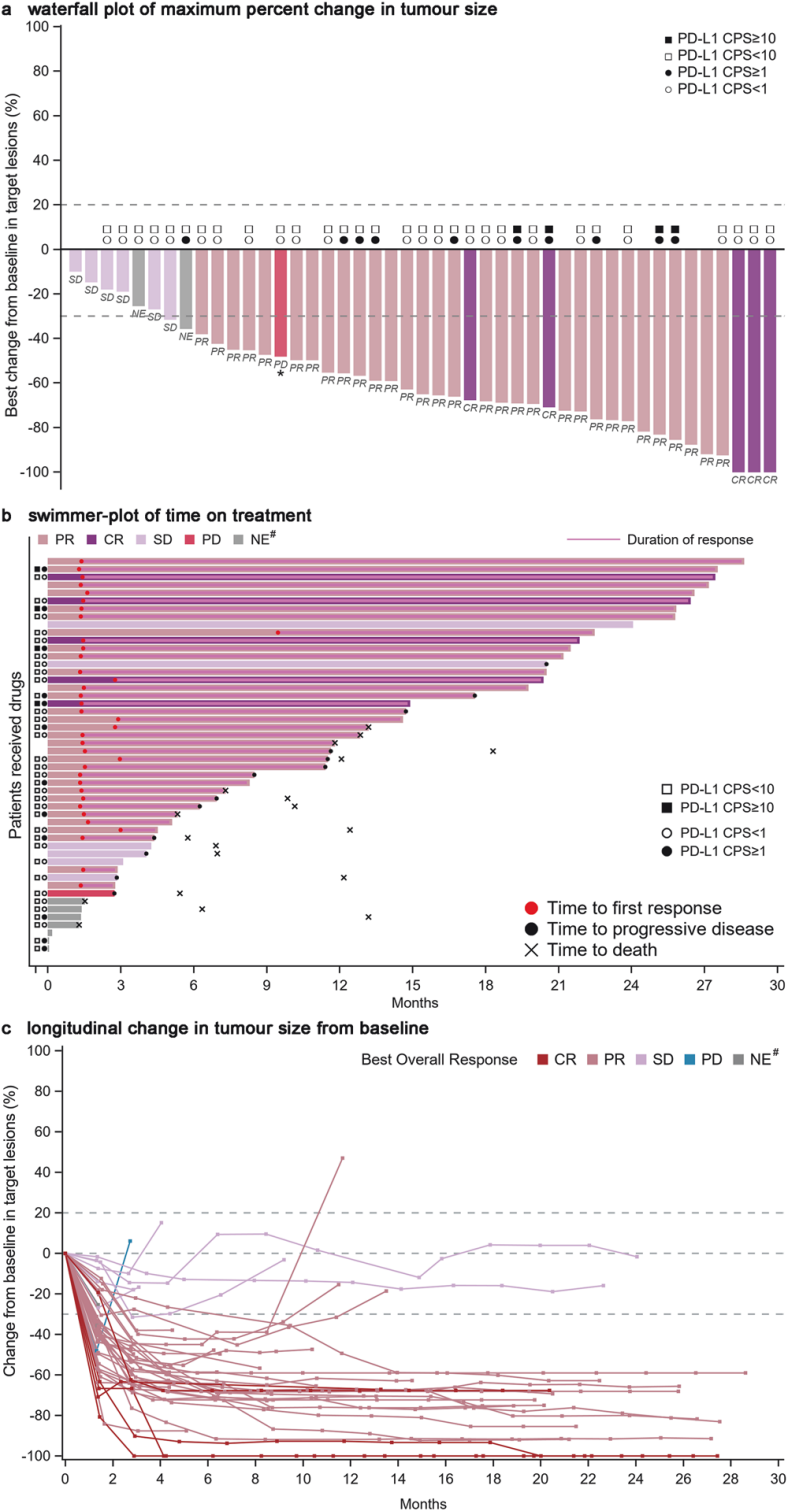
Tumor response. **a** Waterfall plot of maximum percent change in tumor size from baseline in each patient as measured by Response Evaluation Criteria in Solid Tumors (version 1.1). ^*^ Patients whose PD status was documented due to the appearance of new lesions. **b** Swimmer-plot of time on treatment. **c** Longitudinal change in tumor size from baseline. ^#^ NE indicates patients with unevaluable post-baseline assessment or no post-baseline assessment available for tumor response. CR complete response, PR partial response, PD progressive disease, SD stable disease, NE not evaluable

In the intention-to-treat (ITT) population, objective response was achieved in 36 patients (72.0%; 95% CI, 57.5%–83.8%), comprising 5 (10.0%) complete response (CR) and 31 (62.0%) partial response (PR) (Fig. 3a**;** Table 2). Target lesion size decreased from baseline in 45 (90.0%) of all patients (Fig. 3a). Of the 36 responders, durable responses lasting more than six months were observed in 30 patients (83.3%) (Fig. 3b), with a median duration of response (DoR) was 16.2 months (95% CI, 10.2–NE). Additional 6 patients (12.0%) were stable disease (SD), resulting in disease control in 42 (84.0%; 95% CI, 70.9%–92.8%) patients (Table 2). All six patients (100%) with SD showed initial tumor shrinkage from baseline (Fig. 3c). Among the 43 patients with post-baseline response assessments, the objective response rate (ORR) was 83.7% (95% CI, 69.3%–93.2%) and disease control rate (DCR) was 97.7% (95% CI, 87.7%–99.9%). Throughout the study, 13 patients experienced PD, with 12 (92.3%) developing oligo-progression (progression site 1–2) and 1 (7.7%) developing systemic progression (progression site ≥ 3).

**Table 2:** Tumor response to benmelstobart plus anlotinib and chemotherapy

	Patients (n = 50)
Best response	
Complete response	5 (10)
Partial response	31 (62)
Stable disease	6 (12)
Progressive disease	1 (2)
Not evaluable^a^	7 (14)
Objective response^b^	36 (72.0; 57.5–83.8)
Disease control^c^	42 (84.0; 70.9–92.8)

Data were expressed as n (%) or n (%; 95% confidence interval)

^a^ Not evaluable indicates patients with unevaluable post-baseline assessment or no post-baseline assessment available for tumor response

^b^ Objective response=Complete response plus partial response

^c^ Disease control=Complete response, partial response, plus stable disease

PD-L1 CPS were available for 38 patients. Exploratory analyses based on PD-L1 CPS status indicated an ORR of 100% (4/4) for patients with CPS ≥ 10 and 67.6% (23/34) for those with CPS < 10; 75.0% (9/12) for CPS ≥ 1 and 69.2% (18/26) for CPS < 1 (Fig. 3a). Median PFS was not associated with PD-L1 CPS, irrespective of cutoff values (cutoff ≥ 1, 12.9 vs 14.9 months; ≥ 10, 12.9 months vs. NR) (supplementary Fig. S1). The median OS was NR in either subgroup, regardless of PD-L1 CPS (supplementary Fig. S2). Subgroup analyses of efficacy results (PFS or OS) based on other baseline characteristics (e.g., sex, age, metastasis, Eastern Cooperative Oncology Group [ECOG] performance status, prior surgery, or radiotherapy) are also available in supplementary Figs. S1 and S2. Patients without liver metastases exhibited significantly longer PFS (20.5 vs. 4.7 months; *p* < 0.0001) and OS (NR vs. 6.3 months; *p* < 0.0001) compared to those with liver metastases, while other characteristics had no correlations with PFS or OS (supplementary Figs. S1 and S2). Nevertheless, the significance based on liver metastases should be considered preliminary due to the limited number of cases.

### Safety

Forty-six patients (92%) reported any grade AEs and 39 (78%) reported grade ≥3 events (Table 3). Common AEs included leukopenia (32 patients [64%]; of whom 12 [24%] with grade ≥3), neutropenia (29 [58%]; 22 [44%] with grade ≥3), anemia (28 [56%]; 3 [6%] with grade ≥3), hypertension (26 [52%]; 10 [20%] with grade ≥3), nausea (26 [52%]; none with grade ≥3), and anorexia (25 [50%]; 4 [8%] with grade ≥3).

**Table 3:** Adverse events and treatment-related adverse events^a^

	Adverse events (n = 50)		Treatment-related adverse events (n = 50)	
	Any grade	Grade 3 or worse	Any grade	Grade 3 or worse
Any events	46 (92)	39 (78)	46 (92)	37 (74)
Events leading to discontinuation	3 (6)	-	-	-
Events leading to dose reduction	17 (34)	-	-	-
Immune-related events	24 (48)	3 (6)	-	-
Frequent events (≥20%)				
Leukopenia	32 (64)	12 (24)	31 (62)	12 (24)
Neutropenia	29 (58)	22 (44)	27 (54)	22 (44)
Anemia	28 (56)	3 (6)	28 (56)	3 (6)
Hypertension	26 (52)	10 (20)	26 (52)	10 (20)
Nausea	26 (52)	0	26 (52)	0
Anorexia	25 (50)	4 (8)	25 (50)	4 (8)
Thrombocytopenia	24 (48)	2 (4)	23 (46)	2 (4)
Fatigue	22 (44)	0	19 (38)	0
Lymphopenia	14 (28)	2 (4)	12 (24)	2 (4)
Diarrhea	13 (26)	2 (4)	9 (18)	2 (4)
Vomiting	12 (24)	0	12 (24)	0
Cough	11 (22)	2 (4)	4 (8)	0
Weight loss	11 (22)	2 (4)	8 (16)	1 (2)
Constipation	11 (22)	0	6 (12)	0
Hypothyroidism	10 (20)	0	9 (18)	0
Alopecia	10 (20)	0	10 (20)	0

Data were expressed as n (%). The safety set included all patients who received at least one dose of study treatment

^a^ Adverse events were classified according to Common Terminology Criteria for Adverse Events, version 5.0; Adverse events or treatment-related adverse events occurring in 20% of patients are listed

Regarding treatment-related AEs (TRAEs), 46 patients (92%) experienced any grade events and 37 (74%) were grade 3 or worse (Table 3). Frequent TRAEs were leukopenia (31 patients [62%]; of whom 12 [24%] with grade ≥3), anemia (28 [56%]; 3 [6%] with grade ≥3), neutropenia (27 [54%]; 22 [44%] with grade ≥3), hypertension (26 [52%]; 10 [20%] with grade ≥3), nausea (26 [52%]; none with grade ≥3), and anorexia (25 [50%]; 4 [8%] with grade ≥3).

Immune-related AEs (irAEs) occurred in 24 patients (48%) with only 3 (6%) experiencing grade ≥3 irAEs (supplementary Table S2). The most common irAEs in at least 10% of patients were hypothyroidism (9 patients [18%]), diarrhea (7 [14%]), and alanine aminotransferase increased (5 [10%]). Seventeen patients (34%) required dose reduction of anlotinib or chemotherapy due to AEs, and 3 (6%) discontinued treatment secondary to toxicity.

During the treatment, three bleeding events (6%) were reported, including one grade 5 intracranial hemorrhage, one grade 1 epistaxis, and one grade 1 lower gastrointestinal hemorrhage, which was possibly attributed to anlotinib. The gastrointestinal hemorrhage did not lead to dose reduction or discontinuation of anlotinib and was managed successfully with supportive care. Two patients (4%) experienced grade ≥3 esophageal fistula; however, one was deemed unrelated to treatment. Another grade 5 esophageal fistula occurred in a patient who had previously undergone extensive radiotherapy (5400 cGy in 30 fractions) for a primary lesion in the cervical and upper thoracic segment of the esophagus and was considered possibly related to treatment according to the investigator’s assessment. Nineteen deaths (38%) occurred on treatment or during follow-up. Two deaths (4%) were attributed to non-disease-related causes (COVID-19) and 13 (26%) were attributed to PD. Four deaths (8%) occurred as a result of AEs, including one each with myelosuppression, pulmonary infection, intracranial hemorrhage, and esophageal fistula.

## Discussion

This phase II study offers the first prospective evaluation of a first-line combination therapy involving PD-L1 inhibitor, multitargeted TKI, and chemotherapy for advanced or metastatic/recurrent ESCC. This combination achieved median PFS of 14.9 months, meeting the primary endpoint. Additionally, our combination yielded a promising ORR of 72.0%. The benefits observed regarding PFS, OS, and ORR were consistently encouraging, regardless of PD-L1 expression. Furthermore, this combination had a generally tolerable safety profile without any novel safety signals.

Current first-line immunochemotherapy options provide limited therapeutic benefits in advanced ESCC, with ORR ranging from 47% to 70%.^5–8,28^ Encouragingly, our ORR data (72.0%) is at the upper end of this spectrum and is comparable to that of another regimen involving immunochemotherapy and antiangiogenic apatinib, which reported an ORR of 80%.^29^ Equally noteworthy is that the durable and deep response was observed in a substantial proportion of responders (83.3%), with median DoR reaching up to 16.2 months, which was favorably compared with median values (about 6–10 months) for other immunochemotherapy with,^29^ or without anti-angiogenesis regimens.^5–7^

Building upon the modest benefits of immunotherapy-based regimes with median PFS of 5.7–7.2 months (1-year PFS, ~25%) and OS of 12.6–17.0 months (1-year OS, 50%–65%),^5–8,28^ our regimen reported an encouraging PFS of 14.9 months (1-year PFS, 58.5%). In ALTER-E002,^30^ the effects of anlotinib plus chemotherapy on PFS prolongation were limited (median, 8.4 months; 1-year PFS, 25%) despite increased ORR, whereas here adding benmelstobart prolonged PFS dramatically. We speculate that this enhanced activity may be owing to the synergy of anlotinib with ICIs and chemotherapy.^17,31–35^ Advanced ESCC with low PD-L1 expression usually derive limited PFS benefit (<8 months) from immunochemotherapy.^5–8,29^ Interestingly, our regimen produced promising outcomes in both PFS (positive vs. negative: 12.9 vs. 14.9 months) and ORR (75% vs. 69.2%) irrespective of PD-L1 status. Long-term efficacy was also promising, with median OS of NR and 1-year OS of 74.8% at 23.7 months of follow-up. The OS curve began to plateau after 18 months with a long tail until 30 months, suggesting the potential for durable survival. Limitations of cross-trial comparison should be acknowledged. For instance, our study cohort included fewer patients with liver metastases (14% vs 18–24%) compared to similar studies, which might introduce a bias in favor of our results. Overall, satisfying benefits from our regimen supported their potential utility in this advanced disease irrespective of PD-L1 status, thereby enhancing patient access to immunotherapy-based therapy.

This combination demonstrated an acceptable tolerability profile. Despite 78% of patients reporting grade ≥3 AEs, most frequent events were possibly chemotherapy-related. Similar to previous reports on antiangiogenic therapies,^36^ we observed frequent occurrences of hypertension (a known AE of anlotinib),^37^ though it was manageable with supportive care. Safety data were generally consistent with that of other cancer types.^27^ The occurrence of potential irAEs (48%) and TRAE-related discontinuation (6%) from our regimen seems unfrequent compared with camrelizumab plus chemotherapy (84.6%; 12.1%).^28^ Only 6% of patients reported grade ≥3 irAEs, similar to that seen with other regimens (7%–10%).^7,28^ A meta-analysis reported that PD-L1 inhibitors have fewer grade ≥3 toxic effects than PD-1 inhibitors.^38^ As expected, grade ≥3 TRAEs (74%) of our regimen was numerically lower than that of PD-1 inhibitors-based immunochemotherapy combined with TKIs (90.0%),^29^ comparable to those without TKIs (63.4%–72%),^6,28^ implying its preferable safety profile, although considering the possibility that patient background, chemotherapy regimen, or treatment duration affect toxicity. From mechanistic aspects, unlike PD-1 inhibitors, benmelstobart targets PD-L1 but not influences PD-1/PD-L2 interactions, thereby reducing the risk of immune-related toxicity.^39^ Moreover, the preferable safety may be explained by the fact that benmelstobart is an Fc-engineered humanized IgG1 antibody, which reduced FcγR binding and eliminated ADCC/CDC activities, preventing non-specific immune cell killing.^21,40^ This favorable safety profile could potentially improve patients’ quality of life and treatment compliance.

This exploratory study was limited by non-randomized study design, lacking the comparison with standard-of-care or other existing regimens. Besides, 28% of patients discontinued initial therapy might be considered as a limitation; however, most were attributed to lost follow-up or voluntary withdrawal (18%), particularly influenced by the COVID-19 outbreak from October 2021 to November 2022. Nevertheless, efficacy was analyzed in the ITT population to minimize the impact of these dropouts. Lacking full assessment of exploratory biomarkers (except for PD-L1 expression) was another limitation, underscoring the need to identify additional predictive biomarkers.

In conclusion, benmelstobart plus anlotinib and chemotherapy demonstrated impressive survival and durable responses in advanced or metastatic/recurrent ESCC, irrespective of PD-L1 status. Furthermore, this regimen exhibited a potentially preferable safety profile. Based on these results, this regimen may serve as an effective and safe therapeutic option for this patient population and warrants further investigation in confirmatory randomized studies.

## Materials and methods

### Ethics approval

The study was performed in line with the Good Clinical Practice Guideline and Declaration of Helsinki. Informed consent was obtained from each subject. This study was approved by ethics committees of participant centers and registered in ClinicalTrials.gov (NCT05013697).

### Study design and patients

This study was a multicohort, multi-center, phase II study and enrolled patients with advanced or metastatic/recurrent ESCC at five sites in China. The study investigated benmelstobart and chemotherapy with anlotinib in cohort 1 or benmelstobart and chemotherapy without anlotinib in cohort 2. Cohort 2 continued enrollment up to approximately 30 patients after the completion of cohort 1 enrollment, as defined in the protocol, and the results will be reported once fully accrued. Here, the results of cohort 1 were reported.

Patients aged 18–75 with histologically confirmed, unresectable, recurrent/metastatic ESCC were eligible. Patients were required to have not received previous systemic therapy or have tumor recurrence more than six months after completing (neo) adjuvant or radical therapy. Key inclusion criteria were measurable lesions, an expected survival of ≥3 months and ECOG performance status of 0–1. Patients with esophageal stent placement, ulcerative disease, interstitial lung disease, substantial malnutrition, autoimmune disease, symptomatic brain metastasis, carcinomatous meningitis, or tumor invasion into adjacent organs of the lesion were excluded. We also excluded patients who did not receive surgical resection of the primary lesion but had no tumor regression after radiotherapy or had recurrence or metastasis within one year after adjuvant chemotherapy with paclitaxel. Other exclusion criteria included severe and/or uncontrolled systemic disease, allergic reactions to study drugs, other malignancies, or women who were pregnant or breastfeeding. Full eligibility criteria are listed in supplementary materials.

### Procedures and assessments

Patients in cohort 1 received 4 to 6 cycles (every three weeks) of initial therapy, including benmelstobart (1200 mg intravenously [i.v.]; day 1) plus anlotinib (10 mg orally; days 1–14) combined with paclitaxel (135 mg/m^2^ i.v.; day 1) and cisplatin (60–75 mg/m^2^ i.v.; divided into days 1–3). Patients with response or SD maintained with benmelstobart plus anlotinib at the same dose until confirmed PD or unacceptable AEs. Benmelstobart dose could be delayed for a maximum of 12 weeks and not permitted for reduction. Dose delays or reductions of anlotinib were allowed for toxicities under dose reduction criteria prespecified in the protocol. If the patients remain intolerant after the dose adjustment of anlotinib, treatment was terminated. The maximum duration per episode of dose delays was two weeks, and up to twice delays per cycle were allowed; otherwise, study treatment was discontinued, but tumor evaluation continued.

Radiological assessment was conducted by investigators at baseline, every 2 cycles during initial therapy, and thereafter every 3 cycles during maintenance therapy, as per RECIST v1.1. Responses were reconfirmed at least six weeks later if meeting response criteria. AEs were monitored by evaluating ECOG performance status, vital signs, and laboratory parameters until 30 days after the final dose of treatment, and their severity was graded by the National Cancer Institute Common Terminology Criteria for Adverse Events (version 5.0). Baseline tumor biopsies were collected for immunohistochemistry analysis of PD-L1 expression using VENTANA PD-L1 SP263 assay. The PD-L1 positivity was defined according to the CPS cutoff (CPS = [number of PD-L1-positive tumor or immune cells]/[number of viable cells]×100).

### Outcomes

The primary endpoint was PFS assessed according to RECIST v1.1 (the date from enrollment until PD or death). Secondary endpoints included ORR (patients with CR or PR), DCR (patients with objective response or SD), DoR (the date from response until PD or death), OS (the date from enrollment until death), and safety. Exploratory endpoint was prespecified as the association of PD-L1 status with efficacy outcomes.

### Statistical analysis

Based on a historical control of 5.7 months with immunochemotherapy regimens in patients with advanced ESCC, we hypothesized an expected median PFS of 9.8 months with benmelstobart plus chemotherapy with anlotinib. With 12 months of enrollment and 12 months of follow-up, approximately 27 expected events in 38 patients would provide a power of 80% at a two-sided α of 0.05 to demonstrate superior efficacy of this combination than the historical control. Considering a dropout rate of 20%, we planned to enroll 48 patients.

Efficacy and safety were evaluated in patients who received ≥1 dose of study regimen. Characteristics and safety data were descriptively summarized. Time-to-event endpoints were analyzed using Kaplan-Meier method. Patients without endpoint events were censored at the last assessment date. The 95% CIs of response data were estimated with the Clopper-Pearson method. Prespecified subgroups for survival were estimated according to baseline characteristics. Statistical analyses were performed using SAS v.9.4.

## Supplementary information

Supplemental Material

Protocol

## Data Availability

Datasets analyzed in this study are available upon reasonable request to the corresponding author.
