# Supplementary material for: First-line benmelstobart plus anlotinib and chemotherapy in advanced or metastatic/recurrent esophageal squamous cell carcinoma: a multi-center phase 2 study
Source: Signal Transduct Target Ther. 2024 Nov 8;9:303. doi: 10.1038/s41392-024-02008-7 (PMC11544088; doi:10.1038/s41392-024-02008-7)
Supplement: Supplementary file 1 — Supplemental Material [file 41392_2024_2008_MOESM1_ESM.docx]

Supplementary Materials for

First-line benmelstobart plus anlotinib and chemotherapy in advanced or metastatic/recurrent esophageal squamous cell carcinoma: a multi-center phase 2 study

Ning Li^#^, Jin Xia^#^, Xiaohui Gao^#^, Jianwei Zhou, Yonggui Hong, Donghai Cui, Xuesong Zhao, Tao Wu, Yanzhen Guo^*^, Junsheng Wang^*^, Suxia Luo^*^

^*^Correspondence to: guoyanzhen177@126.com; 1207741934@qq.com; luosxrm@163.com

**This PDF file includes:**

Materials and Methods

Figures. S1 to S2

Tables S1 to S2

Materials and Methods

Study population

1. **Inclusion criteria**

Subjects can participate in the study only if all the following criteria are met:

1. Histopathologically confirmed, advanced, recurrent, or metastatic esophageal squamous cell carcinoma (ESCC) (excluding mixed adenosquamous carcinoma);
2. No prior systemic therapy or have tumour recurrence more than 6 months after the completion of (neo) adjuvant or radical therapy (including radical surgery and radical chemoradiotherapy);

Note: Patients with advanced or recurrent non-target lesions who progressed again after radiotherapy alone were included. The time from the end of palliative treatment for local lesions (non-target lesions) to enrollment was more than 2 weeks.

1. At least one measurable lesion according to Response Evaluation Criteria in Solid Tumors version 1.1 (RECIST v1.1); Measurable lesions should not have received local treatment such as radiotherapy (lesions located in the area of previous radiotherapy can also be selected as target lesions if they are confirmed to have progressed and meet RECIST v1.1 criteria);
2. Age 18-75 years;
3. Eastern Cooperative Oncology Group-Performance status (ECOG-PS) of 0-1; Predicted life expectancy of ≥3 months;
4. Adequate function of the important organs as evidenced by the following:
5. Hemanalysis:

- hemoglobin (Hb) ≥90g/L (no blood transfusion within 28 days);
- absolute neutrophil count (ANC) ≥1.5×10^9^/L;
- platelets (PLT) ≥100×10^9^/L.

1. Biochemistry:

- total bilirubin (TBIL) ≤1.5×upper limit of normal (ULN)；
- alanine aminotransferase (ALT) and aspartate aminotransferase (AST) ≤2.5×ULN; ALT and AST ≤5×ULN in patients with liver metastases;
- Creatinine (Cr) ≤1.5×ULN and creatinine clearance rate (CCr) ≥60 mL/min (Cockcroft-Gault formula).

1. Adequate coagulation function: international normalization ratio (INR), or prothrombin time (PT) ≤1.5×ULN;
2. Women of reproductive age were required to use appropriate contraception from the time of screening until 3 months after discontinuation of study treatment and were not breast-feeding. A negative pregnancy test or one of the following criteria before the initiation of dosing proved that there was no risk of pregnancy:

a. Postmenopausal status was defined as age ≥50 years and amenorrhea for at least 12 months after discontinuation of all exogenous hormone replacement therapy;

b. Women aged <50 years were also considered postmenopausal if they had amenorrhea for 12 months or more after discontinuation of all exogenous hormone therapy and the luteinizing hormone (LH) and follicle-stimulating hormone (FSH) were accorded with the laboratory normal reference range;

c. Patients who had undergone irreversible sterilization procedures, including hysterectomy, bilateral oophorectomy, or bilateral salpingectomy, with the exception of those who had bilateral tubal ligation.

For men, consent is given to use an appropriate method of contraception or to have been surgically sterilized during the trial period and 8 weeks after the last drug administration.

1. Be willing and able to provide written informed consent for the trial, and have better compliance with follow-up.
2. **Exclusion criteria**
3. Patients with ESCC who have complete obstruction under endoscopic guidance and need interventional therapy to relieve obstruction;
4. Patients with ulcerative ESCC;

Note: This refers primarily to patients with ulcers adjacent to blood vessels that increase the risk of bleeding.

1. Patients after esophageal or tracheal stent placement;
2. Patients with a high risk of bleeding or perforation due to tumor invasion of adjacent organs (large arteries or trachea) of the esophageal lesion, or with established fistulas;
3. Patients who had hematemesis, bloody stool and daily blood loss ≥2.5 mL or any CTCAE grade ≥3 bleeding events within 3 months before screening, or who had any evidence of bleeding, regardless of severity, or whose history was judged by the investigator to be ineligible for enrollment;
4. Patients who have allergic reactions to drug formulations or excipient components or similar drugs;
5. Patients who had received adjuvant chemotherapy with paclitaxel and had recurrence or metastasis within one year;

Note: Patients with recurrence or metastasis for more than one year could be included in the study.

1. Factors significantly affecting oral medication (e.g. swallowing difficulty, chronic diarrhea, and intestinal obstruction);
2. The liver metastatic burden with accounting for approximately more than 50% of the total liver volume;
3. Patients with any severe and/or uncontrolled illness, including:

- Patients with poor blood pressure control using antihypertensive drugs (systolic blood pressure ≥150 mmHg or diastolic blood pressure ≥100 mmHg); patients with grade II or above myocardial ischemia or myocardial infarction, arrhythmia (including QT interval ≥480ms); patients with Grade III-IV cardiac insufficiency, or with left ventricular ejection fraction (LVEF) <50% via the cardiac color ultrasound;
- Active or uncontrolled severe infection;
- Liver diseases such as cirrhosis, decompensated liver disease, chronic active hepatitis;
- Poor diabetes control (fasting blood glucose [FBG] >10 mmol/L);
- Urinary protein ≥ ++, and confirmed 24-hour urinary protein >1.0 g;

1. Unhealed wound or fracture for a long time;
2. Patients with ESCC who have active bleeding of the primary lesion within 2 months; NCI CTCAE grade >1 pulmonary hemorrhage within 4 weeks prior to enrollment; NCI CTCAE grade >2 other site bleeding within 4 weeks prior to enrollment; patients with bleeding tendencies (e.g., active gastrointestinal ulcers) or those receiving thrombolytic or anticoagulation therapy such as warfarin, heparin, or similar agents;
3. Patients who have undergone major surgical procedures (e.g., craniotomy, thoracotomy, or laparotomy) within 4 weeks before the first study dose or are anticipated to require major surgery during the study treatment;
4. Patients with a history of gastrointestinal perforation and/or fistula within 6 months prior to enrollment, or with thromboembolic events such as cerebrovascular accidents (including transient ischemic attacks), deep vein thrombosis, and pulmonary embolism;
5. Known presence of symptomatic central nervous system metastases and/or carcinomatous meningitis;
6. Clinically significant ascites, including ascites detectable on physical examination, ascites that have been treated previously or currently require treatment, and minimal ascites evident by imaging only but without symptoms;
7. Patients with moderate bilateral pleural effusion, or significant pleural effusion on one side, or those who have developed respiratory impairment requiring drainage;
8. Known active pulmonary tuberculosis;
9. Interstitial lung disease requiring steroid hormone therapy;
10. Uncontrolled metabolic disturbances or other non-malignant or systemic disease or secondary reaction to cancer, which can lead to higher medical risks and/or uncertainty of survival evaluation;
11. Patients with significant malnutrition;
12. Patients with a history of psychotropic substance abuse and difficulty achieving abstinence or with psychiatric disorders;
13. Patients with a history of immunodeficiency, including those with a positive HIV test or suffering from other acquired or congenital immunodeficiency diseases, or those with a history of organ transplantation;
14. History of other primary malignant tumors, except for the following: 1) Complete remission of malignant tumors for at least 2 years prior to enrollment, without further treatment during the study; 2) Adequately treated non-melanoma skin cancer or lentigo maligna with no evidence of disease recurrence; 3) Adequately treated carcinoma in situ with no evidence of disease recurrence;
15. Pregnant or lactating women;
16. > Grade 1 unresolved toxicity according CTCAE due to any previous treatment, excluding alopecia;
17. Patients who have received first-line chemotherapy for advanced disease or radiotherapy prior to first dose;

Note: Patients who previously received local radiotherapy can be eligible if: the end of radiotherapy is more than 3 weeks from the start of study treatment; the target lesion selected for this study is not within the radiation field; or the target lesion is located within the radiation field, but progression has been confirmed; without chemotherapy, immunotherapy and targeted therapy during radiotherapy.

1. Received treatment with Chinese patent medicines with anti-tumor indications as specified in the approved NMP-approved drug instructions (including Fufangbanmao Capsules, Kangai Injection, Kanglaite Capsules / Injection, Aidi Injection, Brucea javanica oil Injection/Capsules, Xiaoaiping Tablets/Injection, Huachansu Capsules, etc.) within 2 weeks prior to the first administration of the medication.
2. Patients previously treated with VEGFR small molecule inhibitors, such as anlotinib, apatinib, lenvatinib, sorafenib, sunitinib, regorafenib and furazolidone;
3. Patients previously received treatment with anti-PD-1 or anti-PD-L1/PD-L2 inhibitors or other therapies acting on T cell costimulatory targets or checkpoints;
4. History of live attenuated vaccination 28 days prior to first dose or planned live attenuated vaccination during the study;
5. Active autoimmune disease requiring systemic therapy (eg, disease-modifying drugs, corticosteroids, or immunosuppressants) within 2 years prior to first dose. Replacement therapy (e.g., thyroxine, insulin, or physiological corticosteroids for adrenal or pituitary insufficiency, etc.) is not considered as systemic therapy;
6. Diagnosis of immunodeficiency or ongoing systemic glucocorticoid therapy or any other form of immunosuppressive therapy (>10 mg/ Prednisone or other effective hormone), and continued use within 2 weeks prior to the start of study treatment;
7. Participated in other clinical trials of anti-tumor drugs within 4 weeks prior to first dose (the washout period is calculated from the end time of the last treatment);
8. Patients who have concomitant diseases that seriously jeopardize the patient's safety or affect the patient's completion of the study, or are considered unsuitable for enrollment for other reasons according to the judgment of the investigator.
9. **Criteria for participant withdrawals**
10. The patients demonstrate poor compliance, regularly failing to adhere to the prescribed medication regimen (patients do not take medication at the planned time and dose more than two times for no reason, and the investigator's judgment will seriously affect the following study and study results);
11. Treatment with other systemic antineoplastic agents (e.g., chemotherapy, hormone therapy, targeted therapy, or biologic agents) that affect the judgment of efficacy;
12. Patients who experienced serious adverse events (SAEs) and were not suitable for further study according to the investigator’s judgment or who had an unintended pregnancy;
13. Patients who are unwilling to continue the clinical trial and insist on withdrawing;
14. The investigators deemed it necessary to stop the study.
15. **Criteria of participant removal**
16. Patients with chemotherapy, surgery, or other study drugs beyond the protocol during the trial;
17. Patients who failed to meet the inclusion criteria were mistakenly included;
18. Patients who did not receive treatment;

Note: Patients who met criteria 1 to 2 were included in the safety analysis.

1. **Criteria of termination**
2. Patients with disease progression;
3. If serious adverse events occurred, treatment was discontinued based on the decisions of investigators.


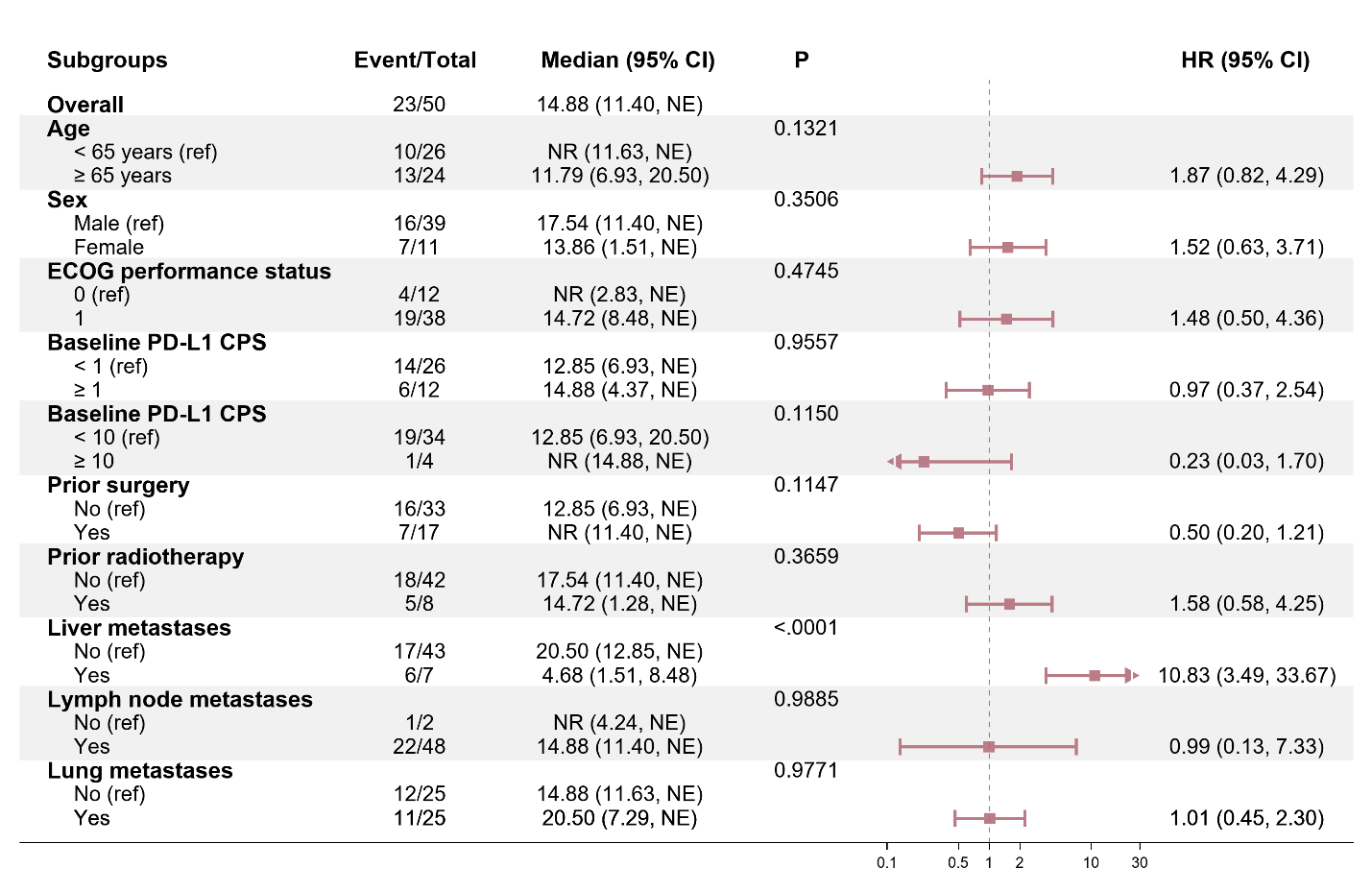


Figure. S1. Progression-free survival by patient subgroups

ECOG Eastern Cooperative Oncology Group, CI confidence interval, NR not reached, NE not estimable, PD-L1 [programmed death-ligand 1](https://zhuanlan.zhihu.com/p/116480937), CPS combined positive score


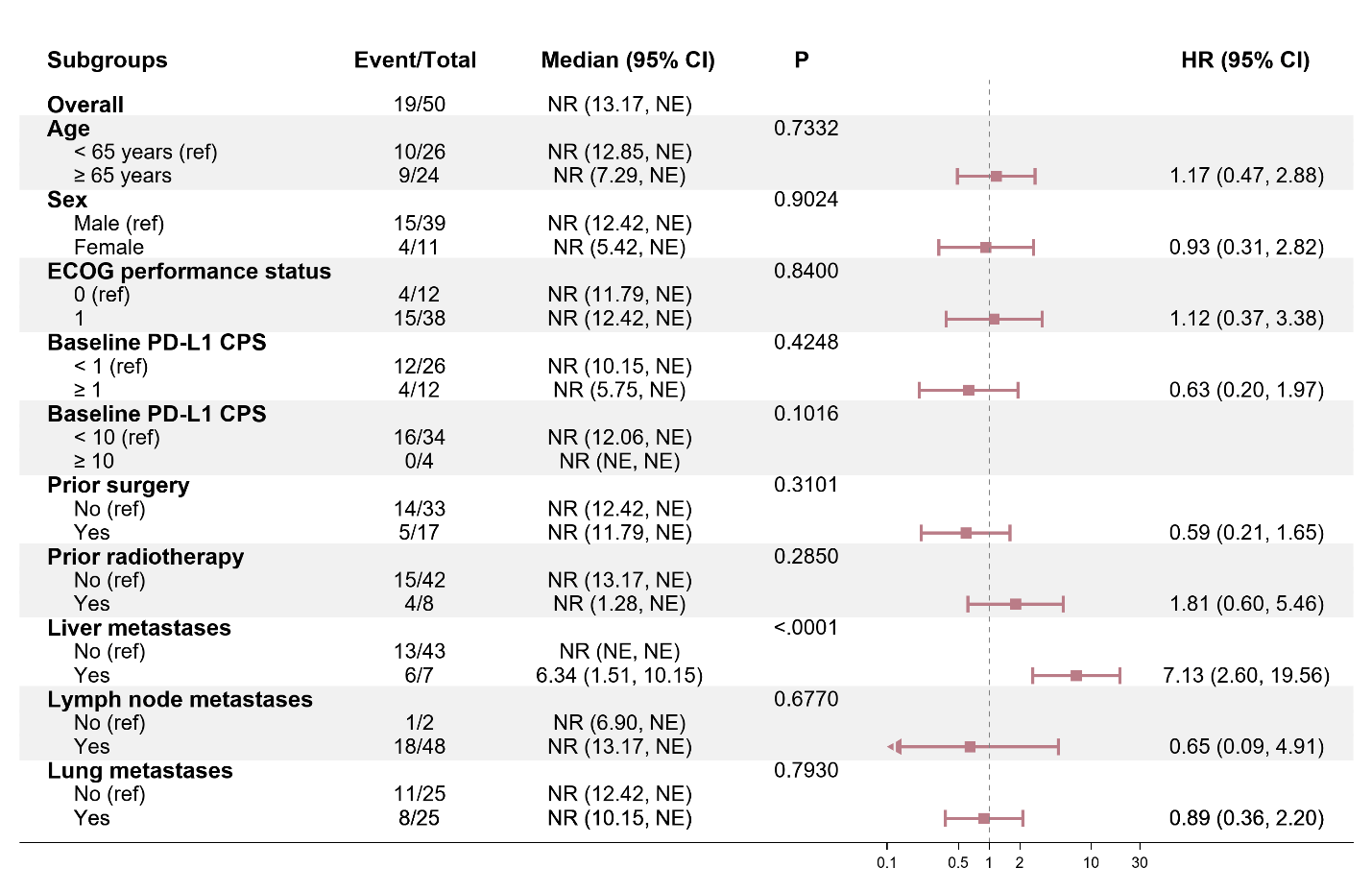


Figure. S2. Overall survival by patient subgroups

ECOG Eastern Cooperative Oncology Group, CI confidence interval, NR not reached, NE not estimable, PD-L1 programmed death-ligand 1, CPS combined positive score

Table S1. Subsequent treatment of 13 patients after progression or withdrawal from study treatment

| **Subsequent regimens n (%)** | **2^nd^** | **3^rd^** |
| --- | --- | --- |
| Irinotecan+Carboplatin | 1 (7.7) | 0 |
| Camrelizumab | 1 (7.7) | 0 |
| Camrelizumab+Nimotuzumab | 2 (15.4) | 0 |
| Penpulimab+Paclitaxel+Cisplatin | 1 (7.7) | 0 |
| Apatinib+S-1 | 1 (7.7) | 0 |
| Camrelizumab+Apatinib+Nedaplatin+5-FU | 0 | 1 (7.7) |
| TCM+Tislelizumab+Nab-Paclitaxel | 1 (7.7) | 0 |
| **Total (%)** | **7 (53.8)** | **1 (7.7)** |

*2^nd^* second-line, *3^rd^* third-line, *5-FU* Fluorouracil, *TCM* traditional Chinese medicine.

Table S2. Immune-related adverse events*

|  | **Any grade** | **Grade 3 or worse** |
| --- | --- | --- |
| Any events | 24 (48) | 3 (6) |
| Hypothyroidism | 9 (18) | 0 |
| Diarrhea | 7 (14) | 1 (2) |
| Alanine aminotransferase increased | 5 (10) | 1 (2) |
| Aspartate aminotransferase increased | 4 (8) | 1 (2) |
| Hyperthyroidism | 4 (8) | 0 |
| Blood bilirubin increased | 3 (6) | 0 |
| Liver dysfunction | 2 (4) | 1 (2) |
| Thyroid stimulating hormone increased | 2 (4) | 0 |
| Unconjugated bilirubin increased | 2 (4) | 0 |
| Blood creatinine increased | 2 (4) | 0 |
| Gamma-glutamyl transferase increased | 1 (2) | 0 |
| Infectious pneumonia | 1 (2) | 0 |
| Thyroid hormone decreased | 1 (2) | 0 |
| Conjugated bilirubin increased | 1 (2) | 0 |
| Rash | 1 (2) | 0 |
| Rash maculo-papular | 1 (2) | 0 |
| Triiodothyronine decreased | 1 (2) | 0 |
| Supraventricular extrasystole | 1 (2) | 0 |
| Free thyroid hormone decreased | 1 (2) | 0 |
| Free triiodothyronine decreased | 1 (2) | 0 |
| Rash acneiform | 1 (2) | 0 |

* Immune-related adverse events were identified using MedDRA preferred terms and graded according to Common Terminology Criteria for Adverse Events, version 5.0; All immune-related adverse events are listed.
