# Supplementary material for: First-line benmelstobart plus anlotinib and chemotherapy in advanced or metastatic/recurrent esophageal squamous cell carcinoma: a multi-center phase 2 study
Source: Signal Transduct Target Ther. 2024 Nov 8;9:303. doi: 10.1038/s41392-024-02008-7 (PMC11544088; doi:10.1038/s41392-024-02008-7)
Supplement: Supplementary file 2 — Protocol [file 41392_2024_2008_MOESM2_ESM.docx]

Paclitaxel + cisplatin + TQB2450 with or without anlotinib in first-line treatment of advanced esophageal squamous cell carcinoma: a multicenter exploratory study

| Party code: | TQB2450-II-13 |
| --- | --- |
| Version No.: | 1.2 |
| Version Date: | August 01, 2021 |

| Leading site: | Henan Cancer Hospital |
| --- | --- |
| Principal Investigator: | Suxia Luo |
|  |  |
|  |  |

Protocol Signature Page (Leading site)

I will earnestly perform my duties as an investigator and personally participate in or directly guide this clinical study in accordance with current GCP regulations. I have read and confirmed this protocol (Protocol No.: TQB2450-II-13; Version No.: 1.2; version date: August 01, 2021, and agree with the scientificity and ethicality of this protocol. We will perform our duties in accordance with the laws of China, the Declaration of Helsinki, Chinese GCP, and the provisions of this study protocol, As the leading site, we will actively coordinate all participating sites to carry out this clinical study in accordance with the protocol, Modifications to the protocol will be made only after notification to the sponsor and approval will be required from the Ethics Committee before implementation, unless measures are necessary to protect the safety, rights, and interests of the subjects.

We will keep this study protocol confidential.

Leading site: Henan Cancer Hospital

|  |  |
| --- | --- |
| Principal Investigator (Signature) | Date of Signature (DD/MMM/YYYY) |

Protocol Signature Page (Study site)

I will earnestly perform my duties as an investigator and personally participate in or directly guide this clinical study in accordance withcurrent GCP regulations. We have read and confirmed this protocol (protocol No. TQB2450-II-13; version No.: 1.2, version date: August 01, 2021, and the scientificity and ethicality of this protocol are approved. We will perform relevant duties in accordance with the laws of China, the Declaration of Helsinki, China's GCP, and the stipulations of this study protocol, and only modify the protocol after notifying the sponsor, which can be implemented only with the approval of the Ethics Committee, unless measures must be taken to protect the safety, rights, and interests of the subjects.

We will keep this study protocol confidential.

| Study sites: |
| --- |
|  |

|  |  |
| --- | --- |
| Principal Investigator (Signature) | Date of Signature (DD/MMM/YYYY) |

CONTENTS

[Protocol Synopsis 7](#_Toc178522710)

[Table 1: Subject Visit Schedule 19](#_Toc178522711)

[1. Study background 23](#_Toc178522712)

[1.1 Foreword 23](#_Toc178522713)

[1.2 Drug profile 24](#_Toc178522714)

[1.3 TQB2450 Overview of Preclinical Pharmacodynamic Studies 24](#_Toc178522715)

[1.4 Overview of Preclinical Pharmacokinetics of TQB2450 25](#_Toc178522716)

[1.5 Overview of Preclinical Toxicology Studies of TQB2450 26](#_Toc178522717)

[1.5.1 General Pharmacology 26](#_Toc178522718)

[1.5.2 Acute toxicity test 27](#_Toc178522719)

[1.5.3 Long-term toxicity test 27](#_Toc178522720)

[1.5.4 Hemolysis and vascular stimulation test 28](#_Toc178522721)

[1.6 TQB2450 Human tolerance and kinetics and other clinical studies 29](#_Toc178522722)

[1.6.1 Safety and tolerability of TQB2450 in combination with anlotinib 30](#_Toc178522723)

[1.7 Preclinical Studies of anlotinib 31](#_Toc178522724)

[1.8 Anlotinib Toxicology Studies 31](#_Toc178522725)

[1.9 Study on the Dynamics of Anlotinib 32](#_Toc178522726)

[1.10 Safety and tolerability of anlotinib 33](#_Toc178522727)

[1.11 Study results of anlotinib in treatment of esophageal squamous cell carcinoma 36](#_Toc178522728)

[1.12 Current status of first-line treatment for esophageal squamous cell carcinoma 37](#_Toc178522729)

[2. Study objectives 40](#_Toc178522730)

[3. Study plan 40](#_Toc178522731)

[3.1 Study Design 40](#_Toc178522732)

[3.2 Study Duration 40](#_Toc178522733)

[3.3 Study Population 41](#_Toc178522734)

[3.3.1 Inclusion criteria 41](#_Toc178522735)

[3.3.2 Exclusion criteria 42](#_Toc178522736)

[3.3.3 Withdrawal Criteria 44](#_Toc178522737)

[3.3.4 Exclusion criteria 45](#_Toc178522738)

[3.3.5 Termination Criteria 45](#_Toc178522739)

[3.3.6 Withdrawal or treatment termination procedures 45](#_Toc178522740)

[3.4 Prohibited concomitant medications 46](#_Toc178522741)

[3.5 Concomitant medications allowing with caution 46](#_Toc178522742)

[3.6 Drugs and therapies that can be used concomitantly in the study 47](#_Toc178522743)

[4. Study drug 48](#_Toc178522744)

[4.1 Study Drug 48](#_Toc178522745)

[4.2 Dosing Regimen 49](#_Toc178522746)

[4.3 Dose Delays and Adjustments 51](#_Toc178522747)

[5. Collection of Biological Samples 54](#_Toc178522748)

[6. Study process 54](#_Toc178522749)

[6.1 Before study initiation 54](#_Toc178522750)

[6.2 During the study 55](#_Toc178522751)

[6.3 Dropouts 56](#_Toc178522752)

[6.4 End of treatment and follow-up 57](#_Toc178522753)

[6.4.1 End-of-treatment visit 57](#_Toc178522754)

[6.4.2 Follow-up Visit 57](#_Toc178522755)

[6.5 Unscheduled Visits 58](#_Toc178522756)

[7. Efficacy evaluation 58](#_Toc178522757)

[8. Safety evaluation 61](#_Toc178522758)

[8.1 Adverse Events 62](#_Toc178522759)

[8.2 Assessment of Adverse Events 62](#_Toc178522760)

[8.3 Recording of Adverse Events 63](#_Toc178522761)

[8.4 Follow-up of Adverse Events 65](#_Toc178522762)

[8.5 Criteria for judging the correlation between drug and adverse event 65](#_Toc178522763)

[8.6 Serious Adverse Events (SAEs) 66](#_Toc178522764)

[8. 7 Reporting system of serious adverse events 67](#_Toc178522765)

[8.8 Reporting procedures of SAEs 68](#_Toc178522766)

[8.9 Symptomatic treatment of common adverse reactions 68](#_Toc178522767)

[9. Quality of life 72](#_Toc178522768)

[10. Data Management and Statistical Analysis 72](#_Toc178522769)

[10.1 Case Report Forms 72](#_Toc178522770)

[10.2 Database establishment 73](#_Toc178522771)

[10.3 Data Lock 73](#_Toc178522772)

[10.4 Selection of Statistical Analysis Data 73](#_Toc178522773)

[10.5 Statistical Analysis Plan 73](#_Toc178522774)

[10.6 Sample Size Estimation 74](#_Toc178522775)

[This study is an exploratory study. The sample size calculation for the cohort 1 is based on a historical median PFS of 5.7 months with immunochemotherapy regimens in patients with advanced ESCC. It is hypothesized that the combination of benmelstobart, chemotherapy, and anlotinib would achieve an expected median PFS of 9.8 months. With a 12-month enrollment period and 12 months of follow-up, approximately 27 events among 38 patients are anticipated, providing 80% power at a two-sided α of 0.05 to demonstrate superior efficacy compared to the historical control. Considering a 20% dropout rate, the plan is to enroll 48 patients. 74](#_Toc178522776)

[11. Coordinator Investigator Responsibilities 74](#_Toc178522777)

[11.1 Co-organizer 74](#_Toc178522778)

[11.2 Investigator 75](#_Toc178522779)

[12. Ethical Guidelines and Informed Consent Form 75](#_Toc178522780)

[13. Quality control and quality assurance 76](#_Toc178522781)

[14. Study site and personnel 76](#_Toc178522782)

[14.1 Study Site, Site Number, and Principal Investigator (Sort by Site Number) 76](#_Toc178522783)

[14.2 Leading Site and Leader 77](#_Toc178522784)

[15. Discussion, approval and modification of study protocol 77](#_Toc178522785)

[16. Test Summary 77](#_Toc178522786)

[Attachment 1 TNM Staging of Esophageal Cancer (Version 8, 2017) 78](#_Toc178522787)

[Appendix 2 Evaluation of quality of life (ECOG PS) (ZPS 5 scale) 80](#_Toc178522788)

[Appendix 3 New York Heart Association (NYHA) Functional Classification 81](#_Toc178522789)

[Appendix 4 Creatinine Clearance Calculation 82](#_Toc178522790)

List of Abbreviations

| ACEI | | : | Angiotensin-converting enzyme inhibitor | | |
| --- | --- | --- | --- | --- | --- |
| AE | | : | Adverse Event | | |
| ASCO-GI | | : | American Society of Clinical Oncology Gastrointestinal Oncology Symposium | | |
| ALT | | : | Alanine aminotransferase (ALT) | | |
| ANC | | : | Neutrophils | | |
| APTT | | : | Activated partial thromboplastin time | | |
| ARB | | : | Angiotensin II Receptor Antagonists | | |
| ADR | | : | Adverse drug reaction | | |
| AST | | : | Aspartate aminotransferase (AST) | | |
| BUN | | : | Urea nitrogen | | |
| Ca | | : | Calcium | | |
| Cl | | : | Chlorine | | |
| Cr | | : | Creatinine | | |
| CR | | : | Complete response | | |
| CRF | | : | Case Report Form | | |
| CT | | : | Computed tomography | | |
| DCR | | : | Disease control rate | | |
| DoR | | : | Duration of Response | | |
| DLT | | : | Dose limiting toxicity | | |
| ECOG PS | | : | Performance status scoring criteria | | |
| EDC | | : | Electronic entry system | | |
| EGFR | | : | Epidermal growth factor receptor | | |
| Fbg | | : | Fibrinogen | | |
| GCP | | : | Good Clinical Practice | | |
| FAS | | : | Full Analysis Set | | |
| Glu | | : | Glucose | | |
| Hb | | : | Hemoglobin | | |
| HR | | : | Hazard ratio | | |
| HIV | | : | HIV | | |
| INR | | : | International Standardization | | |
| ITT | | : | Intent to treat set | | |
| K | | : | Potassium | | |
| LD 50 | | : | Median lethal dose | | |
| MRI | | | : | NMR | |
| MSI | | |  | Microsatellite instability | |
| Na | | | : | Sodium | |
| NCI-CTC | | | : | National Cancer Institute Common Toxicity Criteria | |
| OB | | | : | Occult blood | |
| ORR | | | : | Objective response rate | |
| OS | | | : | Overall survival | |
| PFS | | | : | No disease progression | |
| PD | | | : | Progression | |
| PDGFR | | | : | Platelet-derived growth factor receptor | |
| PI | | | : | Principal Investigator | |
| PLT | | | : | Platelets | |
| PR | | | : | Partial response | |
| PRO | | | : | Protein | |
| PPS | | | : | Per Protocol Set | |
| PT | | | : | Prothrombin time | |
| Q3W | | | : | Every 3 weeks | |
| Qd | | | : | Once daily | |
| RECIST | | | : | Response Evaluation Criteria in Solid Tumors | |
| SFDA | | | : | China Food and Drug Administration | |
| SAE | | | : | Serious adverse event | |
| SAS | | | : | Safety Analysis Set | |
| SD | | | : | Stable | |
| TT | | | : | Thrombin time | |
| TMB | | |  | Tumor mutation burden | |
| ULN | | | : | Upper limit of normal | |
| VEGF | | | : | Vascular endothelial growth factor | |
| VEGFR | | | : | Vascular endothelial growth factor receptor | |
| WBC | | | : | Leucocyte | |

**Protocol Synopsis**

| **Study title** | | Paclitaxel + cisplatin + TQB2450 with or without anlotinib in first-line treatment of advanced esophageal squamous cell carcinoma: a multicenter exploratory study |
| --- | --- | --- |
| **Protocol No.** | | TQB2450-II-13 |
| **Sponsor** | | Henan Cancer Hospital |
| **Co-organizer** | | Chia Tai Tianqing Pharmaceutical Group Co., Ltd. |
| **Nature of Study** | | Investigator-Initiated Exploratory Studies |
| **Study population** | | Patients with advanced esophageal squamous cell carcinoma |
| **Study**  **Purpose** | Primary objective | To evaluate the efficacy of paclitaxel + cisplatin + TQB2450 with or without anlotinib as first-line treatment in patients with advanced esophageal squamous cell carcinoma; |
|  | Secondary objective | To evaluate the safety of paclitaxel + cisplatin + TQB2450 injection with or without anlotinib as first-line treatment in patients with advanced esophageal squamous cell carcinoma; |
|  | Exploratory  Purpose | To explore the correlation between biomarkers and clinical efficacy. |
| **Efficacy evaluation** | | RECIST 1.1 criteria were used to determine disease status. |
| **Safety evaluation** | | The severity of adverse events was judged using NCI-CTC AE 5.0 criteria. |
| **Study Endpoints** | Primary endpoint | Progression-free survival (PFS) |
|  | Secondary endpoints | 1. Overall survival (OS), objective response rate (ORR), disease control rate (DCR), duration of response (DOR), quality of life score; 2. Incidence and severity of adverse events (AEs) and serious adverse events (SAEs), as well as abnormal laboratory test parameters; |
|  | Exploratory  Metrics | To explore the relationship between biomarkers such as PD-L1 expression, TMB, MSI and efficacy |
| **Study subjects** | | Patients with unresectable locally advanced, recurrent or metastatic esophageal squamous cell carcinoma |
| **Study Design** | | Multicenter, Open label, double arm, Phase II clinical trial |
| **Sample Size** | | This study is an exploratory study. The sample size calculation for the cohort 1 is based on a historical median PFS of 5.7 months with immunochemotherapy regimens in patients with advanced ESCC. It is hypothesized that the combination of benmelstobart, chemotherapy, and anlotinib would achieve an expected median PFS of 9.8 months. With a 12-month enrollment period and 12 months of follow-up, approximately 27 events among 38 patients are anticipated, providing 80% power at a two-sided α of 0.05 to demonstrate superior efficacy compared to the historical control. Considering a 20% dropout rate, the plan is to enroll 48 patients.  In the cohort 2, 30 patients will be enrolled. After completion of group 1, the study of group 2 will be conducted. |
| **Statistical methods** | | - Selection of Statistical Analysis Data   **Full Analysis Set (FAS):** The efficacy analysis was performed according to the intention-to-treat (ITT) principle in all patients who used the drug at least once.  **Per Protocol Set (PPS):** Patients with at least one tumor response evaluation results, who are compliant with the study protocol, have good compliance, have not received the prohibited drugs during the study, and have completed the case report form.  **Safety Analysis Set (SS):** All patients who used the investigational drug at least once and had safety record after treatment.   - Statistical Analysis Plan   **Efficacy Analysis:** For efficacy indicators progression-free survival (PFS), overall survival (OS), duration of response (D OR), median time will be estimated using the Kaplan-Meier method and events and their 95% confidence intervals will be presented. The disease control rate (DCR = CR + PR + SD) and objective response rate (ORR = CR + PR) were calculated using Clopper-Pearson method and presented with 95% confidence intervals. Quality of life score: Number and percentage of subjects with different levels of each dimension will be calculated for EORTC quality of life questionnaire (QLQ-C30), esophageal cancer module (QLQ-OES18) and EQ-5D. The signed-rank test is used for intra-group comparisons when necessary, and the H-test is used for inter-group comparisons.  **Safety analysis:** Safety analyses will be based on all treated subjects. All adverse events will be classified as NCI-CTCAE 5.0 Versions were graded, and descriptive statistics were mainly summarized, according to groups AE, SAE, ≥grade 3 AEs, drug-related AEs, drug-related SAEs, AEs leading to dose interruption / adjustment or treatment termination. The data are statistically summarized. Laboratory test results, vital signs, 12-lead electrocardiogram, echocardiography and other data, continuous indicators are analyzed by means, standard deviation, median, maximum, minimum and so on, and cross classification table is used for analysis of baseline and post-baseline conditions for qualitative data. |
| **Study interventions** | | **Cohort 1:**  **Initial treatment (4-6 cycles, decided by the investigator based on patient's tolerance): TQB2450 + anlotinib + paclitaxel + cisplatin**   - TQB2450 Injection: 1200 mg, D1, Q3W, the infusion time is 60±10 min. Three weeks is a treatment cycle. - Anlotinib: one tablet (10mg) once daily, continuous for 2 weeks and stop for 1 week, oral. Three weeks is a treatment cycle. - Paclitaxel: 135 mg/m^2^, day1 at every cycle, give hormone pretreatment before medication. Three weeks is a treatment cycle. - Cisplatin: Administer after paclitaxel, 60-75 mg/m^2^ intravenous drip. Administered on days 1 - 3 of each cycle. Three weeks is a treatment cycle.   **Maintenance treatment: TQB2450 + Anlotinib**   - TQB2450 Injection: 1200 mg, D1, Q3W, the infusion time is 60±10 min. Three weeks is a treatment cycle. - Anlotinib: one tablet (10mg) once daily, continuous for 2 weeks and stop for 1 week, oral. Three weeks is a treatment cycle.   **Cohort 2:**  **Initial treatment (4 to 6 cycles, decided by the investigator in combination with the patient's tolerance, so that the patient can receive initial treatment for 6 cycles): TQB2450 + paclitaxel + cisplatin**   - TQB2450 Injection: 1200 mg, D1, Q3W, the infusion time is 60±10 min. Three weeks is a treatment cycle. - Paclitaxel: 135 mg/m2, day1 at every cycle, give hormone pretreatment before medication. Three weeks is a treatment cycle. - Cisplatin: Administer after paclitaxel, 60-75 mg/m2 intravenous drip. Administered on days 1 - 3 of each cycle. **Maintenance treatment: TQB2450**    - TQB2450 Injection: 1200 mg, D1, Q3W, the infusion time is 60±10 min. Three weeks is a treatment cycle. |
| **Principal investigator** | | Prof. Suxia Luo |
| **Leading site** | | Henan Cancer Hospital |
| **Inclusion Criteria:**  Subjects can participate in the study only if all the following criteria are met:   1. Histopathologically confirmed, unresectable, locally advanced, recurrent, or metastatic esophageal squamous cell carcinoma (ESCC) (excluding mixed adenosquamous carcinoma); 2. No prior systemic therapy or have tumour recurrence more than 6 months after the completion of (neo) adjuvant or radical therapy (including radical surgery and radical chemoradiotherapy;   Note: Patients with advanced or recurrent non-target lesions who progressed again after radiotherapy alone were included. The time from the end of palliative treatment for local lesions (non-target lesions) to enrollment was more than 2 weeks.   1. At least one measurable lesion according to Response Evaluation Criteria in Solid Tumors version 1.1 (RECIST v1.1); Measurable lesions should not have received local treatment such as radiotherapy (lesions located in the area of previous radiotherapy can also be selected as target lesions if they are confirmed to have progressed and meet RECIST v1.1 criteria); 2. Age 18-75 years; 3. Eastern Cooperative Oncology Group-Performance status (ECOG-PS) of 0-1; Predicted life expectancy of ≥3 months; 4. Adequate function of the important organs as evidenced by the following: 5. Hemanalysis:  - hemoglobin (Hb) ≥90g/L (no blood transfusion within 28 days); - absolute neutrophil count (ANC) ≥1.5×10^9^/L; - platelets (PLT) ≥100×10^9^/L.  1. Biochemistry:  - total bilirubin (TBIL) ≤1.5×upper limit of normal (ULN)； - alanine aminotransferase (ALT) and aspartate aminotransferase (AST) ≤2.5×ULN; ALT and AST ≤5×ULN in patients with liver metastases; - Creatinine (Cr) ≤1.5×ULN and creatinine clearance rate (CCr) ≥60 mL/min (Cockcroft-Gault formula).  1. Adequate coagulation function: international normalization ratio (INR), or prothrombin time (PT) ≤1.5×ULN; 2. Women of reproductive age were required to use appropriate contraception from the time of screening until 3 months after discontinuation of study treatment and were not breast-feeding. A negative pregnancy test or one of the following criteria before the initiation of dosing proved that there was no risk of pregnancy:   a. Postmenopausal status was defined as age ≥50 years and amenorrhea for at least 12 months after discontinuation of all exogenous hormone replacement therapy;  b. Women aged <50 years were also considered postmenopausal if they had amenorrhea for 12 months or more after discontinuation of all exogenous hormone therapy and the luteinizing hormone (LH) and follicle-stimulating hormone (FSH) were accorded with the laboratory normal reference range;  c. Patients who had undergone irreversible sterilization procedures, including hysterectomy, bilateral oophorectomy, or bilateral salpingectomy, with the exception of those who had bilateral tubal ligation.  For men, consent is given to use an appropriate method of contraception or to have been surgically sterilized during the trial period and 8 weeks after the last drug administration.   1. Be willing and able to provide written informed consent for the trial, and have better compliance with follow-up. | | |

| **Exclusion Criteria**:  Subjects who meet any of the following criteria are not eligible to enter the study:   1. Patients with ESCC who have complete obstruction under endoscopic guidance and need interventional therapy to relieve obstruction; 2. Patients with ulcerative ESCC;   Note: This refers primarily to patients with ulcers adjacent to blood vessels that increase the risk of bleeding.   1. Patients after esophageal or tracheal stent placement; 2. Patients with a high risk of bleeding or perforation due to tumor invasion of adjacent organs (large arteries or trachea) of the esophageal lesion, or with established fistulas; 3. Patients who had hematemesis, bloody stool and daily blood loss ≥2.5 mL or any CTCAE grade ≥3 bleeding events within 3 months before screening, or who had any evidence of bleeding, regardless of severity, or whose history was judged by the investigator to be ineligible for enrollment; 4. Patients who have allergic reactions to drug formulations or excipient components or similar drugs; 5. Patients who had received adjuvant chemotherapy with paclitaxel and had recurrence or metastasis within one year;   Note: Patients with recurrence or metastasis for more than one year could be included in the study.   1. Factors significantly affecting oral medication (e.g. swallowing difficulty, chronic diarrhea, and intestinal obstruction); 2. The liver metastatic burden with accounting for approximately more than 50% of the total liver volume; 3. Patients with any severe and/or uncontrolled illness, including:  - Patients with poor blood pressure control using antihypertensive drugs (systolic blood pressure ≥150 mmHg or diastolic blood pressure ≥100 mmHg); patients with grade II or above myocardial ischemia or myocardial infarction, arrhythmia (including QT interval ≥480ms); patients with Grade III-IV cardiac insufficiency, or with left ventricular ejection fraction (LVEF) <50% via the cardiac color ultrasound; - Active or uncontrolled severe infection; - Liver diseases such as cirrhosis, decompensated liver disease, chronic active hepatitis; - Poor diabetes control (fasting blood glucose [FBG] >10 mmol/L); - Urinary protein ≥ ++, and confirmed 24-hour urinary protein >1.0 g;  1. Unhealed wound or fracture for a long time; 2. Patients with ESCC who have active bleeding of the primary lesion within 2 months; NCI CTCAE grade >1 pulmonary hemorrhage within 4 weeks prior to enrollment; NCI CTCAE grade >2 other site bleeding within 4 weeks prior to enrollment; patients with bleeding tendencies (e.g., active gastrointestinal ulcers) or those receiving thrombolytic or anticoagulation therapy such as warfarin, heparin, or similar agents; 3. Patients who have undergone major surgical procedures (e.g., craniotomy, thoracotomy, or laparotomy) within 4 weeks before the first study dose or are anticipated to require major surgery during the study treatment; 4. Patients with a history of gastrointestinal perforation and/or fistula within 6 months prior to enrollment, or with thromboembolic events such as cerebrovascular accidents (including transient ischemic attacks), deep vein thrombosis, and pulmonary embolism; 5. Known presence of symptomatic central nervous system metastases and/or carcinomatous meningitis; 6. Clinically significant ascites, including ascites detectable on physical examination, ascites that have been treated previously or currently require treatment, and minimal ascites evident by imaging only but without symptoms; 7. Patients with moderate bilateral pleural effusion, or significant pleural effusion on one side, or those who have developed respiratory impairment requiring drainage; 8. Known active pulmonary tuberculosis; 9. Interstitial lung disease requiring steroid hormone therapy; 10. Uncontrolled metabolic disturbances or other non-malignant or systemic disease or secondary reaction to cancer, which can lead to higher medical risks and/or uncertainty of survival evaluation; 11. Patients with significant malnutrition; 12. Patients with a history of psychotropic substance abuse and difficulty achieving abstinence or with psychiatric disorders; 13. Patients with a history of immunodeficiency, including those with a positive HIV test or suffering from other acquired or congenital immunodeficiency diseases, or those with a history of organ transplantation; 14. History of other primary malignant tumors, except for the following: 1) Complete remission of malignant tumors for at least 2 years prior to enrollment, without further treatment during the study; 2) Adequately treated non-melanoma skin cancer or lentigo maligna with no evidence of disease recurrence; 3) Adequately treated carcinoma in situ with no evidence of disease recurrence; 15. Pregnant or lactating women; 16. > Grade 1 Unresolved toxicity according CTCAE due to any previous treatment, excluding alopecia; 17. Patients who have received first-line chemotherapy for advanced disease or radiotherapy prior to first dose;   Note: Patients who previously received local radiotherapy can be eligible if: the end of radiotherapy is more than 3 weeks from the start of study treatment; the target lesion selected for this study is not within the radiation field; or the target lesion is located within the radiation field, but progression has been confirmed; without chemotherapy, immunotherapy and targeted therapy during radiotherapy.   1. Received treatment with Chinese patent medicines with anti-tumor indications as specified in the approved NMP-approved drug instructions (including Fufangbanmao Capsules, Kangai Injection, Kanglaite Capsules / Injection, Aidi Injection, Brucea javanica oil Injection/Capsules, Xiaoaiping Tablets/Injection, Huachansu Capsules, etc.) within 2 weeks prior to the first administration of the medication. 2. Patients previously treated with VEGFR small molecule inhibitors, such as anlotinib, apatinib, lenvatinib, sorafenib, sunitinib, regorafenib and furazolidone; 3. Patients previously received treatment with anti-PD-1 or anti-PD-L1/PD-L2 inhibitors or other therapies acting on T cell costimulatory targets or checkpoints; 4. History of live attenuated vaccination 28 days prior to first dose or planned live attenuated vaccination during the study; 5. Active autoimmune disease requiring systemic therapy (eg, disease-modifying drugs, corticosteroids, or immunosuppressants) within 2 years prior to first dose. Replacement therapy (e.g., thyroxine, insulin, or physiological corticosteroids for adrenal or pituitary insufficiency, etc.) is not considered as systemic therapy; 6. Diagnosis of immunodeficiency or ongoing systemic glucocorticoid therapy or any other form of immunosuppressive therapy (>10 mg/ Prednisone or other effective hormone), and continued use within 2 weeks prior to the start of study treatment; 7. Participated in other clinical trials of anti-tumor drugs within 4 weeks prior to first dose (the washout period is calculated from the end time of the last treatment); 8. Patients who have concomitant diseases that seriously jeopardize the patient's safety or affect the patient's completion of the study, or are considered unsuitable for enrollment for other reasons according to the judgment of the investigator.   **Withdrawal Criteria**   1. The patients demonstrate poor compliance, regularly failing to adhere to the prescribed medication regimen (patients do not take medication at the planned time and dose more than two times for no reason, and the investigator's judgment will seriously affect the following study and study results); 2. Treatment with other systemic antineoplastic agents (e.g., chemotherapy, hormone therapy, targeted therapy, or biologic agents) that affect the judgment of efficacy; 3. Patients who experienced serious adverse events (SAEs) and were not suitable for further study according to the investigator’s judgment or who had an unintended pregnancy; 4. Patients who are unwilling to continue the clinical trial and insist on withdrawing; 5. The investigators deemed it necessary to stop the study.   **Removal criteria**   1. Patients who receive chemotherapy, surgery or investigational drug beyond the protocol during the trial; 2. Patients who failed to meet the inclusion criteria were mistakenly included; 3. Patients without medication;   Note: Patients who met criteria 1-2 were included in the safety analysis.  **Termination Criteria**  Subjects must be terminated from the study if they meet any of the following criteria:   1. Intolerance during treatment and ineligibility for continueding treatment; 2. Disease progression; 3. Other reasons that the investigator considers inappropriate for continuation of study treatment; 4. Termination by investigators. | |
| --- | --- |
| Duration of trial | June 2021-July 2023. |

**Table 1: Subject Visit Schedule**

| **Visit/Program** | **Screening Period** | | | **Treatment period** | | | | | | | | | | **Follow-up Period** | |
| --- | --- | --- | --- | --- | --- | --- | --- | --- | --- | --- | --- | --- | --- | --- | --- |
|  |  |  |  | **Initial Treatment Period** | | | | | | **Maintenance Treatment Period** | | | **End of treatment**  **Visits** | **Safety ^[22]^** | **Survival ^[23]^** |
|  | **- 28 to -1d** | | **- 7 to -1d** |  |  |  |  |  |  |  |  |  |  |  |  |
| Treatment period | - | | - | C1 | C2 | C3 | C4 | C5 | C6 | C7 | C8 | C9 and thereafter | - | 28 days after last medication /Initiation of new anticancer therapy | Per 8 Week |
| Window period ^[1]^ | NA | | NA | D21 ± 3 | | | | | | | | | + 7 | ± 7 | ± 7 |
| Informed Consent ^[2]^ | **×** | |  |  |  |  |  |  |  |  |  |  |  |  |  |
| Tumor history, other disease history, treatment history ^[3]^ | **×** | |  |  |  |  |  |  |  |  |  |  |  |  |  |
| Inclusion and exclusion criteria check | **×** | | **×** |  |  |  |  |  |  |  |  |  |  |  |  |
| Pregnancy test ^[4]^ |  | | **×** |  |  |  |  |  |  |  |  |  |  |  |  |
| Hepatitis B/Hepatitis C/HIV test | **×** | |  |  |  |  |  |  |  |  |  |  |  |  |  |
| Evidence of radiographic progression ^[5]^ | **×** | |  |  |  |  |  |  |  |  |  |  |  |  |  |
| Echocardiogram ^[6]^ |  | | **×** |  |  |  |  |  |  |  |  |  | **×** |  |  |
| ECOG PS score |  | | **×** | **×** | **×** | **×** | **×** | **×** | **×** | **×** | **×** | **×** | **×** |  |  |
| Life Quality Questionnaire |  | | **×** |  | **×** |  | **×** |  | **×** |  |  | **×** | **×** |  |  |
| Blood pressure ^[7]^ |  | | **×** | **×** | **×** | **×** | **×** | **×** | **×** | **×** | **×** | **×** | **×** |  |  |
| Coagulation function, Thyroid function ^[8]^ |  | | **×** | **×** | **×** | **×** | **×** | **×** | **×** |  | **×** |  | **×** |  |  |
| Amylase, Lipase |  | | **×** | **×** | **×** | **×** | **×** | **×** | **×** |  | **×** |  | **×** |  |  |
| Blood routine ^[9]^ |  | | **×** | **×** | **×** | **×** | **×** | **×** | **×** | **×** | **×** | **×** | **×** |  |  |
| Blood biochemistry ^[10]^ |  | | **×** | **×** | **×** | **×** | **×** | **×** | **×** | **×** | **×** | **×** | **×** |  |  |
| Urine routine ^[11]^ |  | | **×** | **×** | **×** |  | **×** |  | **×** |  | **×** |  | **×** |  |  |
| Stool routine ^[12]^ |  | | **×** | **×** | **×** |  | **×** |  | **×** |  | **×** |  | **×** |  |  |
| Electrocardiogram ^[13]^ |  | | **×** | **×** | **×** | **×** | **×** | **×** | **×** | **×** | **×** | **×** | **×** |  |  |
| Markers of myocardial injury ^[14]^ |  | | **×** | **×** | **×** | **×** | **×** | **×** | **×** | **×** | **×** | **×** | **×** |  |  |
| Vascular endothelial growth factor VEGF |  | | **×** |  | **×** |  | **×** |  | **×** |  |  | **×** | **×** |  |  |
| Tumor markers ^[15]^ |  | | **×** |  | **×** |  | **×** |  | **×** |  |  | **×** | **×** |  |  |
| Lymphocyte immunoassay |  | | **×** |  | **×** |  | **×** |  | **×** |  |  | **×** | **×** |  |  |
| Tumor imaging examination and evaluation ^[16]^ | **×** | |  |  | **×** |  | **×** |  | **×** |  |  | **×** | **×** |  |  |
| Vital Signs and Physical Examination ^[17]^ |  | | **×** | **×** | **×** | **×** | **×** | **×** | **×** | **×** | **×** | **×** | **×** |  |  |
| Study drug ^[18]^ | | | | | | | | | | | | | | | |
| Biomarker - Tissue ^[19]^ | **×** |  | |  | | | | | |  | | |  |  |  |
| Paclitaxel |  |  | | **×** | | | | | |  | | |  |  |  |
| Epilepsy |  |  | | **×** | | | | | |  | | |  |  |  |
| TQB2450 |  |  | | **×** | | | | | | **×** | | |  |  |  |
| Anlotinib |  |  | | **×** | | | | | | **×** | | |  |  |  |
| Documentation of co-administration ^[20]^ | **×** | **×** | | **×** | | | | | | **×** | | | **×** | **×** |  |
| Records of Adverse Events ^[21]^ | **×** | **×** | | **×** | | | | | | **×** | | | **×** | **×** |  |

Note: In addition to the examination items and time points in the table, the investigator may add visits and other examination items as needed, and the examination results should be filled in the corresponding part of case report form (eCRF).

[1]. The allowable window period for imaging examination, safety follow-up and survival follow-up is ±7 days, +7 days for exit visit, ± 3 days for other examinations, in addition, the medication window is ± 3 Day.

[2]. Informed Consent: Performed within 28 days prior to enrollment.

[3]. Tumor history and other disease history: pathological results; tumor surgery, chemotherapy, radiotherapy and treatment history of other diseases. Completed within 28 days prior to enrollment.

[4]. Pregnancy test was limited to women of childbearing age and was completed 7 days prior to enrollment.

[5]. Radiographic evidence of postoperative disease recurrence or progression was collected and completed 28 days prior to enrollment.

[6]. Echocardiogram: Performed once 7 days before enrollment, and the examination should be supplemented when clinically significant ECG abnormalities occurred during treatment, and at check-out visit.

[7]. Blood pressure monitoring: Blood pressure monitoring is done and recorded by the patient himself/herself, and blood pressure is monitored regularly weekly. If the blood pressure was abnormal, it was tested daily. At each follow-up visit, blood pressure was measured by the investigator. For blood pressure measurement, smoking and coffee drinking were prohibited for 30 minutes before measurement, and at least 10 minutes of quiet rest was taken; the measurement was done in a seated position, with the elbow placed at the same level as the heart, and the same side was taken for each blood pressure measurement.

[8]. Coagulation function: PT, APTT, TT, Fbg, INR, D-Dimer; Thyroid function: thyroid stimulating hormone (TSH), triiodothyronine T3, thyroxine T4. Once within 7 days prior to enrollment, once during initial treatment Once every cycle, once every even cycle during maintenance and once during the out visit.

[9]. Blood routine: red blood cell count (RBC), hemoglobin (Hb), hematocrit (HCT), platelet count (PLT), white blood cell count (WBC), neutrophil count (NEUT), lymphocyte count (LYM), monocyte count (MONO), eosinophil count (EOS), basophil count (BASO). Once 7 days prior to enrollment, once every cycle during the treatment period and once at the out visit. If neutrophils ≤ 1 × 10 ^9^ /L or platelets ≤ 50 × 10 ^9^ /L, the frequency of re-examination should be increased (once every 2-3 days). In case of dose delay or dose adjustment due to hematological toxicity in any cycle, blood routine should be re-examined every week;

[10]. Blood chemistry: Hepatic function: Alanine aminotransferase (ALT), aspartate aminotransferase (AST), glutamyl transpeptidase (GGT), total bilirubin (TBIL), direct bilirubin (DBIL), alkaline phosphatase (ALP), total protein (TP), albumin (ALB); Renal function: Blood urea nitrogen (BUN)/ Urea ( UREA ) Creatinine (Cr) ; Glucose (GLU); Electrolytes: potassium (K ), sodium (Na ), chlorine (Cl ), calcium (Ca ), magnesium (Mg ), inorganic phosphorus (PHOS ); lipid profile four items: total cholesterol (TC ), triglycerides (TG ), high-density lipoprotein (HDL ), low-density lipoprotein (LDL ); uric acid (UA). The examination will be performed once every cycle during the treatment period and once at the out visit.

[11]. Urinalysis: urine protein, urine glucose, urine occult blood (urine red blood cells, white blood cells), urine pH and urine ketone body. If the semi-quantitative method shows ≥ 2 + urine protein (e.g., urine dipstick), 24-hour urine protein quantification test will be performed. Once within 7 days prior to enrollment, once in Cycle 1 (C1D21), and then once every even cycle (C2D21/C4D21...), once at out visit.

[12]. Stool routine: Fecal leukocytes (WBC), fecal erythrocytes (RBC), containing occult blood. Once within 7 days prior to enrollment, once in Cycle 1 (C1D21), and then once every even cycle (C2D21/C4D21...), once at out visit.

[13]. ECG: performed once within 7 days prior to enrollment, once every cycle during treatment and at out visit. ECG should be confirmed twice when abnormality (at an interval of 5 minutes, the QTc interval should be indicated).

[14]. Markers of myocardial injury: creatine kinase (CK), creatine kinase isoenzyme (CK-MB), cardiac troponin T and/or I, B Type natriuretic peptide (BNP).

[15]. Tumor markers: scale-cell carcinoma antigen (SCC), carcinoembryonic antigen (CEA) =;

[16]. Tumor imaging examination and evaluation: Neck, chest, abdomen and pelvic cavity + enhancement CT or MRI. Enhanced MRI of brain scans should be done when brain metastasis is suspected or confirmed, and bone scanning is performed only when clinically indicated.

✓ Screening period: Tumor assessment may be completed no later than 1 week before the first dose of study drug. Imaging results must be obtained before signing the informed consent. The result may be used for tumor assessment during screening phase at the discretion of the investigator if the assessment meets the requirements of RECIST 1.1 and is less than 4 weeks before first study drug administration

✓ Study Treatment Period: During initial treatment Tumor imaging was performed every 2 cycles, and every 3 cycles during the maintenance period. Timely imaging is required when subjects discontinue study treatment (+ 7 days, if the previous examination time is not more than 4 weeks from the termination of treatment, it is not necessary to perform the examination again at the time of termination). Conditions for imaging examination should be the same as baseline (including scanning slice thickness, contrast agent, etc.). The allowable window period for imaging examination is + 7 days. Unscheduled imaging may be performed when disease progression is suspected (e.g., symptomatic deterioration).

✓ During the safety follow-up period: For subjects in whom no radiographic progression is observed, radiographic assessments should still be performed at the same frequency until disease progression or initiation of other antineoplastic therapy.

✓ In addition to radiographically confirmed disease progression, subjects who discontinue study treatment for other reasons should also undergo imaging examination as frequently as possible per protocol until documented disease progression, initiation of new antineoplastic therapy, or death.

[17]. Physical examination: examination of major body systems (general condition, mucocutaneous, head, neck, chest, abdomen, spine/extremities, other). Vital signs examination: body temperature, respiratory rate, heart rate and body weight. Once within 7 days prior to enrollment, once every cycle during the treatment period and once at the out visit.

[18]. Study Drug:

TQB2450 Injection: 1200 mg/time, administered on d1 of each cycle, diluted to 250 mL with normal saline, infusion time of 60 ± 10 min, 3 weeks was a treatment cycle.

Paclitaxel: 135 mg/m ^2^ on d1 of each cycle, hormone pretreatment was given before treatment, and 3 weeks was a treatment cycle.

Z: 60 to 75 mg/m^2^ intravenous after paclitaxel, divided into d1-d3 medication in each cycle; 3 weeks as a treatment cycle.

Anlotinib: Experimental arm 1 requires combination with anlotinib. One capsule (10 mg) once daily, administered on d1-d14 of each cycle followed by 1-week break, with 3 weeks as a treatment cycle.

[19]. Biomarker test - tissue: The patient shall provide tissue samples or sections for PD-L1, TMB and MSI detection.

[20]. Documentation of co-administration: Documentation of co-administration and concomitant therapy within 28 days prior to enrollment to 28 days after the last dose/start of new antitumor therapy

[21]. Records of Adverse Events: Adverse events were recorded from the start of signed informed consent to the end of the safety follow-up period.

[22]. Safety follow-up: After the end of treatment visit, enter the safety follow-up period to track the resolution of adverse events. From the completion of the study visit until 28 days after the last dosing or until initiation of other antineoplastic therapy, whichever occurred first, was the safety follow-up period.

[23]. Survival follow-up: After completion of the safety follow-up period, subjects will enter the survival follow-up period. The first survival follow-up (including telephone follow-up) is performed 8 weeks (±7 days) after the last safety follow-up and is conducted at a frequency of once every 8 weeks (±7 days) until the subject is deceased, lost to follow-up, study termination by the sponsor, or other termination criteria are met, whichever occurs first.

Fig. 1: Study Design Chart


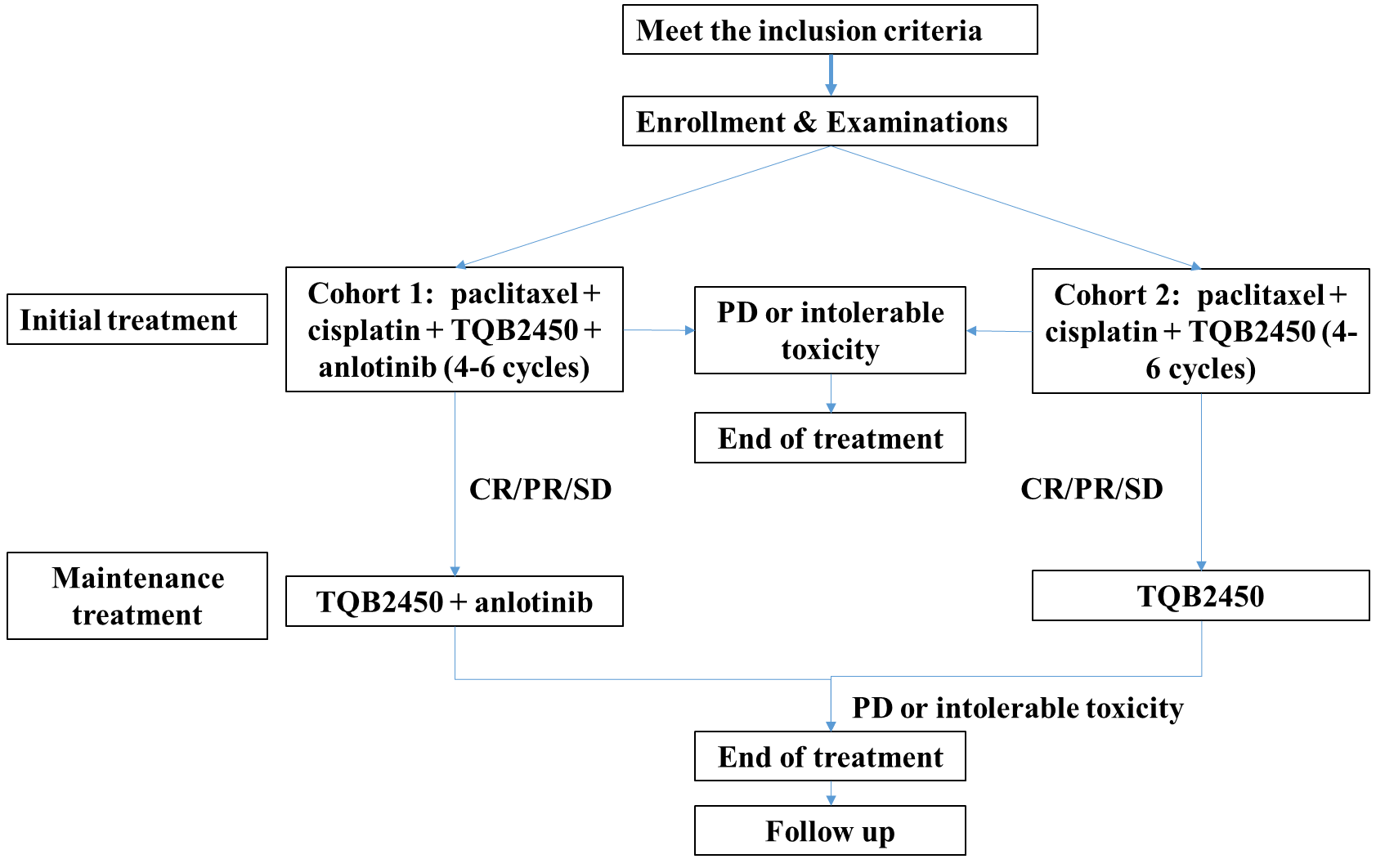


1. Study background

1.1 Foreword

Anlotinib hydrochloride is a Class 1.1 new drug independently developed in China and a novel small molecule multi-target tyrosine kinase inhibitor against angiogenesis. In March 2011, CFDA obtained the Clinical Approval Letter (CFDA, Approval No.: 2011L00661) and approved to carry out the clinical study. The ALTER 0303 study demonstrated that the use of anlotinib versus placebo as third-line or greater therapy for advanced NSCLC significantly prolonged both PFS and OS. Clinical studies of Anlotinib Hydrochloride carried out at the same time have shown that it is effective in the treatment of advanced soft tissue sarcoma, small cell lung cancer, medullary thyroid cancer, differentiated thyroid cancer and other tumors.

Programmed death factor 1 and its ligand (PD-1/PD-L1) are a pair of immune costimulatory factors. Normally, PD-1 plays an immunoregulatory role through its ligand PD-L1. In recent years, PD-1 and its ligand PD-L1 have received attention because of their involvement in tumor immune escape mechanisms. Activation of PD-1/PD-L1 signaling pathway can lead to the formation of immunosuppressive tumor microenvironment, allowing tumor cells to evade immune surveillance and killing, while blocking PD-1/PD-L1 signaling pathway can reverse tumor immune microenvironment and enhance endogenous anti-tumor immune effect. PD-L1 is highly expressed in various solid malignancies, including cancer of the lung and esophagus, Melanoma, renal cell carcinoma, prostate cancer, breast cancer, glioma, etc., and their expression levels vary according to the tumor type. It can not only promote the growth of tumor cells and also induce the apoptosis of T lymphocytes.

Nowadays, numerous immunotherapy clinical studies are in full swing. In mechanistic studies, tumor vascular abnormalities promote tissue hypoxia and increased lactate, which activate immunosuppression and inhibit T cell function. Anti-angiogenic drugs then enhance the immersion of effector immune cells by inducing vascular normalization and reducing immunosuppression Moistening. Based on this, this present study was designed to investigate TQB-2450 (PD-L1) in combination with anlotinib.

1.2 Drug profile

TQB-2450 It is a targeted programmed death ligand - 1 (PD-L1) of humanized monoclonal antibodies to prevent PD-L1 AND T Cell surface PD-1 And B7.1 Receptor binding, such that T Cells restore activity, thereby enhancing the immune response, with the potential to treat many types of tumors.

Molecular formula: C_6444_ H_9968_ N_1692_ O_2002_ S_46_

Molecular weight: 144,651 Da (aglycosylated)

Anlotinib hydrochloride capsules are hard capsules. The structural formula of the main component, Anlotinib, is shown in the figure below:

Molecular formula: C_23_ H_22_ FN_3_ O_3_ · 2HCl

Molecular weight: 480.36

1.3 TQB2450 Overview of Preclinical Pharmacodynamic Studies

Results of in vitro pharmacodynamic studies:

TQB2450 and Tecentriq® could bind to human PD-L1 and show equivalent binding activity with EC50 of 21.3 ng/mL and 27.6 ng/mL, respectively.

Both TQB2450 and Tecentriq® significantly induced IFN-γ secretion in MLR with DC cultured with CD4+ cells in a dose-dependent manner. Basically, equivalent induction to stimulation in TQB2450 (EC50, 35.0±11.3 ng/mL) and Tecentriq® (EC50, 26.6±1.4 ng/mL) indicated that the binding between TQB2450 and PD-L1 expressed on DC cells could inhibit PD-L1/PD-1 pathway and stimulate the secretion of IFN-γ in CD4+ T cells.

In the mixed reaction assay of DC and Tregs, IFN-γ was barely secreted, and TQB2450 showed no stimulatory activity on the IFN-γ. However, the opposite trend was observed in the Treg-free mixed reaction assay of DC and CD4+ CD25-T cells. If Treg, CD4+ CD25-T cells, and DC cells were mixed, Treg significantly inhibited IFN-γ secretion, while TQB2450 remarkably reversed the inhibitory activity of Treg. Overall, TQB2450 showed comparable activity to the control antibody Tecentriq®.

Neither TQB2450 nor Tecentriq® showed significant ADCC activity against MC-38/H-11 cells.

In vivo pharmacodynamic study results:

The antitumor rates of TQB2450 (1.5, 5, 15 mg/kg, IP, Q2D×11) on the subcutaneously transplanted tumor of MC-38/H-11 mice were 95.8%, 63.0%, and 91.7%, respectively. The results were calculated based on the anti-tumor rate (93.8%) of Tecentriq® (15 mg/kg, IP, Q2D×11) on MC-38/H-11 mice (all calculated per the median tumor volume). The therapeutic effect of TQB2450 on the subcutaneously transplanted tumor of MC-38/H-11 mice with colon cancer was comparable to that of Tecentriq®.

TQB2450 (1.5, 5, 15 mg/kg, IP, Q2D×11) significantly prolonged the survival of mice intraperitoneally inoculated with MC-38/H-11 cells. The median survival time was >98 days, and the survival rate to the end of the experiment (D98) was 70%, 80%, and 80%, respectively (P<0.01 compared with the human IgG 15 mg/kg group). The results are calculated based on the significantly prolonged survival, the median survival time of >98 days, and the survival rate of 100% of Tecentriq® (15 mg/kg, IP, Q2D×11) on mice with abdominal tumors. The efficacy (including survival time and survival rate) of TQB2450 on MC-38/H-11 mice with colon cancer was comparable to those of Tecentriq®.

TQB2450 of 5-10 mg/kg (22 days, 3 times a week) and 10 mg/kg (22 days, once a week) by tail vein injection could inhibit the growth of transplanted tumors of A375 human melanoma in NGG mice in a dose-dependent manner. 10 mg/kg of TQB2450 at different administration frequencies (3 times a week and once a week) had the equivalent antitumor effect as the positive control of ATEZOLIZUMAB (3 times a week). The antitumor effect of TQB2450 may be related to the activation of human T cells and the increased release of IFN-γ in tumors. In vitro and in vivo pharmacodynamic studies confirmed that TQB2450 could prevent PD-L1 from binding to the PD-1 and B7.1 receptors on the T cells, and restore the activity of T cells, thus enhancing the immune response and playing the antitumor role.

1.4 Overview of Preclinical Pharmacokinetics of TQB2450

After single intravenous doses of TQB2450 at 1, 10, and 60 mg/kg, pharmacokinetic data in cynomolgus monkeys showed a linear dose-dependent relationship. Following multiple doses (10 mg/kg, once a week on weeks 1-4), there were no statistical differences in the peak time and half-life compared with a single dose. However, the peak concentration and drug exposure were higher than that of a single dose. Besides, the accumulation factor was 1.73±0.65 at the interval of 168 hr.

The radioactive distribution of TQB2450 was ranked by the area under the curve (AUC): serum, lung, liver, bone marrow, gonads, heart, adrenal gland, spleen, kidney, bladder, lymph nodes, small intestine, submandibular gland, fat, thymus, eyeball, large intestine, urine, pancreas, muscle, brain. Tissues and organs with abundant blood perfusion had greater radioactivity. In contrast, organs with poor blood perfusion had less radioactivity. Low radioactivity in the brain suggested that TQB2450 did not readily cross the blood-brain barrier.

TQB2450 was mainly excreted through urine and slightly through feces. The excretion rate of TQB2450 was slow.

1.5 Overview of Preclinical Toxicology Studies of TQB2450

1.5.1 General Pharmacology

Tissue cross-reactivity:

The degree of tissue cross-reactivity was assessed by a two-step method for immunohistochemistry. The results revealed that TQB2450 had specific tissue cross-reactivity with 18 normal tissues and 15 tissues of cynomolgus monkeys. The details were as follows:

**Table 2 Tissue cross-reactivity**

| Species | Group | Cross-reactive tissue |
| --- | --- | --- |
| Human | TQB2450 | The pituitary gland, brain, cerebellum, lung, liver, skeletal muscle, heart, colon, small intestine, stomach, kidney, bladder, lymph nodes, spleen, thymus, bone marrow, blood cells, and placenta. |
|  | Homotype negative control | Lungs, skeletal muscles, colon, bladder, kidney, stomach, heart, blood cells, and bone marrow. |
| Cynomolgus monkeys | TQB2450 | The pituitary gland, lung, liver, muscle, heart, colon, small intestine, stomach, kidney, bladder, lymph nodes, spleen, thymus, bone marrow, and blood cells. |
|  | Homotype negative control | Kidneys, blood cells, and bone marrow. |

**Effects on cardiovascular system in cynomolgus monkeys (concomitant safety pharmacology study)**

The cynomolgus monkeys were divided into 4 groups: control group, TQB2450 50 mg/kg group, TQB2450 100 mg/kg group, and TQB2450 200 mg/kg group. Each group had 5 male cynomolgus monkeys and 5 females. Each group was intravenously given control substances (placebo injection without TQB2450) or a corresponding concentration of TQB2450 at the volume of 20 mL/kg. Results showed that the electrocardiographic parameters, blood pressure, and respiratory rate of female and male monkeys in each group had no obvious abnormal alterations.

1.5.2 Acute toxicity test

Injection in cynomolgus monkeys:

Six cynomolgus monkeys were divided into 2 groups with 3 (both male and female) in each group. The two groups were given TQB2450 of 200 and 400 mg/kg, respectively, and the two were delivered single intravenous doses (10 mg/mL) of TQB2450 at the volume of 20 or 40 mL/kg, respectively. The day of first dose was the first day of the study. Results indicated that general observation, body mass, food intake, temperature, electrocardiogram, blood pressure, hemanalysis, serum biochemistry, urinalysis, and gross anatomy of cynomolgus monkeys showed no obvious abnormalities following single intravenous doses of TQB2450 of 200 and 400 mg/kg. The maximum tolerated dose (MTD) was 400 mg/kg.

1.5.3 Long-term toxicity test

4-week toxicity and toxicokinetics after intravenous injection in cynomolgus monkeys

Cynomolgus monkeys were divided into a control group, TQB2450 50 mg/kg group, TQB2450 100 mg/kg group, and TQB2450 200 mg/kg group with 5 males and 5 females in each group. Each group was intravenously given control substances (placebo injection without TQB2450) or a corresponding concentration of TQB2450 at the volume of 20 mL/kg. The drugs were delivered once a week for 5 consecutive weeks, and the infusion rate was ~2 mL/min. Then, the drug was suspended for 8 weeks.

TQB2450 had certain immunogenicity. The antibody-positive rates in 50, 100, and 200 mg/kg groups were 10%, 10%, and 0, respectively, and were detected within 6-8 weeks after discontinuance. The exposure of TQB2450 in cynomolgus monkeys increased proportionally with the increasing dose between 50 mg/kg and 200 mg/kg. Besides, certain accumulation was observed after multiple doses.

TQB2450 was injected intravenously into cynomolgus monkeys for 4 weeks and suspended for 4 weeks. The no-observed-adverse-effect level (NOAEL) was 200 mg/kg (AUC0-t was 507909.7±139794.1 hr*μg/mL at this dose after 5 doses).

Table 3 Mean toxicokinetic parameters after administration of different doses of TQB2450 in cynomolgus monkeys

| Time | Dose (mg/kg) | | TQB2450 | | |
| --- | --- | --- | --- | --- | --- |
|  |  |  | 50 | 100 | 200 |
| First dose | C_max_ | μg/mL | 1112.9±166.7 | 2122.4±382.1 | 4120.8±476.4 |
|  | AUC_0-t_ | μg·h/mL | 69524.6±10057.9 | 136371.1±20213.0 | 272611.4±45291.7 |
| Last dose | C_max_ | μg/mL | 1663.5±325.1 | 3343.6±810.9 | 6062.6±1245.5 |
|  | AUC_0-t_ | μg·h/mL | 139614.2±27136.6 | 281160.2±97989.0 | 507909.7±139794.1 |
| Accumulation factor | AR | — | 2.0±0.4 | 2.0±0.5 | 1.9±0.4 |

1.5.4 Hemolysis and vascular stimulation test

Hemolysis test

The hemolysis test consisted of a negative control group (0.9% sodium chloride injection), a positive control group (sterilization water for injection), and TQB2450 dose groups (0.1, 0.2, 0.3, 0.4, 0.5 mL/tube, 10 mg/mL, respectively). The results showed that after 3 hours at 37±0.5℃, hemolysis and coagulation were not observed in the negative control group, while hemolysis was presented in the positive control group. Besides, the supernatant was colorless and transparent in all TQB2450 sample groups. Red blood cells naturally sank and were redispersed following adequate oscillation. Hemolysis and coagulation were not observed in all TQB2450 sample groups.

Vascular stimulation test

The vascular stimulation test included the TQB2450 group with 4 male rabbits and 4 females. The highest planned concentration of 10 mg/mL was given by a single injection into the right auricular vein at the volume of 6 mL/kg. In parallel, the same volume of 0.9% sodium chloride injection as the control was administered through injection into the left auricular vein. The day of first dose was the first day of the study.

A single injection of TQB2450 with a concentration of 10 mg/mL at a volume of 6 mL/kg was delivered in the ear vein of Japanese white rabbits, which had no irritation to the blood vessels and surrounding tissues of the injection site.

Comprehensive evaluation

TQB2450 did not significantly change the electrocardiographic parameters, blood pressure, and respiratory rate of male and female monkeys.

The general observation, body mass, food intake, temperature, electrocardiogram, blood pressure, hemanalysis, serum biochemistry, urinalysis, and gross anatomy showed no obvious abnormalities following single intravenous doses of TQB2450 of 200 and 400 mg/kg. The MTD was 400 mg/kg.

TQB2450 was intravenously injected for 4 weeks and suspended for 4 weeks. The NOAEL was 200 mg/kg (AUC0-t was 507909.7enously inhr*μg/mL at this dose after 5 doses). Hemolysis and coagulation were not observed in all TQB2450 groups. TQB2450 had no irritation to the blood vessels and surrounding tissues of the injection site.

1.6 TQB2450 Human tolerance and kinetics and other clinical studies

In the phase I human clinical trial of TQB2450, patients with advanced malignant tumors without standard treatment methods were selected. According to the dose escalation design, six dose of 1mg/kg, 3mg/kg, 10mg/kg, 20mg/kg, 30mg/kg and 1200mg were selected. The drug was given once every 3 weeks until progressed disease or intolerable toxicity.

Until May 2021, a total of 40 patients with advanced malignant tumors were enrolled in the phase I clinical study of TQB2450 injection tolerability and pharmacokinetics (Protocol No. TQB2450-I-01), including 1, 3, 10, 20, 30 mg/kg/q3w and 1200 mg/kg/q3w dose groups. At present, 1 subject is on the group. Preliminary efficacy was observed in Hodgkin lymphoma, lung cancer, cervical cancer, liver cancer, renal cancer, melanoma and other tumors, indicating that the activity of TQB2450 injection is worth looking forward to and exploration and registration studies for multiple indications are being carried out.

Table 4 TQB2450 Injection I Summary of efficacy

| Dose group | Patients number | Efficacy |
| --- | --- | --- |
| 1mg/kg | 1 | Withdraw because of PD |
| 3mg/kg | 6 | Four patients withdraw because of PD and 2 patients were on going. Among them, 1 patient with Hodgkin's lymphoma had the best efficacy of PR, and was discharged from the group after 12 cycles; One patient with liver cancer in the group had the best efficacy of SD in 7 cycles; One lung cancer patient in the group had the best efficacy of SD in 7 cycles. |
| 10mg/kg | 6 | Four patients withdraw because of PD and 2 patients were on going. Two patients with DLBCL and Hodgkin lymphoma, respectively, both had been treated for 15 cycles, and the best efficacy was SD and PR, respectively. |
| 20mg/kg | 3 | All 3 patients were out of the study. One patient with renal cancer was discharged from the study due to SAE after treated for 8 cycles, and the best efficacy was SD. |
| 30mg/kg | 6 | Five patients were out of the study. One patient with renal cancer had been treated for 9 cycles, with the best efficacy of SD. |
| 1200mg | 12 | Five patients were out of the study and 7 patients were on going. One patient with melanoma had been treated for 6 cycles and the first tumor evaluation was SD. One patient with lung cancer had been treated for 6 cycles and the first tumor evaluation was SD. One patient with rectal cancer had been treated for 4 cycles and the first tumor evaluation was SD. |

Safety Results

Six patients were enrolled in the 30mg/kg group, and no DLT was observed. Statistical analysis was conducted on adverse events occurred in 34 patients enrolled in the study, and the summary of adverse events occurred in ≥4 patients were as follows. The safety data were summarized, and adverse events were mainly abnormal in various examinations, with severity of grade 1~2 or consistent with the characteristics of underlying diseases. Therefore, TQB2450 injection showed its good tolerability and safety, and the overall adverse events were controllable, basically consistent with the adverse reactions of similar drugs.

PD/PK Results

At present, the plasma concentrations of the first 6 dose groups were detected. After intravenous injection of HUMIRA, the results of serum drug exposure and Cmax showed good linearity from 3 mg/kg to 30 mg/kg. The half-life of the first cycle was about 320 hours in the 1200 mg dose group. At 21 days of treatment, the vast majority of receptor occupancy was more than 90%, and it was basically determined that the drug could maintain saturation until before the next dose to ensure the continuous exertion of efficacy, which also indicated the feasibility of once 3-week administration.

Dose Recommendations

Taking into account the average body weight of Chinese people, receptor-occupancy rate, probability of immunogenicity, clinical practice convenience, dose of similar drugs, efficacy and safety, 1200mg/ time/q3W is recommended for late-stage trials, and its efficacy and safety have been confirmed in subsequent clinical trials.

1.6.1 Safety and tolerability of TQB2450 in combination with anlotinib

The sponsor has conducted several clinical studies of TQB2450 in combination with Anlotinib. As of July 2021, 229 subjects have been included in multiple phase Ib studies of Anlotinib Hydrochloride Capsules combined with TQB2450 Injection for statistical analysis of adverse events. Adverse events were mainly various test abnormalities, mainly grade 1-2, or consistent with underlying disease characteristics. The incidence of grade 3 and above adverse reactions was 14.29%, mainly including palmar-plantar erythrodysesthesia syndrome, prolonged QT interval in ECG, hypertriglyceridemia, hypertension and diarrhea. The combination of anlotinib hydrochloride and TQB2450 had no additive effect on adverse reactions, was well tolerated, and the overall adverse events were manageable without unexpected adverse events.

Based on the current clinical study data, the adverse reactions of TQB2450 combined with anlotinib were controllable and tolerable. The adverse events were consistent with the safety data of anlotinib and the phase I study of TQB2450, and no unexpected adverse events occurred due to the combination. It is also suggested that the TQB2450 dosing regimen of 1200mg every 3 weeks is well tolerated.

1.7 Preclinical Studies of anlotinib

Pharmacology Studies

Anlotinib is a multi-targeted receptor tyrosine kinase (RTK) inhibitor. The results of the kinase inhibition assay showed that anlotinib inhibited VEGFR1 (IC50: 26.9 nM), VEGFR2 (IC50 Is 0.2 nM), VEGFR3 (IC50: 0.7 nM), c-Kit (IC50: 14.8 nM), PDGFRβ (IC50: 115 nM) of kinase activity.

In vitro assays showed that anlotinib inhibited the proliferation of various tumor cell lines (786-O, A375, A549, Caki-1, U87MG, MDA-MB-231, HT-29, NCI-H526, HMC-1). IC50 was between 3.0 μM and 12.5 μM. It can significantly inhibit the phosphorylation level of VEGFR2 and related downstream proteins in HUVECs cells, the phosphorylation level of c-Kit and related downstream proteins in Mo7e cells, and the phosphorylation level of PDGFR and related downstream proteins in U87MG cells. It can also significantly inhibit the proliferation, migration and tube formation of HUVECs stimulated by VEGF-A. It can inhibit the formation of microvessel-like structures in rat arterial rings.

1.8 Anlotinib Toxicology Studies

**General Toxicology:** SD rats were administered the drug at 0.2, 0.8 and 3.0 mg/kg for 26 weeks; then, drug administration was discontinued for 6 weeks. The no-observed-adverse-effect level (NOAEL) was 0.25 mg/kg, about 0.65 times of the clinical dose (12 mg/person) based on body surface area; significant toxicity occurred at 3.0 mg/kg and the toxicity was targeted to the organs such as were teeth and kidneys. Beagle dogs were administered anlotinib at 0.02, 0.08 and 0.32 mg/kg for 39 weeks and recovered for 6 weeks after the treatment. The NOAEL was < 0.02 mg/kg, about 0.05 times of the clinical dose (12 mg/person) based on body surface area, and the main toxic reactions were arteriolar/arteriolar arteritis and its secondary changes.

**Genotoxicity**: The results of Ames test, chromosomal aberration test in Chinese hamster lung fibroblasts (CHL) and mouse bone marrow micronucleus test were negative.

**Reproductive Toxicity:** The NOAEL for fetal development was <0.3 mg/kg when pregnant SD rats were intragastrically administered with anlotinib.

**Carcinogenicity:** Carcinogenicity studies have not been conducted with anlotinib.

1.9 Study on the Dynamics of Anlotinib

Absorption

Following oral administration of anlotinib 5 mg to 12 healthy subjects in the fasted state, the mean time to peak plasma concentration of anlotinib was 9.3 hours, and the elimination in vivo was slow, with a mean elimination half-life of 113 hours. A high-fat diet reduced the oral bioavailability of amlotinib, and the total in vivo exposure of amlotinib was approximately 80% of that of fasting administration. The effect of a low to medium fat diet on the bioavailability of amlotinib is unknown.

After oral administration of anlotinib 10, 12 and 16 mg to 19 subjects with solid tumors in the fasted state, the mean time to peak plasma concentration of the unchanged drug was about 6 ~ 11 h; the mean elimination half-life was 95 ~ 116 h. Within the dose range of 10 ~ 16 mg, the in vivo exposure level of anlotinib was positively correlated with the administered dose, but the linear relationship was uncertain. There was no significant gender difference. Fifteen subjects with solid tumors were treated with 12 mg once a day for 2 weeks and 1 week off for a dosing cycle. The plasma drug concentration of the original drug in the subjects reached the peak on the 14th day of continuous dosing. The plasma concentration of anlotinib reached 21.1-121 ng/mL on day 14 of the first cycle, and decreased to 5.05-28.5 ng/mL one week after drug withdrawal. The blood concentration of anlotinib reached 22.1 to 101 ng/mL on day 14 of the second cycle. After two cycles of treatment, there was no significant change in plasma drug concentration between the second cycle and the first cycle.

Distribution

After a single fasting oral administration of 12 mg and 16 mg anlotinib hydrochloride capsules in subjects with advanced cancer, the mean apparent volume of distribution ranged from 2061 to 3312L. The plasma protein binding rate of anlotinib was 93% by equilibrium dialysis (in vitro), and there was no concentration dependence in the range of 300~1200 ng/mL.

Metabolism

Anlotinib is primarily metabolized by CYP1A2 and CYP3A4/5, followed by CYP2B6, CYP2C8, CYP2C9, CYP2C19, and CYP2D6; anlotinib is not a substrate of P-glycoprotein.

Excretion

In a 14C-labeled human experiment on the balance of substances of amlotinib, cumulative excretion of amlotinib and its major metabolites via feces and urine was detected to be approximately 62.04% of the administered dose in tumor-bearing subjects after a single oral administration of 12 mg amlotinib for 2648 hours (110 days), with fecal excretion being 48.52% and urinary excretion being 13.52% of of the administered dose.

Special Populations

Pharmacokinetic studies have not been performed in special populations such as those with hepatic or renal insufficiency.

1.10 Safety and tolerability of anlotinib

Twenty-two clinical trials have been conducted for amlotinib, involving 1788 patients with advanced tumors, including non-small cell lung cancer, soft tissue sarcoma, small cell lung cancer, clear cell renal cancer, colorectal cancer, medullary thyroid cancer, esophageal squamous cell cancer, hepatocellular cancer, neuroendocrine tumors, gastric cancer, and bone tumors. Anlotinib were initiated at a dose of 12 mg for 2 weeks and discontinued for 1 week. The adverse events with an incidence of ≥10% included hypertension, fatigue, hand-foot syndrome, hypertriglyceridemia, proteinuria, diarrhea, loss of appetite, increased serum thyroid stimulating hormone, hypercholesterolemia, hypothyroidism, etc.

Summary of adverse reactions

The table below summarizes the adverse reactions occurring in a total of 1788 subjects with advanced tumors from 22 clinical trials of anlotinib. They are presented by system organ and occurrence frequency. Occurrence frequency: very common (≥ 10%), common (1% ~ 10%, including 1%), occasional (0.1% ~ 1%, including 0.1%), rare (0.01% ~ 0.1%, including 0.01%), very rare (< 0.01%). Within each frequency grouping, these adverse events are presented in descending order of severity.

Table 5 Summary of Adverse events of Anlotinib

| **General disorders** | |
| --- | --- |
| Very common | Fatigue, weight decreased |
| Common | Chest pain, fever, influenza-like reaction ^a^, edema ^b^, Cancer pain |
| Occasional | Hypersensitivity reaction ^c^, chills |
| Rare | Poor wound healing ^d^ |
| **Cardiovascular system disorders** | |
| Very common | Hypertension, sinus tachycardia |
| Common | Sinus bradycardia, palpitations, myocardial ischemia, sinus arrhythmia |
| Occasional | Cardiac failure, vena cava thrombosis, atrial fibrillation, pulmonary artery thrombosis, myocardial infarction, venous thrombosis limb, flushing |
| Rare | Hot flushes |
| **Hemorrhage** | |
| Common | Hemoptysis ^e^ Gastrointestinal bleeding ^f^, Other haemorrhage ^g^ |
| **Gastrointestinal disorders** | |
| Very common | Diarrhoea, abdominal pain, oropharyngeal pain, vomiting, nausea, toothache, stomatitis |
| Common | Abdominal distension, constipation, mouth ulceration, dry mouth, oral pain, gastroesophageal obstruction, intestinal obstruction |
| Occasional | Gastritis, pancreatitis, enteritis, melena |
| **Skin and subcutaneous tissue disorders** | |
| Very common | Hand-foot syndrome ^h^ |
| Common | Rash, alopecia, pruritus, skin exfoliation, congestion under finger/toenails, hyperhidrosis |
| Occasional | Skin pain, dermatitis acneiform, pigmentation disorder, dry skin, erythema, pustular rash, seborrheic dermatitis |
| Rare | Eczema, dermatitis bullous, erythema generalised, dermatitis herpetiformis, herpes simplex, blister |
| **Renal and urinary disorders** | |
| Very common | Proteinuria |
| Common | Urinary tract infection |
| **Metabolism and nutrition disorders** | |
| Very common | Hypertriglyceridemia, decreased appetite, hypercholesterolemia, hyperglycemia, hyponatremia, hypoalbuminemia |
| Common | Hypokalemia, hypophosphatemia, hypocalcemia, hyperuricemia, hypomagnesemia |
| **Respiratory, thoracic and mediastinal disorders** | |
| Very common | Dysphonia, cough |
| Common | Dyspnoea, upper respiratory tract infection, epistaxis, lung infection, pneumothorax, pleural effusion |
| Occasional | Interstitial lung disease |
| **Blood and lymphatic system disorders** | |
| Very common | White blood cell count decreased, platelet count decreased, anaemia, neutrophil count decreased |
| Common | Lymphocyte count decreased |
| Rare | Increased eosinophil count |
| **Musculoskeletal and connective tissue disorders** | |
| Very common | Musculoskeletal pain ^i^ |
| Common | Arthralgia |
| **Endocrine disorders** | |
| Very common | Hypothyroidism |
| Common | Hyperthyroidism |
| **Mental and neurological disorders** | |
| Common | Headache, dizziness, insomnia, hypoesthesia |
| Occasional | Paraesthesia, somnolence, vertigo, hypoaesthesia oral, seizure ^j^, dysgeusia |
| **Eye disorders** | |
| Occasional | Blurred vision, dry eye |
| **Hepatobiliary disorders** | |
| Common | Hyperbilirubinemia |
| Occasional | Cholecystitis, jaundice |
| Rare | Hepatic failure |
| **Ear and vagus Go Disease** | |
| Common | Tinnitus |
| **Investigations** | |
| Very common | Blood thyroid stimulating hormone increased, aspartate aminotransferase increased, gamma-glutamyltransferase increased, blood bilirubin increased, alanine aminotransferase increased, electrocardiogram QT prolonged, low density lipoprotein increased, red blood cell urine positive, blood alkaline phosphatase increased, bilirubin conjugated increased, occult blood positive |
| Common | Lipase increased, Amylase increased, Blood creatinine increased, Activated partial thromboplastin time prolonged, Blood urea increased |

a Influenza-like reaction mainly manifested as chills, nasal congestion, runny nose, muscle soreness, fatigue and other symptoms.

b Edema included peripheral edema, facial edema, local edema and generalized edema;

c Hypersensitivity reactions include systemic allergic reactions, upper respiratory tract hypersensitivity reactions, acute allergic reactions, etc.

d clinical trial excluded subjects with non-healing wounds;

e Hemoptysis includes bleeding events of the lung and upper respiratory tract such as hemoptysis and bronchial hemorrhage;

f Gastrointestinal bleeding includes gingival bleeding, oral bleeding, pharyngeal congestion, stomach bleeding, intestinal bleeding, anal bleeding, hemorrhoids bleeding, etc.

g Other bleeding including tumor bleeding, nail bed bleeding, wound bleeding, subcutaneous bleeding, vaginal bleeding, menorrhagia, uterine bleeding, retinal bleeding, conjunctival bleeding and cerebral hemorrhage;

h The term for HFRS in MedDRA is palmoplantar redness and swelling syndrome.

i Musculoskeletal pain included back pain, limb pain, myalgia, musculoskeletal pain, neck pain, bone pain, chest musculoskeletal pain and low back pain.

j Epilepsy includes partial seizures.

Anlotinib Hydrochloride is conducting combination TQB2450 Injection ( PD-L1 ) treatment of advanced soft tissue sarcoma, small cell lung cancer, cholangiocarcinoma, hepatocellular carcinoma, triple-negative breast cancer, breast cancer, melanoma and many others Phase 1b in China, and the safety was controllable and tolerable. Adverse events were consistent with those in the above pooled data. There was no unexpected adverse event due to the combination.

1.11 Study results of anlotinib in treatment of esophageal squamous cell carcinoma

The poster of the 2019 American Society of Clinical Oncology Gastrointestinal Cancer Symposium (ASCO-GI) showed the ALTER1102 trial, a randomized, double-blind, placebo-controlled phase Ⅱ multicenter clinical trial of anlotinib in the treatment of advanced esophageal squamous cell carcinoma after chemotherapy failure. A total of 164 patients who had failed at least one previous line of chemotherapy were randomly assigned in a 2:1 ratio to receive anlotinib alone or placebo. The primary endpoint was progression-free survival (mPFS). mPFS was 3.02 months in the anlotinib group and 1.41 months in the placebo group, HR (95%CI) was 0.46(0.32,0.66), P < 0.0001, achieving the primary endpoint. Anlotinib prolonged mPFS by 1.61 months compared with placebo. The objective response rate (ORR) was also more than doubled in the anlotinib group compared with the placebo group (7.34% vs 3.64%), and the disease control rate (DCR) was more significant (64.22% vs 18.18%). Compared with placebo, anlotinib did not significantly benefit the OS of patients with advanced esophageal squamous cell carcinoma as second-line or beyond. The proportion of patients who continued to receive other treatments after the treatment was not equal between the two groups (41.24% in the anlotinib group VS 72.73% in the placebo group, P=0.0002), which may affect the overall survival to some extent. In terms of safety, the side effects of anlotinib mainly include hypertension and fatigue, which are common in the same class of drugs, and the incidence is low.

The prognosis of patients with advanced ESCC is poor. Paclitaxel + cisplatin is still the standard first-line regimen for patients with advanced ESCC. A phase Ⅱ, multicenter, single-arm, open-label clinical trial of anlotinib combined with paclitaxel and cisplatin in the first-line treatment of patients with advanced esophageal squamous cell carcinoma is currently underway. The patients with unresectable, locally advanced recurrent or metastatic esophageal squamous cell carcinoma are enrolled. The aim of this study is to explore the efficacy and safety of anlotinib combined with cisplatin and paclitaxel as first-line treatment of advanced ESCC. The preliminary results were included in the 2019 and 2021 American Society of Clinical Oncology Gastrointestinal Cancer (ASCO-GI) posters. The study planned to enroll 47 patients, and by March 2021, a total of 51 patients were enrolled,4 patients withdrew informed consent, 1 patient was enrolled in error, 46 patients could be included in ITT analysis,47 were included and full analysis set, and 42 patients had preliminary efficacy evaluation data at the data cutoff date and were included in the per-protocol analysis set. The baseline data of 46 ESCC patients evaluated for efficacy were as follows: median age 65.5 years, range 43-75 years. There were 31 male patients and 15 female patients. There were 3,3 and 40 patients with IIIb, Ⅳa and Ⅳb, respectively. ECOG PS was 0 in 13 cases and 1 in 33 cases. The histological type was all squamous cell carcinoma. In the past, 23 patients received surgical resection and 23 patients did not receive surgical treatment. Forty-three patients had metastasis, and the most common metastases were lymph node metastasis, lung metastasis and liver metastasis. The results showed that the preliminary median PFS was 8.38 months (95%CI: 5.86-10.90) in anlotinib combined with chemotherapy as first-line treatment for advanced ESCC. Complete response (CR) was achieved in 1 patient, partial response (PR) was confirmed in 35 patients, PR was not confirmed in 3 patients, and stable disease (SD) was observed in 7 patients. The preliminary objective response rate (ORR) was 78.3%, and the disease control rate (DCR) was 93.5%. The safety data showed that the adverse reactions during the treatment included bone marrow suppression, gastrointestinal reactions, fatigue, hypertension, constipation, hypokalemia, hyperbilirubinemia, liver toxicity, hemoptysis, pain and tachycardia. Grade 3 or above toxicities included myelosuppression, gastrointestinal reactions, hypertension, fatigue, hypokalemia, hyperbilirubinemia and pain. The incidence of adverse reactions was relatively low, and the overall adverse reactions were safe and controllable.

1.12 Current status of first-line treatment for esophageal squamous cell carcinoma

At present, esophageal squamous cell carcinoma has become one of the ten characteristic tumors determined by the Ministry of Health of China. According to the latest data released by the World Health Organization, in 2018, there were 572,000 new cases of esophageal cancer in the world, ranking seventh in the incidence rate, and 509,000 deaths, ranking sixth, while there were 258,000 new cases of esophageal cancer in China, ranking sixth in the incidence rate, and 193,000 deaths, ranking fourth, both higher than the world average level. In Europe and the United States, adenocarcinoma accounts for more than 50% of the esophageal cancer, while in China, squamous cell carcinoma accounts for more than 90%. There are many controversies in the treatment of esophageal cancer, including the choice of incision, the extent of lymph node dissection, the status of neoadjuvant therapy and adjuvant therapy in the comprehensive treatment of esophageal cancer. The key is that there is still a lack of convincing clinical research in China. Therefore, there are many treatment options, and new technologies and methods emerge in an endless stream.

The 2021 edition of CSCO Guidelines for the Diagnosis and Treatment of Esophageal Cancer points out that the treatment of unresectable locally advanced esophageal cancer is still based on radical concurrent chemoradiotherapy/chemotherapy + radiotherapy/chemotherapy. In the section of first-line treatment of metastatic esophageal cancer, based on the findings of KEYNOTE-590, CHECKMATE-649, and ESCORT, the CSCO updated the 2021 guidelines as following: (1) Pembrolizumab + fluorouracil (5-FU or capecitabine) + cisplatin, (CPS≥10 , level 1A), level Ⅱ expert recommendation; (2) camrelizumab + paclitaxel + cisplatin (recommended for squamous cell carcinoma, level 1A), level Ⅱ expert recommendation; (3) nivolumab + fluorouracil (5-FU or capecitabine) + oxaliplatin (recommended for adenocarcinoma, CPS≥5, level 1A), level Ⅱ expert recommendation; (4) albumin-bound paclitaxel + cisplatin (recommended for squamous cell carcinoma, level 3A), level Ⅲ expert recommendation; (5) camrelizumab + apatinib + paclitaxel liposome + nedaplatin (recommended for squamous cell carcinoma, level 3A), level Ⅲ expert recommendation.

The KEYNOTE-590 is a global, multicenter, randomized, controlled, double-blind, phase III clinical trial exploring pembrolizumab combined with chemotherapy as the first-line treatment for esophageal cancer. A total of 749 patients with unresectable locally advanced or metastatic esophageal cancer were enrolled, including 548 patients with esophageal squamous cell carcinoma. The proportion of patients with squamous cell carcinoma was about 72.9%-73.5%, and the proportion of PD-L1 CPS≥10 was about 49.9%-52.4%. The results showed that in the overall population, the survival time of the pembrolizumab-chemotherapy group was significantly better than that of the chemotherapy group (median survival mOS: 12.4 months vs 9.8 months; P < 0.0001; HR: 0.73; 95%CI: 0.62-0.86). Further analysis showed that in patients with CPS≥10, pembrolizumab-chemotherapy had a significant survival advantage (13.5 months vs 9.4 months; P < 0.0001; HR: 0.62; 95%CI: 0.49-0.78). In ESCC subgroup, the survival advantage of pembrolizumab-chemotherapy group was also significant (12.6 months vs 9.8 months; P =0.0006; HR: 0.72; 95%CI: 0.60-0.88). In terms of progression-free survival (PFS), the overall population (6.3 months vs 5.8 months, HR=0.65, 95%CI 0.55-0.76; P<0.0001), PD-L1 CPS≥10 (7.5 months vs 5.5 months, HR=0.51, 95%CI 0.41-0.65; P<0.0001) and esophageal squamous cell carcinoma (6.3 months vs 5.8 months, HR=0.65, 95%CI 0.54-0.78; P<0.0001) achieved the study endpoint of significantly superior PFS with pembrolizumab-chemotherapy in all three populations. Based on this, the current CSCO guidelines include pembrolizumab combined with fluorouracil (5-FU or capecitabine) and cisplatin as a level II recommendation for the first-line treatment of advanced esophageal cancer patients with PD-L1≥10%.

The recommendation for camrelizumab in combination with chemotherapy was based on the results of the multicenter, randomized, double-blind, phase III study evaluating the efficacy and safety of camrelizumab in combination with paclitaxel and cisplatin in the first-line treatment of advanced esophageal cancer (ESCORT-1st). ESCORT-1st study enrolled 596 subjects from more than 60 centers in China and assigned randomly in a 1:1 ratio to receive camrelizumab + paclitaxel + cisplatin or placebo + paclitaxel + cisplatin for no more than six cycles, followed by maintenance therapy with camrelizumab or placebo until disease progression, unacceptable toxicity, or other circumstances requiring discontinuation of treatment. The maximum duration of camrelizumab or placebo was 2 years. The primary study endpoints were progression-free survival (PFS) and overall survival (OS) as assessed by an independent review committee (IRC) according to RECIST v1.1 criteria. The results showed that camrelizumab combined with paclitaxel and cisplatin significantly prolonged progression-free survival (6.9 months vs5.6 months) and overall survival (15.3 months vs12.0 months) in patients with advanced esophageal cancer, compared with the standard first-line treatment of paclitaxel and cisplatin. The Center for Drug Evaluation (CDE) of the China Food and Drug Administration has also accepted the indication of camrelizumab for the first-line treatment of advanced esophageal cancer.

Camrelizumab plus apatinib and chemotherapy is recommended in new guidelines based on results of a single-arm, prospective, phase II study evaluating camrelizumab plus apatinib and chemotherapy as first-line treatment for advanced esophageal squamous cell carcinoma. From August 2018 to February 2019, a total of 30 patients with unresectable locally advanced or recurrent/metastatic esophageal squamous cell carcinoma were enrolled in this study. The patients were treated with camrelizumab + apatinib + paclitaxel liposome + nedaplatin for no more than 9 cycles, followed by maintenance therapy with camrelizumab + apatinib until disease progression, unacceptable toxicity, or other circumstances requiring discontinuation of treatment. The primary endpoint was objective response rate (ORR). The ORR was 80.0%, and the median response time was 9.77 months. The disease control rate (DCR) was 96.7%. The median progression-free survival (PFS) was 6.85 months, and the median overall survival (OS) was 19.43 months. In terms of safety, the most common grade 3-4 treatment-related adverse events (AEs) were leukopenia (60.0%), neutropenia (83.3%), and elevated aspartate aminotransferase (AST) level (26.7%). No treatment-related death occurred. This study confirmed that the four-drug combination has good antitumor activity and manageable safety in the first-line treatment of advanced esophageal squamous cell carcinoma.

In conclusion, this study aimed to investigate the efficacy and safety of paclitaxel + cisplatin +TQB2450 injection with or without anlotinib in the first-line treatment of patients with advanced esophageal squamous cell carcinoma, and was carried out according to this protocol after the review and approval of the hospital ethics committee.

2. Study objectives

Primary objective: To evaluate the efficacy of paclitaxel + cisplatin + TQB2450 with or without anlotinib as first-line treatment in patients with advanced esophageal squamous cell carcinoma;

Secondary Objective: To evaluate the safety of paclitaxel + cisplatin + TQB2450 injection with or without anlotinib as first-line treatment in patients with advanced esophageal squamous cell carcinoma;

Exploratory Objectives: To explore the correlation between biomarkers and clinical efficacy.

3. Study plan

3.1 Study Design

Multi-center, open-label Dual Arm test design.

Each study site selected appropriate patients and conducted the clinical trial in a competitive manner.

3.2 Study Duration

Cohort 1: Subjects will receive initial treatment with TQB2450 + anlotinib + paclitaxel + cisplatin in 3-week cycles. Patients without disease progression (CR+PR+SD) will receive TQB2450 + Anlotinib as maintenance treatment after initial treatment (4-6 cycles, 6 cycles treatment are assigned to patients whenever possible at the investigators’ discretion based on patient tolerance) until PD or intolerable toxicity. Efficacy is evaluated every two cycles during initial treatment and every three cycles during maintenance treatment.

Cohort 2: Subjects will receive initial treatment with TQB2450 + paclitaxel + cisplatin in 3-week cycles. Patients without disease progression (CR+PR+SD) will receive TQB2450 as maintenance treatment after initial treatment (4-6 cycles, 6 cycles treatment are assigned to patients whenever possible at the investigators’ discretion based on patient tolerance) until PD or intolerable toxicity. Efficacy is evaluated every two cycles during initial treatment and every three cycles during maintenance treatment.

3.3 Study Population

Patients with measurable disease who meet the criteria for first-line treatment of advanced esophageal squamous cell carcinoma, Patients must sign an informed consent prior to enrollment.

3.3.1 Inclusion criteria

Subjects can participate in the study only if all the following criteria are met:

1. Histopathologically confirmed, unresectable, locally advanced, recurrent, or metastatic esophageal squamous cell carcinoma (ESCC) (excluding mixed adenosquamous carcinoma);
2. No prior systemic therapy or have tumour recurrence more than 6 months after the completion of (neo) adjuvant or radical therapy (including radical surgery and radical chemoradiotherapy;

Note: Patients with advanced or recurrent non-target lesions who progressed again after radiotherapy alone were included. The time from the end of palliative treatment for local lesions (non-target lesions) to enrollment was more than 2 weeks.

1. At least one measurable lesion according to Response Evaluation Criteria in Solid Tumors version 1.1 (RECIST v1.1); Measurable lesions should not have received local treatment such as radiotherapy (lesions located in the area of previous radiotherapy can also be selected as target lesions if they are confirmed to have progressed and meet RECIST v1.1 criteria);
2. Age 18-75 years;
3. Eastern Cooperative Oncology Group-Performance status (ECOG-PS) of 0-1; Predicted life expectancy of ≥3 months;
4. Adequate function of the important organs as evidenced by the following:
5. Hemanalysis:

- hemoglobin (Hb) ≥90g/L (no blood transfusion within 28 days);
- absolute neutrophil count (ANC) ≥1.5×10^9^/L;
- platelets (PLT) ≥100×10^9^/L.

1. Biochemistry:

- total bilirubin (TBIL) ≤1.5×upper limit of normal (ULN)；
- alanine aminotransferase (ALT) and aspartate aminotransferase (AST) ≤2.5×ULN; ALT and AST ≤5×ULN in patients with liver metastases;
- Creatinine (Cr) ≤1.5×ULN and creatinine clearance rate (CCr) ≥60 mL/min (Cockcroft-Gault formula).

1. Adequate coagulation function: international normalization ratio (INR), or prothrombin time (PT) ≤1.5×ULN;
2. Women of reproductive age were required to use appropriate contraception from the time of screening until 3 months after discontinuation of study treatment and were not breast-feeding. A negative pregnancy test or one of the following criteria before the initiation of dosing proved that there was no risk of pregnancy:

a. Postmenopausal status was defined as age ≥50 years and amenorrhea for at least 12 months after discontinuation of all exogenous hormone replacement therapy;

b. Women aged <50 years were also considered postmenopausal if they had amenorrhea for 12 months or more after discontinuation of all exogenous hormone therapy and the luteinizing hormone (LH) and follicle-stimulating hormone (FSH) were accorded with the laboratory normal reference range;

c. Patients who had undergone irreversible sterilization procedures, including hysterectomy, bilateral oophorectomy, or bilateral salpingectomy, with the exception of those who had bilateral tubal ligation.

For men, consent is given to use an appropriate method of contraception or to have been surgically sterilized during the trial period and 8 weeks after the last drug administration.

1. Be willing and able to provide written informed consent for the trial, and have better compliance with follow-up.

3.3.2 Exclusion criteria

Subjects who meet any of the following criteria are not eligible to enter the study:

1. Patients with ESCC who have complete obstruction under endoscopic guidance and need interventional therapy to relieve obstruction;
2. Patients with ulcerative ESCC;

Note: This refers primarily to patients with ulcers adjacent to blood vessels that increase the risk of bleeding.

1. Patients after esophageal or tracheal stent placement;
2. Patients with a high risk of bleeding or perforation due to tumor invasion of adjacent organs (large arteries or trachea) of the esophageal lesion, or with established fistulas;
3. Patients who had hematemesis, bloody stool and daily blood loss ≥2.5 mL or any CTCAE grade ≥3 bleeding events within 3 months before screening, or who had any evidence of bleeding, regardless of severity, or whose history was judged by the investigator to be ineligible for enrollment;
4. Patients who have allergic reactions to drug formulations or excipient components or similar drugs;
5. Patients who had received adjuvant chemotherapy with paclitaxel and had recurrence or metastasis within one year;

Note: Patients with recurrence or metastasis for more than one year could be included in the study.

1. Factors significantly affecting oral medication (e.g. swallowing difficulty, chronic diarrhea, and intestinal obstruction);
2. The liver metastatic burden with accounting for approximately more than 50% of the total liver volume;
3. Patients with any severe and/or uncontrolled illness, including:

- Patients with poor blood pressure control using antihypertensive drugs (systolic blood pressure ≥150 mmHg or diastolic blood pressure ≥100 mmHg); patients with grade II or above myocardial ischemia or myocardial infarction, arrhythmia (including QT interval ≥480ms); patients with Grade III-IV cardiac insufficiency, or with left ventricular ejection fraction (LVEF) <50% via the cardiac color ultrasound;
- Active or uncontrolled severe infection;
- Liver diseases such as cirrhosis, decompensated liver disease, chronic active hepatitis;
- Poor diabetes control (fasting blood glucose [FBG] >10 mmol/L);
- Urinary protein ≥ ++, and confirmed 24-hour urinary protein >1.0 g;

1. Unhealed wound or fracture for a long time;
2. Patients with ESCC who have active bleeding of the primary lesion within 2 months; NCI CTCAE grade >1 pulmonary hemorrhage within 4 weeks prior to enrollment; NCI CTCAE grade >2 other site bleeding within 4 weeks prior to enrollment; patients with bleeding tendencies (e.g., active gastrointestinal ulcers) or those receiving thrombolytic or anticoagulation therapy such as warfarin, heparin, or similar agents;
3. Patients who have undergone major surgical procedures (e.g., craniotomy, thoracotomy, or laparotomy) within 4 weeks before the first study dose or are anticipated to require major surgery during the study treatment;
4. Patients with a history of gastrointestinal perforation and/or fistula within 6 months prior to enrollment, or with thromboembolic events such as cerebrovascular accidents (including transient ischemic attacks), deep vein thrombosis, and pulmonary embolism;
5. Known presence of symptomatic central nervous system metastases and/or carcinomatous meningitis;
6. Clinically significant ascites, including ascites detectable on physical examination, ascites that have been treated previously or currently require treatment, and minimal ascites evident by imaging only but without symptoms;
7. Patients with moderate bilateral pleural effusion, or significant pleural effusion on one side, or those who have developed respiratory impairment requiring drainage;
8. Known active pulmonary tuberculosis;
9. Interstitial lung disease requiring steroid hormone therapy;
10. Uncontrolled metabolic disturbances or other non-malignant or systemic disease or secondary reaction to cancer, which can lead to higher medical risks and/or uncertainty of survival evaluation;
11. Patients with significant malnutrition;
12. Patients with a history of psychotropic substance abuse and difficulty achieving abstinence or with psychiatric disorders;
13. Patients with a history of immunodeficiency, including those with a positive HIV test or suffering from other acquired or congenital immunodeficiency diseases, or those with a history of organ transplantation;
14. History of other primary malignant tumors, except for the following: 1) Complete remission of malignant tumors for at least 2 years prior to enrollment, without further treatment during the study; 2) Adequately treated non-melanoma skin cancer or lentigo maligna with no evidence of disease recurrence; 3) Adequately treated carcinoma in situ with no evidence of disease recurrence;
15. Pregnant or lactating women;
16. > Grade 1 Unresolved toxicity according CTCAE due to any previous treatment, excluding alopecia;
17. Patients who have received first-line chemotherapy for advanced disease or radiotherapy prior to first dose;

Note: Patients who previously received local radiotherapy can be eligible if: the end of radiotherapy is more than 3 weeks from the start of study treatment; the target lesion selected for this study is not within the radiation field; or the target lesion is located within the radiation field, but progression has been confirmed; without chemotherapy, immunotherapy and targeted therapy during radiotherapy.

1. Received treatment with Chinese patent medicines with anti-tumor indications as specified in the approved NMP-approved drug instructions (including Fufangbanmao Capsules, Kangai Injection, Kanglaite Capsules / Injection, Aidi Injection, Brucea javanica oil Injection/Capsules, Xiaoaiping Tablets/Injection, Huachansu Capsules, etc.) within 2 weeks prior to the first administration of the medication.
2. Patients previously treated with VEGFR small molecule inhibitors, such as anlotinib, apatinib, lenvatinib, sorafenib, sunitinib, regorafenib and furazolidone;
3. Patients previously received treatment with anti-PD-1 or anti-PD-L1/PD-L2 inhibitors or other therapies acting on T cell costimulatory targets or checkpoints;
4. History of live attenuated vaccination 28 days prior to first dose or planned live attenuated vaccination during the study;
5. Active autoimmune disease requiring systemic therapy (eg, disease-modifying drugs, corticosteroids, or immunosuppressants) within 2 years prior to first dose. Replacement therapy (e.g., thyroxine, insulin, or physiological corticosteroids for adrenal or pituitary insufficiency, etc.) is not considered as systemic therapy;
6. Diagnosis of immunodeficiency or ongoing systemic glucocorticoid therapy or any other form of immunosuppressive therapy (>10 mg/ Prednisone or other effective hormone), and continued use within 2 weeks prior to the start of study treatment;
7. Participated in other clinical trials of anti-tumor drugs within 4 weeks prior to first dose (the washout period is calculated from the end time of the last treatment);
8. Patients who have concomitant diseases that seriously jeopardize the patient's safety or affect the patient's completion of the study, or are considered unsuitable for enrollment for other reasons according to the judgment of the investigator.

3.3.3 Withdrawal Criteria

Subjects may voluntarily withdraw from the trial at any time, or may be withdrawn by the investigator for safety or behavioral reasons, or who are not able to comply with the protocol-required study visit times or steps at the study center where they are enrolled may choose to terminate study treatment and remain in the study for efficacy assessment, safety, and survival follow-up according to the assessment schedule.

Reasons for withdrawal of a subject from the study include:

1) The patients demonstrate poor compliance, regularly failing to adhere to the prescribed medication regimen (patients do not take medication at the planned time and dose more than two times for no reason, and the investigator's judgment will seriously affect the following study and study results);

2) Treatment with other systemic antineoplastic agents (e.g., chemotherapy, hormone therapy, targeted therapy, or biologic agents) that affect the judgment of efficacy;

3) Patients who experienced serious adverse events (SAEs) and were not suitable for further study according to the investigator’s judgment or who had an unintended pregnancy;

4) Patients who are unwilling to continue the clinical trial and insist on withdrawing;

5) The investigators deemed it necessary to stop the study.

If the subject did not return for a scheduled visit and cannot be contacted for at least 3 times, it will be considered as lost to follow-up. Contact information includes: telephone, SMS, social media tools and mail, etc. All such attempts to contact the subject should be documented in the subject's source documents. If it is determined that a subject has died, the study site will make every effort to obtain death information and cause of death, such as using public resources, such as community health registries and databases. If the subject's status is not available despite all attempts, the investigator should report the date the subject was last known to be alive and record in the subject's medical history.

3.3.4 Exclusion criteria

1. Patients who receive chemotherapy, surgery or investigational drug beyond the protocol during the trial;
2. Patients who failed to meet the inclusion criteria were mistakenly included;
3. Patients without medication;

Note: Patients who met criteria 1-2 were included in the safety analysis.

3.3.5 Termination Criteria

Subjects must be terminated from the study if they meet any of the following criteria:

1) Intolerance during treatment and ineligibility for continueding treatment;

2) Disease progression;

3) Other reasons that the investigator considers inappropriate for continuation of study treatment;

4) Termination by investigators.

3.3.6 Withdrawal or treatment termination procedures

Subjects who terminate treatment must continue to complete the remaining visits as required by the protocol, unless the subject withdraws consent. If a subject withdraws from the study or terminates treatment, include but are not limited to the following measures (refer to Section 6: Subject Visit Schedule):

1. The reason must be recorded in the original data;
2. End of treatment (EOT) visits should be performed.;
3. Safety visit.

3.4 Prohibited concomitant medications

During the trial, the use of drugs with anti-tumor indications approved by NMPA, including modern traditional Chinese medicine preparations and immunomodulators (including compound cantharidin capsules, Kangai injection, Kanglaite capsules / Injection, Aidi injection, Brucea javanica oil injection / Capsules, cancer- eliminating copies / Injections, cinobufotalin capsules, etc.) are prohibited.

During the trial, patients are prohibited from receiving any localized lesion-specific treatments including surgery, radiotherapy (except for palliative localized treatments described in 3.6), other systemic antitumor therapies such as chemotherapy, targeted therapies, hormonal therapies, immunotherapy, etc., and hormonal contraceptives are not allowed.

Patients should not receive live vaccines from 28 days before the first dosing of the study treatment to 60 days after the last dosing, including measles, mumps, rubella, chickenpox, yellow fever, seasonal influenza, H1N1 influenza, rabies, Bacille Calmette-Guerin vaccine (BCG), and typhoid vaccines.

3.5 Concomitant medications allowing with caution

- Medications /Food that affect Anlotinib Hydrochloride Capsules
- Use anticoagulant or thromboprophylaxis with caution

Anticoagulant or thrombosis-preventing drugs should be used with caution during treatment to avoid increasing the potential risk of bleeding. It mainly includes but is not limited to the following categories of drugs: salicylic acid derivatives: such as aspirin; heparin anticoagulants: such as low-molecular-weight heparin, enoxaparin, tenoxaparin, adecaparin, etc.; prophylactic anticoagulant drugs after cardiovascular and cerebrovascular events: such as clopidogrel, teagrelor, etc.

- Drugs that interfere with liver p450 enzymes

Inducers (catamizine, rifampicin, and phenobarbital) and inhibitors (ketoconazole, itraconazole, erythromycin, and clarithromycin) of CYP3A, substrates (simvastatin, cyclosporine, and pimozide) of CYP3A4, and other drugs metabolized by CYP3A4 (e.g., benzodiazepines, dihydropyridine calcium antagonists (calcium antagonists may be selected as appropriate for hypertension that cannot be controlled by ACEIs) and HMG-COA reductase inhibitors should be used with caution during treatment. Substrates of CYP2C9 (diclofenac, phenytoin, pyridoxicam, S-warfarin, and tolbutamide) and substrates of CYP2C19 (diazepam, promazine, lansoprazole, and S-mephenytoin) should be used with caution.

- Drugs that prolong the qt interval of the heart

Because drugs have the toxic and side effects of prolonging QT interval in clinical practice, it is required to use drugs that prolong QT interval with caution during the study. It mainly includes but is not limited to the following types of drugs:

Antibacterials (clarithromycin, streptomycin, erythromycin, roxithromycin, metronidazole, moxifloxacin);

Antiarrhythmics (quinidine, sotalol, amiodarone, propylamine, procainamide);

Antipsychotics (rifampin, fluphenazine, droperidol, haloperidol, thioridazine, pimozide, olanzapine, chlorzapine);

Antifungals (fluconazole, ketoconazole);

Antimalarials (mefloquine, chloroquine);

Antidepressants (amitriptyline, promizine, clomipramine, dosulepin, doxepin).

Citrus, star fruit, grapefruit and grapefruit sap can affect cytochrome P450 activity and should be avoided in combination.

- Drugs that impact TQB2450

TQB2450 is an anti-PD-L1 inhibitor, belonging to cancer immunotherapy drugs, so it is not recommended to use hormones and/or drugs or treatments with immunomodulatory function, so as not to affect the efficacy of TQB2450. These agents or treatments may be selected with caution if deemed necessary by the investigator (e.g., topical steroids for rash relief).

3.6 Drugs and therapies that can be used concomitantly in the study

Patients may receive supportive care. Supportive care may be combined with the following drugs or related treatments: antibiotics, analgesics, fluids, psychotherapy, palliative surgery, or any other symptomatic treatment necessary to provide optimal supportive care. Other investigational antineoplastic agents or antitumor chemo/endocrine/immunotherapy are outside the defined supportive care.

Non-routine treatments (eg, herbal remedies or acupuncture) and vitamin/mineral supplements are permitted if there is no effect on study endpoints in the opinion of the investigator. Patients may receive bisphosphonates for treatment of bone metastases during treatment.

Palliative, small-area (< 5% marrow area for radiation) radiation therapy is permitted for bone metastases that are not effectively controlled by systemic therapy or local analgesia, provided that the target lesion does not include a radiation field.

Granulocyte-colony stimulating factor (G-CSF) and other hematopoietic growth factors may be used during treatment if clinical manifestations suggest acute toxic effects such as neutropenic fever require treatment or at the discretion of the investigator. Patients are allowed to use erythropoietin chronically.

When treating with TQB2450 Injection, in order to prevent infusion reactions, H1 blockers (diphenhydramine 50 mg) can be used within 30 – 60 min before each infusion of TQB2450 Injection Deep intramuscular injection, or equivalent) and acetaminophen (500 to 650 mg orally or intramuscular injection). Systemic corticosteroids required for management of infusion reactions or immune-related adverse events must be tapered for at least 2 weeks before the next dose of study drug without suppressing immune system function (≤ 10 mg/day prednisone or equivalent).

In the course of trial observation, all the concomitant medications other than the study drug should be recorded in the patient's medical record and ECRF Medium. The recording time and requirements of concomitant medication in this trial are as follows:

| Time period | RECORD REQUIREMENTS |
| --- | --- |
| 28 days prior to the first administration until first dose | All medications and significant non-drug therapies are recorded, including: generic drug name and daily dose; reason for drug therapy; start and end dates of drug therapy or whether drug was continued at study entry. |
| From the first dose to withdrawal from the trial | All drug treatments and significant non-drug treatments are recorded. |
| Withdrawn from trial until 28 days after the last dose or initiation of other antineoplastic therapy (whichever occurs first) | Recorded only with AE Dispose of all appropriate medical therapy. |

4. Study drug

4.1 Study Drug

TQB2450 Injection: produced and provided by Chia Tai Tianqing Pharmaceutical Group Co., Ltd.

Strength: 600 mg/20ml;

Batch number, packaging, shelf life and storage conditions are based on CoA/package insert and packaging.

Anlotinib Hydrochloride Capsules: manufactured and provided by Chia Tai Tianqing Pharmaceutical Group Co., Ltd.

Strength: 10 mg/capsule, 8 mg/capsule;

Storage condition: Sealed, protected from light, stored below 25℃.

According to the requirements of GCP, the study drug will be kept, distributed and recycled by the hospital.

Distribute and recycle the drugs according to the cycle. Complete records are required for both distribution and recycle. The recycled drugs will be submitted to the sponsor after completing the trial. The monitor regularly checks the use and recording of drugs and monitors the recovery at any time.

4.2 Dosing Regimen

Group 1: Subjects will receive initial treatment with TQB2450 + anlotinib + paclitaxel + cisplatin in 3-week cycles. Patients without disease progression (CR+PR+SD) will receive TQB2450 + Anlotinib as maintenance treatment after initial treatment (4-6 cycles, 6 cycles treatment are assigned to patients whenever possible at the investigators’ discretion based on patient tolerance) until PD or intolerable toxicity. Efficacy is evaluated every two cycles during initial treatment and every three cycles during maintenance treatment.

Group 2: Subjects will receive initial treatment with TQB2450 + paclitaxel + cisplatin in 3-week cycles. Patients without disease progression (CR+PR+SD) will receive TQB2450 as maintenance treatment after initial treatment (4-6 cycles, 6 cycles treatment are assigned to patients whenever possible at the investigators’ discretion based on patient tolerance) until PD or intolerable toxicity. Efficacy is evaluated every two cycles during initial treatment and every three cycles during maintenance treatment.

Table 6 Dosing Regimen

|  | Drug | Dose/Dosage | Dose Frequency | Administration method | Course/Treatment Cycle | Notes |
| --- | --- | --- | --- | --- | --- | --- |
| Initial treatment | TQB2450 Injection | 1200 mg/time | Q3W | Intravenous drip | Every 21 days, administrate on d1 | H1 blockers (diphenhydramine 50 mg deep intramuscular injection, or equivalent) and acetaminophen (500 to 650 mg orally or Intramuscular injection) coule be used for prevention of infusion reactions |
|  | Paclitaxel | 135 mg/m ^2^ | Q3W | Intravenous drip | Every 21 days, administrate on d1 | Hormone plus H1 and H2 receptor antagonists prior to dosing for pretreatment (dexamethasone, diphenhydramine, cimetidine, etc.) |
|  | Cisplatin | 60-75 mg/m ^2^ | Q3W | Intravenous drip | Every 21 days, divided into d1-d3 administration | after paclitaxel |
|  | Anlotinib  (Group 1 only) | 10 mg | 1 capsule daily for 2 weeks | Oral | Every 21 days, administrate on d1-d14 | Fasting before breakfast |
| Maintenance treatment | TQB2450 Injection | 1200 mg/time | Q3W | Intravenous drip | Every 21 days, administrate on d1 | H1 blockers (diphenhydramine 50 mg deep intramuscular injection, or equivalent) and acetaminophen (500 to 650 mg orally or Intramuscular injection) coule be used for prevention of infusion reactions |
|  | Anlotinib  (Group 1 Only) | 10 mg | 1 capsule daily for 2 weeks | Oral | Every 21 days, administrate d1-d14 | Fasting before breakfast |

Note: If the dose of Anlotinib is missed, it should not be taken if less than 12 hours after the next dose. Recommended body surface area calculation formula: 1. body surface area (m ^2^) = 0.0061 × height (cm) + 0.0128 × weight (kg) – 0.1529; 2. Body surface area = (height + weight-60)/100, The actual quantity is allowed to be within ± 10% of the calculated quantity.

- TQB2450 Administration method of injection:

1200 mg TQB2450 is diluted to 250mL with normal saline and infused over a period of 60 ±10 min. The infusion time is from the beginning of TQB2450 infusion to the end of infusion and flushing the tube with normal saline (20mL recommended). The drug will be administered every 21 days.

Observe the injection for particulate matter and discoloration before dilution. Do not use it if particulate matter or discoloration is confirmed.

Discard normal saline equal to the volume of TQB2450 injection that should be drawn from the 250 mL infusion bag containing 0.9% sodium chloride solution. Add the corresponding volume of TQB2450 injection to the intravenous (IV) infusion bags. Gently invert the solution without shaking to ensure adequate mixing.

The entire contents of the IV bag are intravenously delivered via a 0.22 μm in-line filter for 60±10 minutes. Flush immediately after the infusion. The start time and end time of medication will be recorded. If an infusion reaction occurs, appropriate intervention measures should be taken according to the protocol.

Notes: The volume of fluid is extracted in mL for infusion configuration. Different batches of drugs should not be mixed in a single infusion. Ensure that the TQB2450 infusion is transparent, without turbidity and precipitation. TQB2450 does not contain preservatives and IV bags containing TQB2450 must be used immediately after dilution/mixing. If the TQB2450 dilution cannot be used immediately, the method of storage is as follows:

The total storage time from opening the TQB2450 vial to the end of injection should not exceed 6 hours at room temperature;

The total storage time should not exceed 24 hours in the refrigerator of 2 ℃-8 ℃ (36 °F-46 °F) without freezing and shaking;

Mixing with other medications and intravenous injections should be avoided

4.3 Dose Delays and Adjustments

- General principle
- The severity of AEs will be graded according to the National Cancer Institute Common Terminology Criteria for Adverse Events (NCI-CTCAE) v5.0 grading system. Given the trial of the drug combination, the study drugs unrelated to AEs can be identified and the medication regimen can be maintained.
- When severe toxicity of different severity levels occurs simultaneously, adjustments should be performed according to the highest level observed;
- When toxic reactions occur, the investigators may refer to the following rules for delay or dose adjustment. Reasons for dose adjustments or delays, the supportive measures taken, and the outcomes will be documented in the patient's chart and recorded on the eCRF.
- If the medication adjustment was different from the suggested adjustment rules determined by the investigators, the investigators made the corresponding adjustment according to the clinical practice guidelines or previous clinical experience after consultation with the team leader unit, and the relevant reasons were recorded in the patient's medical records.
- If subjects require permanent discontinuation due to toxic reactions caused by anlotinib, TQB2450 and other drugs should be continued until disease progression or unacceptable toxicity (whichever occurs first);
- If subjects require permanent discontinuation due to immune-related toxicity of TQB2450, anlotinib and other drugs should be continued until disease progression or unacceptable toxicity (whichever occurs first).

4.3.1 Dose Delay and Dose Modification of TQB2450 Injection

If the medication is delayed due to AEs caused by TQB2450 injection and cannot be resumed over 12 weeks, TQB2450 injection treatment should be permanently terminated. The management of AEs induced by TQB2450 injection refers to Guidelines of Chinese Society of Clinical Oncology (CSCO) Management of Immune Checkpoint Inhibitor-related Toxicity, 2021 Edition. TQB2450 dose delays are allowed, but no dose adjustment.

4.3.2 Dose Modification of Anlotinib Hydrochloride Capsules

During the treatment, the investigator will decide whether to adjust the dose according to the degree of drug-related toxicity (graded according to NCI CTCAE 5.0) and possible efficacy benefit.

When anlotinib-related AEs occur during the trial, the dosages of anlotinib are allowed to be reduced (10 mg→8 mg in turn). If the dose of 8 mg is not tolerated, then treatment should be discontinued or terminated at the investigator's dicision. If the investigator considers that there is a possibility of disease progression after a period of treatment with stable safety, the dose may be increased once. Each patient can be escalated once (8mg to 10mg).

Recommended dose delays and/or dose reduction for toxicities related to drug are detailed in the table below. In case of non-bleeding adverse reactions, refer to Table 8; when bleeding adverse reactions occur, refer to Table 9.

Table 7 Dose Level of Anlotinib

| Dose Levels | Usage | Number of specific medications |
| --- | --- | --- |
| 1 | 10 mg Oral, once daily | 10 mg Anlotinib hydrochloride capsules, 1 pill Granule |
| 2 | 8 mg Oral, once daily | 8 mg Anlotinib hydrochloride capsules, 1 pill Granule |

Table 8 General principles of dose adjustment according to the grade of AEs

| Grades of AEs  (NCI-CTCAE v5.0) | Time of administration | Dose adjustment |
| --- | --- | --- |
| Grade 3 | Dose delay until recovery to grade 2 | Continue administration after a reduced dose level; if the toxicity is not recovered after 8 weeks, the treatment should be terminated permanently. |
| Grade 4 | Dose delay until recovery to <grade 2 | Continue administration after a reduced dose level; if the toxicity is not recovered after 8 weeks, treatment should be terminated permanently; the investigator can stop the treatment permanently judging by the treatment. |

Table 9 Principles for dose adjustment in case of bleeding AEs

| Bleeding AEs * | Dose adjustment |
| --- | --- |
| Grade 2 | Suspended administration and active symptomatic treatment; restore to <grade 2 within 8 weeks, reduce a dose level; if necessary, treatment should be terminated permanently. |
| ≥Grade 3 | Permanent termination of treatment and emergency medical intervention. |

* Bleeding AEs include hemoptysis, gastrointestinal bleeding, nosebleed, bronchial bleeding, gingival bleeding, gross hematuria, fecal occult blood, and cerebral hemorrhage.

4.3.3 Dose Modification for Paclitaxel and Cisplatin

For paclitaxel or cisplatin, dose modification and delay are allowed, with a maximum delay of 9 weeks (3 cycles) since the last dose, otherwise treatment with the chemotherapeutic agent is discontinued.

During the treatment period, if grade III or above drug-related adverse reactions occur, the dose of the original drug needs to be reduced by 20%, up to two reductions are allowed. If the dose of paclitaxel or cisplatin has been reduced twice, and the dose needs to be reduced for a third time due to toxic reactions, the treatment of the chemotherapy drug must be terminated.

If paclitaxel - or cisplatin-related toxicity was not restored to standard use, the agent causing the toxicity was withheld, and the other chemotherapy agent was administered as planned. If paclitaxel or cisplatin must be permanently discontinued, the remaining chemotherapy agents should be continued according to the schedule.

For other medication requirements, please refer to the latest version of the drug instructions on the market. If the investigator decides that the adjustment of medication is different from the suggested adjustment rule in the course of clinical operation, taking into account the benefit/risk ratio of the subjects, the investigator will make the corresponding adjustment according to the clinical practice guidelines or previous clinical experience after consultation with the team leader. The relevant reasons are recorded in the patient's medical records.

5. Collection of Biological Samples

Participation in this study required the provision of tumor tissue specimens for biomarker analysis, such as PD-L1 expression, TMB testing, MSI testing, and pathological review.

For biomarker analysis, fresh biopsy samples within 3 months before enrollment are preferred. For fresh tissue samples, one or more percutaneous punctures are performed. If fresh biopsy tissue samples could not be obtained, archival tissue samples could be collected and used. Fifteen unstained pathological tissue sections with a thickness of 4-6 μm are taken, and freshly prepared white slides are cut. Detailed requirements are provided in the SOP provided by the central laboratory.

Patients who could not provide tumor tissue samples due to limited tissue samples are also eligible for this study.

6. Study process

6.1 Before study initiation

Assessments within 4 weekss before study initiation:

- Signing the informed consent form;
- Collect medical history and basic data, including patient ID card, gender, age, mailing address and contact number;
- Ask tumor history, other medical history and previous treatment history in detail;
- Imaging studies (i.e. CT/MRI);
- Hepatitis B, hepatitis C, HIV inspection of relevant indicators;
- Collection of archival or fresh tumor tissue samples;
- Concomitant Medication, adverse events;
- Other examinations decided by the investigator according to the patient's condition.

Assessments within 7 days before study initiation:

- Complete physical examination: height, weight, blood pressure;
- Vital signs, ECOG PS;
- Quality of Life Questionnaire;
- Serum pregnancy test: HCG Testing, only for women of childbearing potential;
- 12 Couplet ECG (with special attention to QTc), echocardiography;
- Hematology and urinalysis, Stool routine (including fecal occult blood), Coagulation test (PT, APTT, TT, Fbg, INR), D-Dimer, Thyroid function test Amylase, lipase, markers of myocardial damage;
- Vascular endothelial growth factor VEGF, Tumor markers (SCC, CEA), Lymphocyte immunoassay;
- Blood biochemistry and liver function: alanine aminotransferase (ALT), aspartate aminotransferase (AST), glutamyl transpeptidase (GGT), total bilirubin (TBIL), direct bilirubin (DBIL), alkaline phosphatase (ALP), total protein (TP), albumin (ALB); renal function: blood urea nitrogen (BUN)/urea (Fig. UREA ), creatinine (Cr); blood glucose (GLU); electrolytes: potassium (K), sodium (Na), chloride (Cl), calcium (Ca), magnesium (Mg), inorganic phosphorus (PHOS); blood lipid four items: total cholesterol (TC), triglyceride (TG), high-density lipoprotein (HDL), low-density lipoprotein (LDL); uric acid (UA); ;
- Concomitant medication, Adverse events;
- Other examinations decided by the investigator according to the patient's condition.

6.2 During the study

- Blood pressure monitoring was completed and recorded by the patients themselves during the administration, and blood pressure was monitored regularly every week;
- ECOG PS will be performed every cycle, and comprehensive physical examination will be performed every cycle: body weight, vital signs and physical examination of various organs;
- Adverse reactions during treatment, including nausea, vomiting, diarrhea, and abdominal distension, were recorded. Observe and record various clinical manifestations during medication;
- Hematology: red blood cell count (RBC), hemoglobin (Hb), hematocrit (HCT), platelet count (PLT), white blood cell count (WBC), neutrophil count (NEUT), lymphocyte count (LYM), monocyte count (MONO), eosinophil count (EOS), basophil count (BASO); Once every cycle. If neutrophils ≤ 1×10 ^9^ /L or platelets ≤ 50×10 ^9^ /L, the frequency of re-examination should be increased (once every 2 ~ 3 days). In case of dose delay or dose adjustment due to hematological toxicity in any cycle, blood routine should be re-examined every week;
- Blood biochemistry: liver function: alanine aminotransferase (ALT), aspartate aminotransferase (AST), glutamyl transpeptidase (GGT), total bilirubin (TBIL), direct bilirubin (DBIL), alkaline phosphatase (ALP), total protein (TP), albumin (ALB); renal function: blood urea nitrogen (BUN)/urea (UREA), creatinine (Cr); blood glucose (GLU); electrolytes: potassium (K), sodium (Na), chloride (Cl), calcium (Ca), magnesium (Mg), inorganic phosphorus (PHOS); blood lipid panel 4: total cholesterol (TC), triglyceride (TG), high-density lipoprotein (HDL), low-density lipoprotein (LDL); uric acid (UA); test once every cycle.
- Routine urine and stool tests should be performed at least once every 2 cycles;
- Coagulation, thyroid function, amylase and lipase; perform tests once every cycle during initial treatment and once every even cycle during maintenance treatment;
- Electrocardiogram (with special attention to QTc) and myocardial injury markers are examined once every cycle. If chest pain, palpitation and other symptoms occur, additional echocardiography and myocardial enzymes (CK, CK-MB), troponin T and/or I, B-type natriuretic peptide (BNP) should be performed;
- During the study, efficacy was evaluated every 2 cycles during the initial treatment period and every 3 cycles during the maintenance treatment period. The number of assessments or examinations may be increased appropriately according to the patient's condition;
- Vascular endothelial growth factor VEGF, tumor markers (SCC, CEA), lymphocyte immunoassay; examination with imaging time points.
- During the study, quality of life questionnaire will be performed every 2 cycles during initial treatment and every 3 cycles during maintenance treatment;
- Concomitant medications and any observed adverse events should be recorded during this period.
- Other examinations decided by the investigator according to the patient's condition.

6.3 Dropouts

All patients who completed the informed consent form and are screened as eligible for the trial had the right to withdraw from the clinical trial at any time. No matter when and why the subjects withdraw from the study, as long as they do not complete a cycle of clinical trials and cannot be evaluated for safety and efficacy, they are all drop-outs (After enrollment, patients who progressed with clear medical evidence are not considered as drop-outs, and imaging are required. Patients who terminates study treatment because of intolerable toxicity after enrollment are not considered drop-outs). When a patient dropped out, the investigator must fill in the reason for dropout in the CRF, complete all the evaluation items that can be completed, and carefully fill in the visit record in the CRF. Patients who only undergo screening but withdraw from the study without obtaining the drug will not be considered as dropouts.

6.4 End of treatment and follow-up

6.4.1 End-of-treatment visit

The end of treatment is defined as the date on which the investigator decided that the patient should stop all study medication. If the decision of drug termination is made within 2 weeks of the last treatment visit, an end of treatment visit is not required unless deemed necessary by the investigator. If decided more than 2 weeks after last treatment visit (due to disease progression or other reasons), an end of treatment visit is required.

- Complete physical examination: body weight, blood pressure;
- Vital signs, ECOG PS;
- Quality of Life Questionnaire;
- Echocardiogram;
- ECG, blood routine, blood biochemistry, fasting blood glucose, urine routine, stool routine and myocardial injury marker;
- Coagulation function test (PT, APTT, TT, Fbg, INR, D-Dimer), thyroid function test, amylase and lipase;
- Vascular endothelial growth factor VEGF, tumor markers (SCC, CEA), lymphocyte immunoassay;
- Imaging examination (CT/MRI);
- Concomitant medications, adverse events.

6.4.2 Follow-up Visit

Safety follow-up: A safety follow-up visit will occur 28 ± 7 days within last dose of the investigational drug. AEs and SAEs that remained unresolved had to be reported at least 28 days after the last dose or had returned to NCI-CTCAE V5.0 grade 1, returned to baseline, or are considered irreversible, whichever the longest. If the subject is scheduled to start a new antineoplastic therapy before the end of the safety follow-up visit, the safety follow-up visit is to begin before the new antineoplastic therapy. Once a new antineoplastic therapy is initiated, the subject enters a survival follow-up period.

After completion of the safety follow-up period, enter into the survival follow-up period. The first survival follow-up period (including telephone follow-up) occurred 8 weeks (± 7 days) after the last safety follow-up and occurred on an 8-week (± 7 days) basis until the subject died, lost of follow-up, study termination by the sponsor, or other study closure criteria are met, whichever occurrs first. The investigator may follow the subject, family, or the responsible physician by telephone to collect whether other anticancer therapy was administered to the subject prior to this visit. If other treatments are used, the treatment regimen and cycle number and outcome must be recorded. The patients are followed up until death and the relevant cause of death and the specific time are recorded to obtain the overall survival (OS).

For subjects who terminate treatment due to intolerance or other reasons, if no radiographic progression is observed, radiographic assessment should still be performed at the original frequency until disease progression or initiation of other anti-tumor therapy (except anti-tumor therapy with traditional Chinese medicine), and disease progression should be obtained as far as possible.

6.5 Unscheduled Visits

Unscheduled visits may be performed at any time at the discretion of the investigator and appropriate clinical and laboratory tests may be performed based on AEs or other findings.

7. Efficacy evaluation

During the clinical use of similar drugs of TQB2450, the patient had pseudoprogression. The efficacy is assessed according to RECIST 1.1.

The methods of radiographic assessment (CT or MRI) will be determined by the investigator. However, the assessment methods, machines, and technical parameters should be consistent throughout the study period. Contrast media is required for subjects without contraindications. For subjects who had received a radiographic assessment within 28 days (within 3 mounth for bone scan) before the first dose using the same procedure in the same center, the radiographic assessment could be used as the baseline data in this trial. The baseline tumor assessment should include enhanced CT or MRI of the chest, abdomen, and pelvis (except for subjects with an allergy to contrast media). During the screening period, a cranial plain scan plus enhanced MRI, and bone scan is required for all subjects. During screening, brain examination (plain scan plus contrast-enhanced MRI is recommended, or plain scan plus contrast-enhanced CT is allowed if MRI was not available because of contrast allergy or other reasons) and bone scanning (for subjects without bone metastases during screening, determined according to clinical indicationst by study physician) should performed. Imaging should be performed at all suspicious sites. CT or MRI of the neck, chest, abdomen, pelvis, and other areas with lesions, with additional imaging of suspicious areas was recommended during the trial. If the subject retained the uterus, the preferred method of pelvic imaging was MRI (if applicable).

Tumor imaging will be performed every 2 cycles during initial treatment, and every 3 cycles during the maintenance treatment until the patient has tumor imaging confirmed disease progression, and assessment frequency will not change the due to patient's delay or interruption of treatment. Patients who discontinued the study treatment for reasons other than progressive disease were required to undergo imaging at this frequency until the initiation of new antineoplastic therapy, radiographic evidence of disease progression, voluntary withdrawal, or death, whichever came first. Timely imaging was required when the subject was out of the group for any reason (+7 days, no repeat examination was required if the previous examination was not more than 4 weeks before the end of treatment). Imaging conditions should be the same as at baseline (slice thickness, contrast material, etc.). A window of +7 days was allowed for imaging, and unscheduled imaging could be performed when disease progression was suspected (e.g., worsening symptoms).

Progressedn disease (PD) validation process: The efficacy evaluation of this study is accessed by the site investigators.

7.1 Tumor Definition

- Measurable lesions:

Measurable visceral disease: Lesions that can be accurately measured in at least one dimension with the use of a CT scan with a slice thickness of 5mm or less and that most often have a diameter (required documentation) of 10mm or more or that are at least twice the slice thickness with the use of a CT or MRI scan with a slice thickness of more than 5mm.

Measurable pathological lymph nodes: Lymph nodes with pathologic enlargement, a high suspicion of metastasis, and a short axis measurement of 15mm or more must be considered malignant on CT evaluation. The short axis refers to the longest linear dimension perpendicular to the longest diameter of the lymph node evaluated in the same plane as the acquisition scan. Only measurable lesions could be selected as target lesions.

- Non-measurable lesions include:

1. Small visceral metastatic lesions less than 10 mm in the longest dimension or twice the slice thickness if the slice thickness is greater than 5 mm.
2. Abnormal and suspicious metastatic lymph nodes with short axis ≥ 10 mm and < 15 mm;
3. Lesions that are truly non-measurable (e.g., ascites carcinomatosis). All non-measurable lesions can only be selected as non-target lesions.

- Target lesions

All measurable lesions, up to two per organ, and a total of 5 lesions representing all involved organs/tissues should be considered as target lesions;

Target lesions (organs with longest diameter and lymph nodes with short axis measurement) should be selected based on lesion size. Target lesions should be representative of all involved organs/tissues, and lesions with reproducible repeated measurements should be selected;

When recording tumor measurements, the most common diameter of each non-nodal target lesion should be recorded. For measurable pathological lymph nodes that can be considered as target lesions, it’s short-axis measurement value should be used in combination with the measurement value of non-nodular (i.e., organ lesion) target lesions. Therefore, when a complete response (CR) occurs in an abnormal lymph node that is a target lesion, the sum of its diameters will not decrease to a value of zero.

1. Target lesions were followed and measured at each subsequent time point.
2. The sum of diameters for all target lesions will be calculated and recorded. The baseline sum will be used as a reference value to further characterize the objective tumor assessment of lesions in the measurable dimension.

Assign a single measurement to all target lesions, regardless of size. If no measurement value can be assigned, "too small to measure" will be provided Options. A value of 0 is assigned only when there is a complete response.

"Not Evaluable" lesions options are only used for those lesions that cannot be read for technical reasons, e.g: 1. CT artifacts. 2. patient position resulting in obstruction or inability to see the lesion. 3. The whole lesion cannot be seen due to the thick CT slice. If a lesion is divided into two lesions, the longest diameters of the fragmented portions should be added together to calculate the target lesion sum. If two lesions are fused, there may be a plane left between them that helps to obtain the largest diameter measurement for each individual lesion. If these lesions truly coalesce and are no longer distinguishable, the vector of the longest diameter in this instance should be used as the "coalescing lesion" of the longest diameter.

- Non-Target Lesions

Non-target lesions include all non-measurable and measurable lesions that are not selected as target lesions. Lymph nodes with short axis < 10 mm are considered non-pathological and should not be recorded;

Any indeterminate lesion without a definite diagnosis (e.g., an unspecified solitary pulmonary nodule without biopsy, an unspecified thyroid mass without needle aspiration biopsy) that cannot be distinguished from a benign lesion can be considered a non-target lesion;

All other diseases (or lesion location) including pathological lymph nodes should be considered non-target lesions and recorded at baseline. Although measurements are not required, their presence, disappearance, or unequivocal progression should be followed during the study;

It is possible to record multiple non-target lesions involving the same organ as one item in the eCRF.

7.2 Primary Efficacy Measures:

- Progression-free survival (PFS)

Defined as the time from the date of enrollment to the date of first evidence of PD or death from any cause, whichever occurs first.

If a subject did not experience PD during the trial, PFS is defined as the last date the subject is confirmed to be progression-free. Subjects who discontinue the trial for reasons other than PD (without subsequent imaging) and subjects who receive post-trial treatment will be censored at the time of discontinuation or the start of post-trial treatment. When a subject is not censored at the time of discontinuation from the trial or the time of initiation of post-trial treatment, a pre-planned sensitivity statistical analysis will further confirm PFS solely on the basis of the time to event of radiologically confirmed progression. New onset of other neoplasms is not considered a disease progression event and is not censored.

If imaging studies and evaluations showed progression, the date of progression is not the time of imaging that first shows progression but rather the time of imaging that definitively confirmed progression. If PD is diagnosed by other clinical means, the date of diagnosis is taken as the date of PD.

7.3 Secondary Efficacy Measures:

- Overall survival (OS):

Defined as the time from the first dose to death due to any causes.

- The objective response rate (ORR):

Defined as the proportion of patients whose tumor shrinkage reaches a certain amount and remains for a certain period of time, including CR and PR. RECIST 1.1 Criteria to assess objective tumor response. Subjects must have measurable tumor lesions at baseline. Efficacy evaluation is classified as complete response (CR), partial response (PR), stable (SD) and progressed disease (PD) according to RECIST 1.1 criteria.

- Disease control rate (DCR):

Percentage of confirmed cases including CR, PR and SD among efficacy evaluable patients.

- Duration of response (DoR)

Time from the first assessment of CR or PR to the first assessment of PD or death from any cause.

8. Safety evaluation

8.1 Adverse Events

AEs are defined as any untoward medical occurrence in a patient or clinical investigation subject administered a pharmaceutical product and which does not necessarily have to have a causal relationship with this treatment. Thus, AEs could be any unfavorable and unintended sign (including an abnormal laboratory finding), symptom, or disease temporarily associated with the use of a medical treatment or procedure regardless of whether it is considered related.

This study requires collection of any untoward AEs, regardless of causal relationship to the study drug, that occurs from the time the subject signs the informed consent through 28 days after the last dose or initiation of new antineoplastic therapy (whichever comes first). It should be noted that clinical adverse medical events occurred after signing the informed consent form and before the first dose are recorded as medical history/concomitant diseases rather than as AEs, unless one of the following conditions is met: injury/damage caused by any clinical laboratory test procedure; adverse events caused by discontinuation related to the trial protocol; adverse events caused by drugs other than the investigational product taken as part of the treatment regimen, etc.

8.2 Assessment of Adverse Events

The nature and severity of adverse events were assessed according to the National Cancer Institute Common Toxicity Criteria (NCI- CTCAE v5.0).

Adverse event terms not included in NCI-CTCAE v5.0 were graded according to the following CTCAE grading principles:

Grade 1: mild; No symptoms or mild signs; Only clinical or diagnostic observation; No medical stem is needed

Grade 2: moderate; The need for minimal, local or non-invasive treatment; Age-appropriate functions of daily living (e.g., cooking, shopping, using the telephone, and managing money) are limited.

Grade 3: severe or clinically significant but not immediately life-threatening; Hospitalization or prolonged hospitalization; Disabling; The ability to perform activities of daily living (e.g., bathing, dressing and undressing, eating, toileting, and taking medications) was limited but not bedridden.

Level 4: resulting in life-threatening consequences; Urgent treatment is required.

Grade 5: death related to AE.

Note: * Instrumental activities of daily living refer to preparing meals, buying clothes, using the telephone, managing money, etc.

* Self-care activities of daily living refer to bathing, dressing and undressing, eating, washing, taking medications, etc., and not bedridden.

8.3 Recording of Adverse Events

During the reporting period of AEs, the investigator(s) are required to record any AEs, including SAEs, in the CRF/eCRF. For the report of AEs, the investigator(s) are required to use the correctly standardized medical terminology rather than colloquialism and abbreviations. The start date, severity grade as per NCI-CTCAE v5.0, stop date, causality to study drugs, effects on the trial, concomitant therapy, and recovery will be recorded.

Diagnosis vs. symptoms and signs

A diagnosis (if known) should be recorded on the CRF/eCRF rather than individual signs and symptoms (e.g., record only liver failure or hepatitis rather than jaundice, asterixis, and elevated transaminases). However, if a constellation of signs and/or symptoms cannot be medically characterized as a single diagnosis or syndrome at the time of reporting, each event should be recorded on the CRF/eCRF as AEs. If a diagnosis is subsequently established, all previously reported AEs based on signs and symptoms should be nullified and replaced by 1 AE report based on the single diagnosis.

Start time and end time

When an adverse event progresses to a serious adverse event, the time when the adverse event is upgraded to a serious adverse event is used as the start time of the serious adverse event.

The end time is not collected when the subject died, which did not lead to ‘Death’ for adverse events of direct cause, the end time should be vacant and the outcome is " Persistent ". If judged to cause ‘Death’ For adverse events of direct or primary cause, the end time is the time of subject's death, and the outcome is ‘Death’.

Adverse Events Secondary to Other Events

In general, AEs occurring secondary to other events (e.g., cascade events or clinical sequelae) should be identified by their primary cause, except for severe or serious secondary events. However, medically significant AEs occurring secondary to an initiating event that are separated in time should be recorded as independent events on the CRF/eCRF. All AEs should be recorded separately as primary or secondary events if it is unclear as to whether the events are associated.

Persistent, intermittent, or single adverse events (frequency of adverse events)

Persistent AE extends continuously, without resolution between cycles/courses, such as the upper respiratory tract infection lasting 5 days. The event must only be reported once unless the grade becomes more severe. For severity grade, the highest severity grade should be recorded.

Intermittent AE occurs and resolves during a cycle/course of therapy, but without clinically significant outcomes, such as nausea and vomiting lasting for several days with the intermittent resolution, and persistent hypertension with intermittent resolution. The event must only be reported once. For severity grade, the highest severity grade should be recorded.

Single AE is one that occurs independently or only once during therapy, such as falls and one vomiting event during the trial. The event must only be reported once.

It should be noted that these above events are recurrent after clinically significant resolution and meanwhile have no course continuity with the former, each recurrence of an AE should be recorded separately on the CRF/eCRF.

Laboratory abnormalities or vital signs

Laboratory test results will be recorded on the laboratory results pages of CRF. Not all abnormal laboratory tests and vital signs will be reported as AEs. Investigator(s) have the responsibility to review all laboratory findings and vital signs. Medical and scientific judgment should be exercised in deciding whether an isolated laboratory abnormality should be classified as an AE. Any abnormalities that meet one or more of the following conditions for clinical significance will be reported as AEs:

- Accompanied by clinical symptoms;
- Leading to a treatment change (e.g., dose modification, interruption, or discontinuation);
- Requiring medical intervention or the change of concomitant therapy (e.g., concomitant medication, new treatment, treatment interruption, discontinuation, or any other change);
- Have significant clinical significance as judged by the investigator.

If a clinically significant laboratory abnormality is a sign of a disease or syndrome (e.g., increased ALT/AST and bilirubin caused by hepatic dysfunction), only the diagnosis should be recorded on the CRF/eCRF (hepatic dysfunction). Otherwise, the abnormality should be recorded along with a descriptor indicating if the test result is above or below the normal range. If the abnormalities have corresponding standard clinical terms, the standard clinical terms should be recorded (e.g., an increase in blood potassium to 7.0 mmol/L should be recorded as hyperkalemia).

Pre-existing Symptoms or signs

A preexisting medical condition should be recorded as an AE only if the frequency, severity, or character of the condition worsens during the study. When recording such events, it is important to convey the concept that the preexisting condition has changed by including applicable descriptors (e.g., more frequent headaches, and aggravated hypertension).

Surgery

If the disease for surgery is definite, the disease should be recorded as AE rather than surgery (e.g., for subjects who underwent inguinal herniorrhaphy, AE is inguinal hernia rather than inguinal herniorrhaphy). Otherwise, the surgery should be recorded as AE (e.g., for subjects who underwent abbreviated laparotomy, abbreviated laparotomy is AE).

Overdose

Overdose is not an AE. However, any untoward medical occurrence that results from this is an AE, which should be recorded and reported in the original medical record and eCRF report.

8.4 Follow-up of Adverse Events

Investigator(s) are required to follow up all AEs until any of the following occurs:

- AEs resolve to baseline;
- No further remission will be expected by the investigator(s);
- Death;
- Lost to follow-up;
- AEs that are not related to the study treatment confirmed by the investigator(s);
- Starting new antitumor treatment;
- End of clinical or safety data collection, or the database lock;
- No clinical or safety data are collected, or the database is eventually closed.

The outcomes of each AE (including the date of resolution and death) need to be recorded in the CRF/eCRF.

8.5 Criteria for judging the correlation between drug and adverse event

Investigator(s) are required to assess the causality of AEs to study drugs, according to the following 5 criteria:

(1) Whether the administration time and the suspected AEs exhibit a reasonable relationship;

(2) Whether the suspected AEs fulfill the criteria for the typical reactions of the drug;

(3) Whether the suspected AEs can be explained by the effects of the combined drug, patient’s clinical condition, or other therapies;

(4) Whether the suspected AEs disappear or are mitigated after drug discontinuation;

(5) Whether the same AEs recurred after repetitive administration of the study drugs.

|  | 1 | 2 | 3 | 4 | 5 |
| --- | --- | --- | --- | --- | --- |
| Definite | + | + | - | + | + |
| Probable | + | + | - | + | ? |
| Possible | + | + | ± | ± | ? |
| Unlikely | + | - | ± | ± | ? |
| Unrelated | - | - | + | - | - |

Note: +, Yes; -, No; ±, probably Yes or No; ?, unknown.

AEs will be calculated as the sum of definitely-related, probably-related, and possibly-related events.

8.6 Serious Adverse Events (SAEs)

A serious adverse event (SAE) is any untoward medical occurrence that results in death, is life-threatening, results in persistent or significant disability or incapacity, requires inpatient hospitalization or prolongation of existing hospitalization, or is a congenital anomaly or birth defect after the subject has received investigational product.

(1) Leading to death;

(2) Life-threatening: It refers to that the subject was at risk of death at the time of adverse event, but does not refer to the assumption that the adverse event may cause death if it is more serious;

(3) Permanent or severe disability or loss of function: adverse event results may cause serious inconvenience or interference with the normal life and activities of subjects;

(4) Requires hospitalization or prolongation of hospitalization: The subject has to be hospitalized for treatment due to adverse event or has been prepared to be discharged but has prolonged hospitalization due to adverse event; it needs to be clarified that the reason leading to this condition is due to adverse event, rather than admission due to elective surgery, non-medical reasons, etc.;

(5) Congenital abnormalities or birth defects: the subject's offspring appear malformations or congenital functional defects;

(6) Other important medical events: Medical and scientific judgment must be used to determine whether expedited reporting is appropriate for other situations. For example, important medical events may not be immediately life-threatening, fatal or hospitalized, but if medical measures are required to prevent one of the above situations, they are usually considered serious. For example, major treatment in the emergency room or allergic bronchospasm at home, cachexia or convulsion without hospitalization, drug dependence or addiction.

Death

For reporting death events, the death due to AE should be recorded as the single medical concept on the CRF/eCRF and reported as SAE. If the cause of death is unknown, “unexplained death” should be recorded on the CRF/eCRF, reported as SAE provisionally, and confirmed by further investigation. If the cause of death later becomes available, the “unexplained death" should be replaced by the established cause of death.

Disease progression

Symptoms or signs of progression were not recorded as adverse events if they occurred as expected. Hospitalizations solely due to symptoms or signs associated with the progression of this disease should also not be considered an SAE. If the symptoms could not be clearly caused solely by disease progression, or if the symptoms or signs of disease progression were more severe than expected, or if the investigators believed that tumor progression was related to the trial drug administration or study procedure, the relevant clinical symptoms could be recorded as an AE and should be reported to the SAE.

Hospitalization, prolonged hospitalization

The following conditions leading to hospitalization or prolongation of existing hospitalization are not classified as SAEs:

The hospitalization or prolongation of existing hospitalization required by protocol (e.g., administration and efficacy evaluation);

An elective hospitalization for a pre-existing condition unrelated to the study indication. For example, the planned surgery or treatment before study or the scheduled surgery or treatment after enrollment. However, hospitalization for surgery or treatment due to disease worsening (surgery or treatment in advance) will be classified as SAEs.

Pregnancy

If a female subject or a female partner(s) of a male subject becomes pregnant during the trial, the investigator(s) should be informed immediately. Investigator(s) are required to report to the sponsor within 24 hours of learning of its occurrence. Pregnant subjects should immediately stop using the study drug. The investigator should counsel the patient, and discuss the risks of continuing the pregnancy and the possible effects on the fetus. Pregnant subjects will be monitored until the end of pregnancy. All pregnancies within 30 days of the last administration will be reported to the investigator(s).

Both induced and spontaneous abortions should be reported as SAEs. Any congenital anomalies/birth defects in infants born to female subjects or female partners of male subjects who had taken study drugs should be reported as SAEs.

8. 7 Reporting system of serious adverse events

Serious adverse events were reported from the time a subject signed informed consent through 28 calendar days (inclusive) after the last dose of study drug. In case of serious adverse event, whether it is an initial report or a follow-up report, the Clinical Study Serious Adverse Event (SAE) Report Form must be completed immediately, signed and dated, and the Pharmacovigilance Department, Clinical Research Associate, Principal Investigator and Ethics Committee of the leading site of Chia tai tianqing Pharmaceutical Group CO., LTD should be informed immediately within 24 hours after the investigator is informed.

Serious adverse events occurring after administration of investigational product 28 are not reported unless they are suspected to be related to the study drug.

For serious adverse events, the symptoms, severity, correlation with the study drug, occurrence time, treatment time, measures taken, follow-up time and method and outcome should be recorded in detail. If the investigator considers a SAE to be unrelated to the investigational drug and potentially related to the study conditions (e.g. discontinuation of original treatment, or comorbidities during the trial), the relationship should be detailed in the narrative section of the SAE CRF. If the intensity of an ongoing SAE or its relationship to the study drug is changed, a follow-up SAE report should be sent to the sponsor immediately. All serious adverse events should be followed up until recovery or stabilization.

8.8 Reporting procedures of SAEs

Any serious adverse event occurred during the clinical study and within 28 days after drug withdrawal must be immediately reported to the monitor of the cooperative unit, principal investigator of the unit responsible for clinical study, ethics committee and provincial food and drug administration where each study site is located.

Table 10 Required Reporting Units and Contact Information after SAE

| Units | Contact Person | Contact Mode |
| --- | --- | --- |
| Henan Cancer Hospital | Ethics Committee | Phone/Fax: 0371 - 65588251 |
| Chia Tai Tianqing Pharmaceutical Group Co., Ltd. | Pharmacovigilance | Email: TQB2450@cttq.com |
| China Food and Drug Administration Drug Department of Registration | | Address: No.1 Beiluyuan, Zhanzhou Road, Xicheng District, Beijing  Postal code: 100053  Tel.: 010-88331134  Fax: 010- 88363228 (preferred) |

8.9 Symptomatic treatment of common adverse reactions

8.9.1 Palmar-plantar erythrodysesthesia syndrome

Hand-foot syndrome is palmar-plantar dysesthesia or acral redness, marked discomfort, swelling, tingling, and more pronounced manifestations in compressed or stressed areas. Cancer patients may appear during chemotherapy or molecular targeted therapy.

Grade 1 is characterized by painless minor skin changes or dermatitis (e.g., erythema, edema, hyperkeratosis); Grade 2 is characterized by painful skin changes (e.g., flaking, blistering, bleeding, swelling, hyperkeratosis); affecting instrumental activities of daily living; and Grade 3 is characterized by severe skin changes (flaking, blistering, bleeding, edema, hyperkeratosis) with pain; and affecting personal activities of daily living.

For patients with Grade 1 toxicity, supportive treatment is often not required, and for patients with Grade 2 or higher toxicity, consider the following symptomatic and supportive treatments: including: enhanced skin care, keeping the skin clean and avoiding secondary infections; avoiding pressure or friction; use of emollients or lubricants, topical application of lotions or lubricants containing urea and corticosteroid components; and topical antifungal or antibiotic treatment if necessary.

8.9.2 Hypertension

Blood pressure should be monitored daily for the first 6 weeks of study medication. Blood pressure increased, active communication with the physician is required. When elevated blood pressure occurs, it can mostly be controlled by conventional antihypertensive treatment. Uncontrollable increases in blood pressure can generally be relieved by reducing the dose of targeted drugs or discontinuing the drug.

Recommendations for staging and routine management of hypertension

Hypertension refers to a pathological increase in blood pressure, which is repeatedly measured and exceeds 140/90 mmHg. Severity Grading:

Grade 1: prehypertension: (systolic blood pressure 120-139, diastolic blood pressure 80-89 mmHg) without antihypertensive drugs, only blood pressure was monitored. Grade 2: hypertension in the first stage (systolic blood pressure: 140-159 mmHg, diastolic blood pressure: 90-99 mmHg); medical intervention is required; repeated or persistent (≥ 24 h), symptomatic systolic blood pressure increase > 20 mmHg or previous normal range increase > 140/90 mmHg; blood pressure should be monitored during treatment; most patients use thiazide diuretics, and angiotensin-converting enzyme inhibitors (ACEI), angiotensin II receptor blockers (ARB), β-blockers and calcium channel blockers can also be considered. Grade 3: hypertension in the second stage (systolic blood pressure greater than or equal to 160 mmHg, diastolic blood pressure greater than or equal to 100 mmHg); medical intervention indicated; multi-drug therapy indicated, usually thiazide diuretics and ACEI or β-blockers or calcium channel blockers. Grade 4: Life-threatening (e.g., malignant hypertension, transient or persistent neurologic impairment, hypertensive crisis); urgent intervention indicated. At present, there is no uniform classification at home and abroad; recently, it has been divided into two types from the perspective of clinical treatment:

(1) Hypertension emergencies, diastolic blood pressure > 120 mmHg, accompanied by acute or progressive target organ damage, such as cerebral infarction, intracranial or arachnoid hemorrhage, hypertensive encephalopathy, etc., of which progressive or rapidly progressive hypertension on the basis of chronic essential hypertension is the most common (about 40% to 50%); (2) Hypertension urgencies, diastolic blood pressure > 120 mmHg without or only mild organ damage. Nifedipine or nifedipine were used to rapidly lower blood pressure, diazepam and phenobarbital were used to stop convulsion, fursemide and mannitol were used for dehydration, sodium excretion and reduction of intracranial pressure;

Once a patient develops hypertensive crisis, the administration should be terminated and the patient should be withdrawn from the clinical study.

8.9.3 Management of Diarrhea

Supportive care may be given for grade 1 to 2 diarrhea, such as starting treatment with loperamide at the earliest (e.g., 4 mg orally, followed by 2 mg orally every 2 hours until diarrhea resolves).

8.9.4 Management of gastrointestinal bleeding

Gastrointestinal bleeding, including fecal occult blood (+ +) or more, hematemesis or bloody stools, should be actively treated symptomatically. Patients with upper gastrointestinal bleeding should be fasted, and given antacid, protection of gastric mucosa, hemostasis (hemostatic acid, reptilase, etc.), blood transfusion and supportive treatment, if necessary, octreotide, etc.; patients with lower gastrointestinal bleeding should be given hemostasis, blood transfusion and supportive treatment, etc.; patients with uncontrolled bleeding should be treated with surgical assistance.

8.9.5 Recommendations for Management of Proteinuria

Proteinuria was closely monitored in all patients throughout the treatment period and enhanced in those with a history of hypertension; 24-hour urinary protein measurement was required in those with two consecutive urinary protein ≥ + +.

After presence of proteinuria, dose adjustment should follow the following principles: continue treatment as planned when urine protein is + or < 3 g/24 h and provide symptomatic treatment; if urine protein is ≥ 3 g/24 h, suspend treatment and provide symptomatic treatment until urine protein is < 3 g/24 h and then reduce the dose by one dose level; if urine protein is ≥ 3 g/24 h after dose reduction, resume treatment at one dose level after recovery to < 3 g/24 h. However, if urine protein ≥ 3 g/24 h occurs for the third time, the test will be terminated.

If grade 4 proteinuria (nephrotic syndrome) develops, the drug should be permanently discontinued and the patient should be withdrawn from the clinical study.

8.9.6 Management of Hyperlipidemia and Hyperglycemia

The management of hyperlipidemia should consider the patient's pretreatment status and dietary habits. In addition to diet, grade 2 or higher hypercholesterolemia (i.e. ≥ 7.75 mmol/L), or grade 2 or higher hypertriglyceridemia (i.e. ≥ 2.5 x upper normal limit), should be treated with HMG-CoA reductase inhibitors (atorvastatin, etc) or appropriate lipid-lowering drugs.

8.9.7 Gastrointestinal Toxicity

Severe nausea and vomiting are the main limiting toxicities, and acute vomiting generally occurs 1 – 2 hours after administration; at present, 5-HT3 receptor antagonists ondansetron, granisetron, and ramosetron are widely used for chemotherapy-induced vomiting in clinical practice. Dexamethasone: To prevent delayed symptoms, oral dexamethasone may be given, either alone or in combination with metoclopramide and diphenhydramine.

8.9.8 Renal Toxicity

Generally, the dose mainly produces tubular injury, which is seen 10 to 15 days after treatment and is reversible; repeated high-dose treatment can cause persistent mild to moderate renal damage.

Prevention and treatment of nephrotoxicity: In order to reduce the deposition of platinum in the kidney and reduce the nephrotoxicity of platinum drugs, 1 ~ 2 liters of water or infusion can be used in advance before administration.

Administration of sodium thiosulfate: Sodium thiosulfate combined with cisplatin can reduce the degree of activation, and its concentration in the kidney is high, which can reduce the reabsorption of sodium thiosulfate in the renal tubules.

Give cytoprotective drugs: some cytoprotective drugs such as amifostine, organic selenium preparations, glutathione, vitamin C, etc. can play a role in protecting renal tubules and reducing the nephrotoxicity of platinum drugs.

8.9.8 Hematotoxicity

The most common is myelosuppression, which is characterized by a decrease in white blood cells, granulocytes, and platelets.

Prevention and treatment of hematotoxicity

Correction of leukopenia: This process can be divided into two stages: prevention and treatment, and granulocyte colony-stimulating factor (G-CSF) and granulocyte macrophage colony-stimulating factor (GM-CSF) can be used, respectively.

Correction of thrombocytopenia: 1L-11 can be used to increase cytokines in platelets, and the recommended dose is 50 μg/ (kg.d), generally used at least after 6–24 hours of chemotherapy, with the risk of toxic water and sodium retention.

Correction of anemia: The destruction and inhibition of hematopoietic system by tumors and bone marrow injury caused by chemotherapy can lead to the occurrence of anemia. When the patient's peripheral hemoglobin is male < 110 g/L Erythropoietin can be used when women are < 100 g/L, and 150 IU/kg three times a week is recommended; after 4 weeks, the red blood cell increase is less than 0.1 g/L, and the dosage can be increased to 300 IU/kg three times a week; it is still ineffective and discontinued at 8 weeks.

8.9.9 Neurotoxicity

Sodium channel blockers carbamazepine plus gabapentin can be used to reduce the occurrence of neurotoxicity, or some trophic nerve drugs such as vitamin B1, vitamin B6 vitamin C, etc., can improve the symptoms of poisoning. In addition, interferon has the effects of reducing delay and preventing neurotoxicity caused by interferon. For patients with serious adverse reactions, timely consideration or drug withdrawal should be given and symptomatic treatment should be given.

The types of immune-related adverse events caused by TQB2450 injection and the treatment recommendations are shown in the Guidelines for the Management of Toxicity Related to Immune Checkpoint Inhibitors of Chinese Society of Clinical Oncology (CSCO) 2021 ".

9. Quality of life

The quality of life of the patients was assessed by collecting questionnaires from many aspects. The assessment of health-related quality of life questionnaire is an exploratory part of this project, which aims to collect various information on drug application as early as possible.

Health-related quality of life questionnaires need to be completed at specified time points. Patients should complete questionnaires prior to undergoing various clinical examinations and assessments, before being informed of any new disease information, and before starting any new therapy to avoid answers being affected by the physician or new condition. The questionnaire will be kept as source data.

The investigator will review the patient's questionnaire and assess the patient's clinical condition when completing the questionnaire. Any adverse event found by the clinical assessment should also be recorded in the CRF.

Quality of life questionnaires: EORTC Quality of Life Questionnaire (QLQ-C30), esophageal cancer module (QLQ-OES18) and EQ-5D;

10. Data Management and Statistical Analysis

10.1 Case Report Forms

The investigator or authorized designee should complete relevant information in the electronic case report form by using the electronic entry system (EDC) within the specified time. All relevant data of each follow-up visit of each subject during the trial should be timely and truly recorded, and confirmed and signed. To guarantee the patient's right to privacy, the patient's name will be coded.

10.2 Database establishment

The data manager designated by the statistician should complete the electronic case report form document and system in advance, and prompt the investigator or authorized person to verify and modify the questionable data in time. The database was reviewed correctly and the data were locked by the principal investigator, data manager, statistician and monitor. Data cannot be entered and modified by irrelevant personnel to ensure data security. Electronic case report data must be backed up. Any data changes can be made only after the consent form is signed by the principal investigator, statistician and data manager.

10.3 Data Lock

The automatic verification system checks the data deviation in the eCRF and generates the corresponding query table, allowing the study site personnel to modify and verify the entered data. The system automatically saves all data modification trajectories. It is then transmitted to the data statistics unit through a secure virtual private network. When the principal investigator, co-organizer, statistical analyst and data management personnel are present at the same time, the analysis dataset shall be determined, the reviewed data shall be locked, and the locked data files shall not be changed in principle.

10.4 Selection of Statistical Analysis Data

- Full Analysis Set (FAS): The efficacy analysis was performed according to the intention-to-treat (ITT) principle in all patients who used the drug at least once.
- Per-protocol Set (PPS): Patients with at least one tumor response evaluation results, who are compliant with the study protocol, have good compliance, have not received the prohibited drugs during the study, and have completed the case report form.
- Safety analysis set (SS): All patients who used the investigational drug at least once and had safety record after treatment.

10.5 Statistical Analysis Plan

- **Efficacy analysis:** For efficacy indicators progression-free survival (PFS), overall survival (OS), duration of response (D OR), median time will be estimated using the Kaplan-Meier method and events and their 95% confidence intervals will be presented. The disease control rate (DCR = CR + PR + SD) and objective response rate (ORR = CR + PR) were calculated using Clopper-Pearson method and presented with 95% confidence intervals. Quality of life score: Number and percentage of subjects with different levels of each dimension will be calculated for EORTC quality of life questionnaire (QLQ-C30), esophageal cancer module (QLQ-OES18) and EQ-5D. The signed-rank test is used for intra-group comparisons when necessary, and the H-test is used for inter-group comparisons.
- **Safety analysis**: Safety analyses will be based on all treated subjects. All adverse events will be classified as NCI-CTCAE 5.0 Versions were graded, and descriptive statistics were mainly summarized, according to groups AE, SAE, ≥grade 3 AEs, drug-related AEs, drug-related SAEs, AEs leading to dose interruption / adjustment or treatment termination. The data are statistically summarized. Laboratory test results, vital signs, 12-lead electrocardiogram, echocardiography and other data, continuous indicators are analyzed by means, standard deviation, median, maximum, minimum and so on, and cross classification table is used for analysis of baseline and post-baseline conditions for qualitative data.

10.6 Sample Size Estimation

This study is an exploratory study. The sample size calculation for the cohort 1 is based on a historical median PFS of 5.7 months with immunochemotherapy regimens in patients with advanced ESCC. It is hypothesized that the combination of benmelstobart, chemotherapy, and anlotinib would achieve an expected median PFS of 9.8 months. With a 12-month enrollment period and 12 months of follow-up, approximately 27 events among 38 patients are anticipated, providing 80% power at a two-sided α of 0.05 to demonstrate superior efficacy compared to the historical control. Considering a 20% dropout rate, the plan is to enroll 48 patients.

In the group 2, 30 patients will be enrolled. After completion of group 1, the study of group 2 will be conducted.

11. Coordinator Investigator Responsibilities

11.1 Co-organizer

1. Provide the investigator with materials and other support, and explain the protocol and filling of various materials to the investigator before the clinical initiation;
2. Dispatch a clinical research associate for regular monitoring visits;
3. The CRA should make sure that he/she can keep in contact with the investigator by phone, fax and mail at any time.
4. The monitor will supervise the investigator to carry out the clinical study in accordance with the approved protocol, check the distribution and recovery of investigational drugs according to relevant regulations, and ensure the consistency between the trial records in the clinical trial and the data in the original report.

11.2 Investigator

1. Have received training on GCP and this trial protocol, and have time to carry out this trial according to the study protocol.
2. Patients should be informed in detail about the study before enrollment, and consent should be obtained from the patients and informed consent should be signed.
3. The investigator is obliged to take necessary measures to ensure the safety of patients. In case of any adverse reaction, the investigator should deal with it and report to the principal investigator immediately according to relevant regulations. Serious adverse reactions were followed up.
4. Carefully fill in the case report form in a timely manner;
5. Actively cooperate with the CRA in regular visits;
6. Complete retention of laboratory test records, clinical records, and the patient's original medical records;
7. In order to ensure the evaluation and supervision of clinical trials by China Food and Drug Administration and the sponsor, the study site shall uniformly preserve all the study data, including the confirmation of all the patients (can effectively check different records), all the original informed consent forms with valid signatures, detailed original records of drug distribution, etc., and the preservation period is 5 years. The ownership of all the data of this clinical trial belongs to the sponsor and co-sponsor. Except for the national drug regulatory authority, the investigator should not provide it to the third party in any form without the written consent of the sponsor.

12. Ethical Guidelines and Informed Consent Form

This clinical trial must be conducted in accordance with the Declaration of Helsinki (2008 Edition) and the relevant clinical trial study specifications and regulations in China. The study protocol should be developed before the initiation of the clinical trial. The study protocol should be discussed and signed by the investigator and the sponsor, and submitted to the Ethics Committee of the hospital for approval before implementation. During the actual implementation of this clinical trial, if it is necessary to revise this protocol, the revised trial protocol should be submitted to the Ethics Committee for approval before implementation. If important new information involving the investigational drug is found, the informed consent form must be revised in writing and submitted to the ethics committee for approval before obtaining the patient's consent again.

Before the initiation of the clinical trial, the investigator must provide the patient with detailed information about the clinical trial, including the nature of the trial, trial objectives, possible benefits and risks, and the patient's rights and obligations. The clinical trial can only be started after the patient has fully understood the informed consent and signed the "Informed Consent Form".

13. Quality control and quality assurance

- The clinical research unit shall be the drug clinical research base with clinical research conditions determined by CFDA;
- The investigators must be physicians trained in clinical trials and work under the guidance of senior professionals;
- The examination of clinical wards before the test must meet the standardization requirements to ensure that the rescue equipment is complete;
- The professional nursing staff will give medication to the subjects to have a detailed understanding of the medication and ensure the compliance of the subjects;
- Each study site must conduct the study in strict accordance with the study protocol and truthfully fill in the case observation form;
- The monitor should supervise the conduct of the clinical trial in accordance with the standard operating procedures, confirm that all data records and reports are correct and complete, and that all case report forms are correctly filled in and consistent with the original data, so as to ensure that the trial is conducted according to the clinical study protocol;
- In case of any SAE, the monitor shall timely report to all the study sites, and temporarily stop the study when necessary;
- All study sites participating in the trial should be audited by the sponsor and drug regulatory authorities. It is particularly important that the investigators and relevant personnel provide convenience and time for monitoring and auditing.

14. Study site and personnel

14.1 Study Site, Site Number, and Principal Investigator (Sort by Site Number)

Table 11 Relevant information of all study institutions

| Center No. | Study site | Principal Investigator |
| --- | --- | --- |
| 01 | Henan Cancer Hospital | Suxia Luo |
| 02 | The First Affiliated Hospital of Henan University of Science & Technology | Yanzhen Guo |
| 03 | Anyang Cancer Hospital | Junsheng Wang |
| 04 | Anyang People's Hospital | Heming Xi |
| 05 | Henan Provincial People's Hospital | Jianwei Zhou |

14.2 Leading Site and Leader

Single site: Henan Cancer Hospital

Address: No. 127, Dongming Road, Zhengzhou City, Henan Province

Post code: 450008

Responsible person: Suxia Luo

Tel: 0371 - 65587697

E-mail: 2290773710qq.com

15. Discussion, approval and modification of study protocol

The above "clinical study plan" and "clinical study case report form" are jointly determined by the principal investigators participating in the clinical trial of each unit after discussion, and can be implemented after being approved by Chia Tai Tianqing Pharmaceutical Group Co., Ltd. and approved by the Ethics Committee of the leading site. During the clinical trial, any amendment to the trial protocol should be reported to the Ethics Committee for approval or filing.

16. Test Summary

The principal investigator should summarize the results of statistical analysis of the trial in an objective and detailed manner, and actively complete the summary report of the clinical trial so as to meet the requirements of NMPA for unified regulation of clinical review of new drugs. Each participating unit should complete the sub-site summary.

Attachment 1 TNM Staging of Esophageal Cancer (Version 8, 2017)

T stage

Tis high-grade dysplasia

T1a tumors invade the lamina propria or mucosa

T1b tumor invades the submucosa

T2 Intrinsic tumor invasion

T3 tumor invading adventitia

T4a tumors invade adjacent structures, such as the pleura, pericardium, azygos vein, diaphragm, or peritoneum

T4b Tumor invades large adjacent structures, such as the aorta, vertebral body, or trachea

No regional lymph node metastasis at N0

N1 1-2 regional lymph node metastasis

N2 3-6 regional lymph node metastasis

N3 ≥ 7 lymph node metastases

M0 without distant metastasis

M1 distant metastasis

Gx differentiation cannot be determined

G1 well differentiated

G2 moderately differentiated

G3 Poorly Differentiated


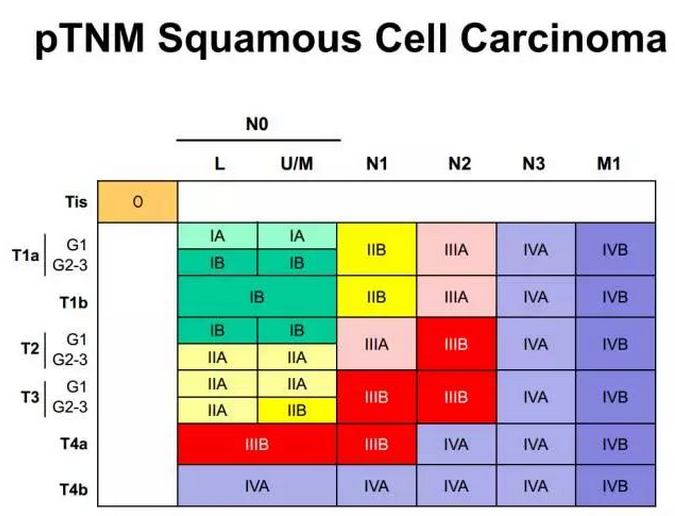


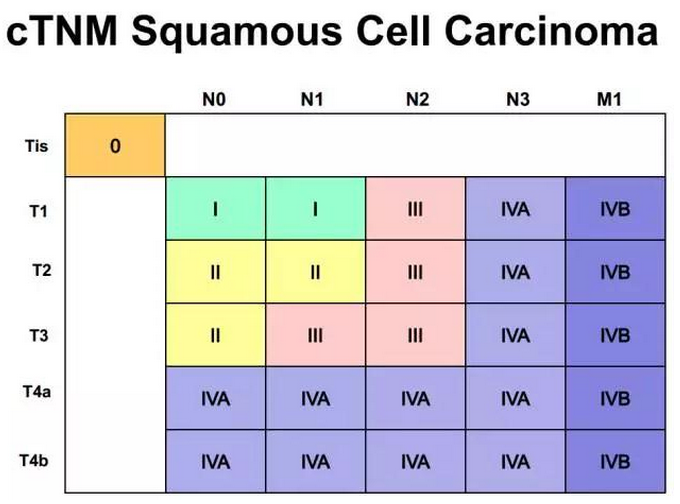


Appendix 2 Evaluation of quality of life (ECOG PS) (ZPS 5 scale)

| 0 | Normal activity |
| --- | --- |
| 1 | Mild symptoms, self-care, able to engage in light physical activity |
| 2 | Can tolerate the symptoms of the tumor and take care of himself/herself, but can stay in bed for no more than 50% of the time during the day |
| 3 | The tumor symptoms are severe, with more than 50% of the time in bed during the day, but they can also get up and stand, and some of them take care of themselves |
| 4 | Critically ill Bedridden |
| 5 | Death |

Appendix 3 New York Heart Association (NYHA) Functional Classification

| Grading | New York Heart Association (NYHA) Class |
| --- | --- |
| Grade I | No limitation of physical activity, ordinary activity does not cause undue fatigue, dyspnea or palpitation. That is, the compensatory phase of cardiac function. |
| Grade II | Slight limitation of physical activity. Asymptomatic at rest, fatigue, palpitation, dyspnea, or angina may be precipitated by ordinary activity. It is also known as grade I or mild heart failure. |
| Grade III | Marked limitation of physical activity, absence of symptoms at rest, less than ordinary activity producing the above symptoms. Also known as grade II or moderate heart failure. |
| Grade IV | Inability to engage in any physical activity, congestive heart failure or angina symptoms at rest, aggravated by any physical activity. It is also known as grade III or severe heart failure. |

Cardiac function is divided into four classes, and heart failure is divided into three degrees (slightly supplemented according to NYHA classification).

**Appendix 4 Creatinine Clearance Calculation**

**Cockcroft-Gault** Formula calculated creatinine clearance

Serum creatinine concentration (mg/dL):

| Creatinine clearance (mL/min) for males = | (140-age) × (weight) a |
| --- | --- |
|  | 72 × serum creatinine |

| Creatinine clearance (mL/min) for females = | 0.85 × (140-age) × (weight) a |
| --- | --- |
|  | 72 × serum creatinine |

Serum creatinine concentration (i.e. μmol/L):

| Creatinine clearance (mL/min) for males = | (140-age) × (weight) a |
| --- | --- |
|  | 0.81 × serum creatinine |

| Creatinine clearance (mL/min) for females = | 0.85 × (140-age) × (weight) a |
| --- | --- |
|  | 0.81 × serum creatinine |
